# An Overview of Marine Biodiversity in United States Waters

**DOI:** 10.1371/journal.pone.0011914

**Published:** 2010-08-02

**Authors:** Daphne Fautin, Penelope Dalton, Lewis S. Incze, Jo-Ann C. Leong, Clarence Pautzke, Andrew Rosenberg, Paul Sandifer, George Sedberry, John W. Tunnell, Isabella Abbott, Russell E. Brainard, Melissa Brodeur, Lucius G. Eldredge, Michael Feldman, Fabio Moretzsohn, Peter S. Vroom, Michelle Wainstein, Nicholas Wolff

**Affiliations:** 1 Department of Ecology and Evolutionary Biology, University of Kansas, Lawrence, Kansas, United States of America; 2 Washington Sea Grant, University of Washington, Seattle, Washington, United States of America; 3 Aquatic Systems Group, University of Southern Maine, Portland, Maine, United States of America; 4 Hawaii Institute of Marine Biology, Kaneohe, Hawaii, United States of America; 5 North Pacific Research Board, Anchorage, Alaska, United States of America; 6 Conservation International, Arlington, Virginia, United States of America; 7 Hollings Marine Laboratory, National Oceanic and Atmospheric Administration, Charleston, South Carolina, United States of America; 8 Gray's Reef National Marine Sanctuary, Savannah, Georgia, United States of America; 9 Harte Research Institute for Gulf of Mexico Studies, Texas A&M University – Corpus Christi, Corpus Christi, Texas, United States of America; 10 Department of Botany, University of Hawaii at Mānoa, Honolulu, Hawaii, United States of America; 11 National Oceanic and Atmospheric Administration Fisheries, Pacific Islands Fisheries Science Center, Honolulu, Hawaii, United States of America; 12 Consortium for Ocean Leadership, Washington, D. C., United States of America; 13 Bishop Museum, Honolulu, Hawaii, United States of America; 14 Joint Institute for Marine and Atmospheric Research, Honolulu, Hawaii, United States of America; Institut Pluridisciplinaire Hubert Curien, France

## Abstract

Marine biodiversity of the United States (U.S.) is extensively documented, but data assembled by the United States National Committee for the Census of Marine Life demonstrate that even the most complete taxonomic inventories are based on records scattered in space and time. The best-known taxa are those of commercial importance. Body size is directly correlated with knowledge of a species, and knowledge also diminishes with distance from shore and depth. Measures of biodiversity other than species diversity, such as ecosystem and genetic diversity, are poorly documented. Threats to marine biodiversity in the U.S. are the same as those for most of the world: overexploitation of living resources; reduced water quality; coastal development; shipping; invasive species; rising temperature and concentrations of carbon dioxide in the surface ocean, and other changes that may be consequences of global change, including shifting currents; increased number and size of hypoxic or anoxic areas; and increased number and duration of harmful algal blooms. More information must be obtained through field and laboratory research and monitoring that involve innovative sampling techniques (such as genetics and acoustics), but data that already exist must be made accessible. And all data must have a temporal component so trends can be identified. As data are compiled, techniques must be developed to make certain that scales are compatible, to combine and reconcile data collected for various purposes with disparate gear, and to automate taxonomic changes. Information on biotic and abiotic elements of the environment must be interactively linked. Impediments to assembling existing data and collecting new data on marine biodiversity include logistical problems as well as shortages in finances and taxonomic expertise.

## Introduction

An extensive global scientific initiative, the Census of Marine Life (Census) has assembled the first catalog of marine life, creating a baseline against which impacts of global change and human activity can be measured. The Census has been involved in examining previously unexplored marine ecosystems and in explaining the dynamic role of species over space and time. Some of the data gathered by Census projects have already provided vital information to policy makers and ocean educators to help preserve and protect marine resources, and will do so into the future. Conserving marine biodiversity will increase the ability of ecosystems to adapt and recover following natural or human-caused disturbances, including the impacts of global change in its many forms [1]. The Census has highlighted the importance of preserving natural marine biodiversity as a critical part of maintaining marine ecosystem functions and services, including fisheries, water quality, recreation, and shoreline protection [2].

**Figure 1:**
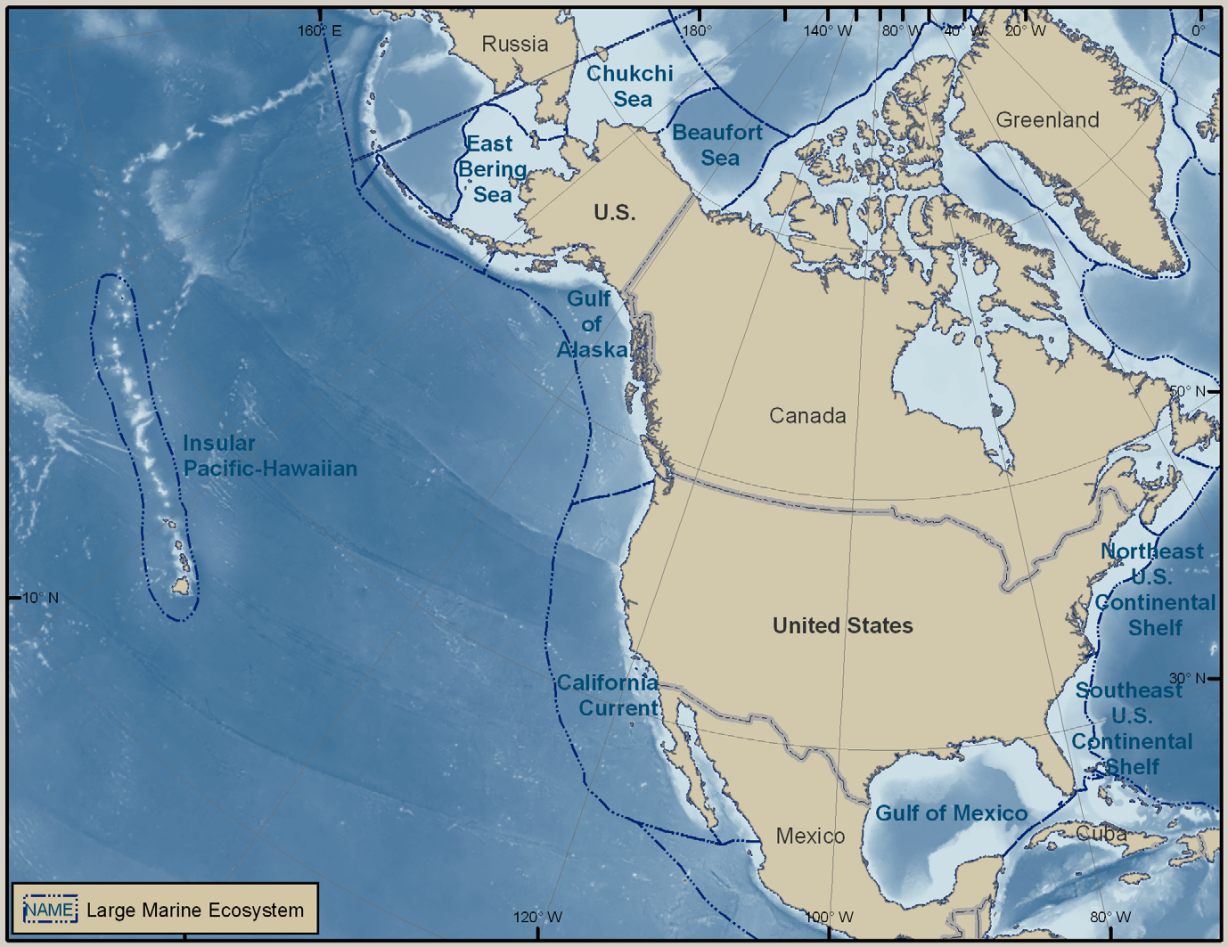
Map of six regions covered in the overview. The six regions are identified by the LME with which they coincide or of which they are a part: Northeast U.S. Continental Shelf, Southeast U.S. Continental Shelf, Gulf of Mexico, Insular Pacific–Hawaiian, California Current, Chukchi Sea, Beaufort Sea, East Bering Sea, and Gulf of Alaska.

This overview summarizes the knowledge—and some of the major gaps in knowledge—of marine biodiversity of the United States (U.S.) as of late 2009, when the data were assembled. The inventories and the summaries provided in Table S1 and its condensed version, Table 1, are at the species level, but there is discussion of biodiversity at ecosystem and genetic levels, which are also vital (e.g., [2]). Although it does not include information about regions administered by or associated politically with the U.S., the area that is covered is enormous, bordering on at least five major named bodies of water, and extending from 67° W to about 172.5° E, and from the tropics to the Arctic (just south of 19° N to 71° N). The regions differ greatly in history of exploration and knowledge of their biodiversity. Because the biota of a place such as Alaska may have more in common with that of Japan than with that of another part of the U.S., such as the Gulf of Mexico, a single list of marine species reported from the U.S. or an annotation that a species occurs in the U.S. is of little use for many scientific and management purposes. This overview is therefore divided into six geographically based sections (Figure 1), four of which are more or less coincident with large marine ecosystems (LMEs) (http://www.lme.noaa.gov). They are the Northeast U.S. Continental Shelf LME (#7), the Southeast U.S. Continental Shelf LME (#6), the Insular Pacific–Hawaiian LME (#10), and the Gulf of Mexico LME (#5). The last section covers the entire LME, not just that portion along the U.S. Gulf coast. The section on the West Coast concerns much, but not all, of the California Current LME (#3). The Alaska section includes part or all of four LMEs: the Chukchi Sea LME (#54), the Beaufort Sea LME (#55), the East Bering Sea LME (#1), and the Gulf of Alaska LME (#2).

**Table 1:** Biotic diversity of the six U.S. geographically-based sections in the text.

Taxonomic group	Northeast U.S. Continental Shelf LME	Southeast U.S. Continental Shelf LME	Gulf of Mexico	Insular-Pacific Hawaii LME	California Current LME	High Arctic (not exclusively the U.S.)
**Domain Archaea**	**UD**	**UD**	**UD**	**UD**	**UD**	**UD**
**Domain Bacteria (including Cyanobacteria)**	**10 (9)**	**48 (16)**	**UD (45)**	**UD (183)**	**UD**	**UD**
**Domain Eukarya**	**5,032**	**4,229**	**15,374**	**8,244**	**10,160**	**5,925**
**Kingdom Chromista**	**376**	**217**	**1,034**	**175**	**187**	**287**
Phaeophyta	154	217	86	84	187	
**Kingdom Plantae**	**246**	**113**	**967**	**821**	**703**	**150**
Chlorophyta	98	65	195	247	139	
Rhodophyta	148	38	392	574	557	
Angiospermae	UD	10	380	UD	7	
**Kingdom Protoctista (Protozoa)**	**51**	**165**	**2,169**	**798**	**896**	**759**
Dinomastigota (Dinoflagellata)	49		644	43	UD	70
Foraminifera	2	165	951	755	670	325
**Kingdom Animalia**	**4,359**	**3,734**	**11,150**	**6,395**	**8,374**	**4,729**
Porifera	36	111	339	144	134	163
Cnidaria	212	362	792	460	400	227
Platyhelminthes	77		705	676	1389	134
Mollusca	868	698	2455	1345	663	488
Annelida	689	400	866	343	830	533
Crustacea	810	696	2579	1325	2680	1525
Bryozoa	138	91	266	168	150	331
Echinodermata	138		522	309	290	151
Urochordata (Tunicata)	44	35	102	102	62	64
Other invertebrates	173	41	549	228	733	600
Vertebrata (Pisces)	954	1200	1541	1214	909	415
Other vertebrates	220	100	434	81	134	98
**TOTAL REGIONAL DIVERSITY** 3	**5,042**	**4,277**	**15,419**	**8,427**	**10,160**	**5,925**

Values are number of species^1,2^. See Table S1 for more details.

**Notes:**

1 Sources of the reports: databases, scientific literature, books, field guides, technical reports, and personal communication with taxonomic experts.

2 Identification guides cited in Text S2.

3 Includes all taxonomic groups as reported in Table S1.

UD  =  Listed in work but number undetermined to date; because taxonomic units in ICoMM are not species, they are not comparable to the data presented here and so are not included.

Each region is spatially defined, and features of its oceanographic setting that are known or likely to affect marine biodiversity are described. The approximate number of species in each major taxon is listed, with comments on taxa of particular note, including those that are commercially important or endangered. For example, the highest marine diversity ever recorded was on the slope east of Charleston in the Southeast U.S. Continental Shelf LME, and the Straits of Florida in that same LME has the richest ichthyofauna in the Atlantic. The Aleutian Islands has cold-water corals in very high diversity and abundance. Endemism of the Hawaiian biota is the highest of any tropical marine ecosystem on earth. Each section enumerates the threats to biodiversity in the region. As extensive as it is, it is clear from the biotic diversity inventories compiled in these six sections as an activity of the Census that knowledge of U.S. marine biodiversity is fragmentary. Knowledge is uneven spatially, taxonomically, and through time. Taxa that are best known – taxonomically, biologically, temporally, and in their geographical and ecological distribution – are no doubt those of current commercial importance, particularly finfish, or past commercial importance, such as whales and some birds. Knowledge diminishes with depth and with distance from shore, and with body size. A major challenge is to interrelate components assessed at genetic, species, habitat, and ecosystem levels.

The data come from a wide range of research efforts, including basic studies, monitoring programs that assess activities like fisheries, and exploration in preparation for activities such as oil production. The goal is to summarize patterns of biodiversity; secondary, and some primary, sources of information are cited in references. The Census has worked to assemble marine biodiversity data into an open-access database, the Ocean Biogeographic Information System (OBIS). Some of the data presented here are accessible through OBIS, the international site (http://www.iobis.org) and/or the U.S. node of OBIS, OBIS-USA (http://obisusa.nbii.gov).

### U.S. research capacity

Abundant information is available on U.S. marine waters, much a result of the large number of marine laboratories, vessels, and scientists in the U.S. According to the National Association of Marine Laboratories (NAML), an organization representing marine laboratories in the U.S. and its territories, more than 120 such laboratories support over 10,000 scientists, engineers, and professionals (http://www.naml.org/about).

**Figure 2:**
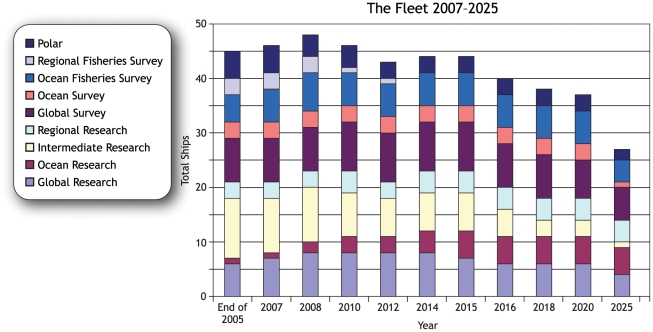
Federal fleet status projected through 2025. Figure courtesy of the Interagency Working Group on Facilities *Federal Oceanographic Fleet Status Report*. July 2007. Available at http://www.oceanleadership.org/files/IWG-F%20Fleet%20Status%20Report%20-%20Final.pdf

The hundreds of vessels used by marine laboratories, universities, and federal agencies range from small dinghies to global-class ships as long as 84 m. According to the Federal Fleet Status Report in 2007, 39 U.S. vessels were more than 40 m long (Figure 2). Figure 2 is projections based upon 2007 construction plans for the U.S. research fleet through 2025, organized by class and function. Since it was published, two vessels over 40 m in length (RV *Okeanos Explorer* and RV *Marcus Langseth*) have been added to the research fleet.

Many Census projects conduct research within U.S. waters. “Listening curtains” of the Pacific Ocean Shelf Tracking (POST) project track fishes of several species on migrations from Alaska to Baja, California, and Tagging of Pacific Predators (TOPP) has used marine life, including bluefin tuna and elephant seals, as oceanographers, fitting them with tags that collect data on temperature and salinity as they migrate, mate, and feed. The Natural Geography of Inshore Areas (NaGISA), which aims to inventory and monitor biodiversity in the nearshore zone of the world's oceans at depths less than 20 m, conducts research along many stretches of the U.S. coastline, notably in Alaska and New England. Many of the scientists involved in the Arctic Ocean Diversity (ArcOD) project, which studies waters of the Arctic Ocean, are at the University of Alaska, Fairbanks. In the Gulf of Mexico, researchers of the Continental Margin Ecosystems on a Worldwide Scale (COMARGE) study areas of the continental margin that are relatively untouched by commercial exploitation. The Census of Coral Reef Ecosystems (CReefs) is an international cooperative effort to assess, visualize, and explain diversity patterns, including those of the coral reefs of the Hawaiian Islands, before such patterns are further affected by global change. The Gulf of Maine Area (GoMA) program documents patterns of biodiversity and related processes so its findings can be used to establish ecosystem-based management (EBM) of the Gulf of Maine (Table 2). The International Census of Marine Microbes (ICoMM: http://icomm.mbl.edu/microbis/) has inventoried marine microbial diversity (inclusive of the Bacteria, Archaea, Protista, and associated viruses) in numerous places in U.S. waters. Included in its activities is Visual Analysis of Microbial Population Structures (VAMPS) at http://vamps.mbl.edu.

**Table 2:** Census of Marine Life projects by Large Marine Ecosystem.

	Alaska Region	California Current	Gulf of Mexico	Southeast U.S. Continental Shelf	Northeast U.S. Continental Shelf	Insular Pacific– Hawaiian
Census Project	LMEs # 1, 2, 54, 55	LME #3	LME #5	LME #6	LME #7	LME #10
ArcOD	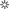 ▪ ▴	n/a	n/a	n/a	n/a	n/a
CeDAMar	n/a	n/a	n/a	n/a	▪	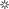 ▪
ChEss	n/a	▪	▪ ▴	n/a	n/a	n/a
COMARGE	n/a	▪ ▴	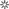 ▪ ▴	n/a	n/a	n/a
GoMA	n/a	n/a	n/a	n/a	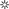 ▪ ▴	n/a
MAR-ECO	n/a	n/a	n/a	n/a	▪	n/a
POST	▪ ▴	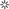 ▪ ▴	n/a	n/a	n/a	n/a
CAML	n/a	n/a	n/a	n/a	n/a	n/a
CenSeam	n/a	n/a	n/a	n/a	▪	n/a
CMarZ	▪	▪	▪	▪	▪	▪
CReefs	n/a	n/a	n/a	n/a	n/a	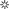 ▪ ▴
ICoMM	▪	▪	▪	▪	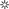 ▪	▪
NaGISA	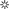 ▪	▪	▪	▪	▪	n/a
TOPP	▪	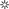 ▪ ▴	▪ ▴	n/a	n/a	▪ ▴
FMAP	▪	▪	▪	▪	▪	▪
HMAP	n/a	n/a	n/a	n/a	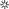 ▪ ▴	n/a
OBIS	▪	▪	▪	▪	▪	▪

**Note**: 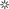  =  Census project's primary area of study; ▪  =  Census studies in the region; ▴ =  Intensive and integrated studies of biodiversity; N/A  =  No Census work in the region. Please see Text S1 for full project titles.

### The importance of biodiversity

Meetings organized by the Census U.S. National Committee for members of academic, government, and not-for-profit organizations have addressed topics concerned with how biodiversity can be assessed. A major premise of Census activities in the U.S., that maintaining biodiversity is a worthy goal, accepts the assertion [3] that the survival and well-being of humans depend on intact, fully functioning ecosystems. Further, conservation of biodiversity for its own intrinsic value, above and beyond consideration of human needs, should be a significant and recognized goal of global society [4].

In the marine environment and elsewhere, a growing body of evidence relates the maintenance of healthy, natural biodiversity to provision of a broad spectrum of ecosystem services, including those that humans rely upon and value, such as food, medicines, recreation, climate modulation, and protection from extreme weather [2], [3]. However, at a global scale, 60% of ecosystem services are degraded [3]. Along U.S. coasts, loss or impairment of biodiversity correlates with degraded ecosystem services important to humans [5]. Specifically, there are impacts to tourism, loss of aesthetic and other cultural attributes, lowered property values, and increased health risks to humans and animals from harmful algal blooms and their toxins, infectious disease organisms, and chemical contaminants [6], [7]. Efforts to develop national marine spatial planning as a component of national ocean policy will be an important advance in the efforts to conserve marine biodiversity [8].

The U.S. Commission on Ocean Policy (USCOP) [9] identified EBM as a cornerstone of ocean policy reform and specifically stated that conservation of natural biodiversity was a crucial part of EBM. The USCOP report said, “One of the central goals for ecosystem-based management should be the explicit consideration of biodiversity on species, genetic, and ecosystem levels…[I]t is now understood that every species makes some contribution to the structure and function of its ecosystem. Thus, an ecosystem's survival may well be linked to the survival of all the species that inhabit it.” The Joint Ocean Commission Initiative (JOCI) [10] reemphasized the importance of EBM, including the conservation of biodiversity, in recommendations to President Barack Obama's administration. An extensive discussion of implementing EBM for the marine environment is presented in McLeod and Leslie [11].

According to the 2008 Valencia Declaration [12], “Marine biodiversity underpins the functioning of marine ecosystems and their provision of services—without biodiversity there would be no ecosystem services.” While the ecological mechanisms linking biodiversity to sustained marine ecosystem function are not fully understood, it is clear that preserving biodiversity could be one important way to maintain continued provision of critical ecosystem services, including fisheries, water quality, and others [2], [6]. Because sustained biodiversity is likely to benefit most ecosystem services, Palumbi et al. [2] suggested that an EBM approach focused on conservation of natural biodiversity would benefit sectoral management and enhance the resiliency of coastal ecosystems and the human communities associated with them. Maintaining and improving coastal resiliency is important with regard to effects of ecosystem alterations, especially those that may occur as inadvertent consequences of human behavior.

A wide range of human activities affect marine biodiversity both in direct ways, such as exploitation by fisheries, habitat loss due to dredging, filling, and other construction influences, fishing gear impacts, and pollution, and in less direct ways, including effects of global change resulting in acidification, warmer waters, and coastal inundation. Some activities cause biodiversity loss due to ecosystem changes (e.g., reduced coral reef health commonly is associated with reduced populations or even extirpation of some organisms), whereas others may change ecosystem function because of alterations in diversity - either up or down (e.g., due to invasive species). Rising atmospheric carbon dioxide not only contributes to temperature increase but, as some of that carbon dioxide dissolves in the ocean, pH of ocean water declines. A growing number of studies have demonstrated adverse impacts on marine organisms, including decreases in rates of coral calcification, reduced ability of algae and zooplankton to maintain protective shells, and reduced survival of larval marine shellfish and fish [13], [14], [15]. Ecosystems can undergo rapid change in their ability to provide a range of ecosystem services as biodiversity changes [6].

To monitor and evaluate changing biodiversity, and develop policy responses to it requires developing reference levels of biodiversity currently and, as far as is possible, into the past (e.g., [16]). This overview is intended to summarize the current state of knowledge for the U.S., where relatively few management measures for protecting marine biodiversity have been implemented.

## Results

### Northeast United States Continental Shelf Large Marine Ecosystem

#### Description of the Northeast Continental Shelf region

**Figure 3:**
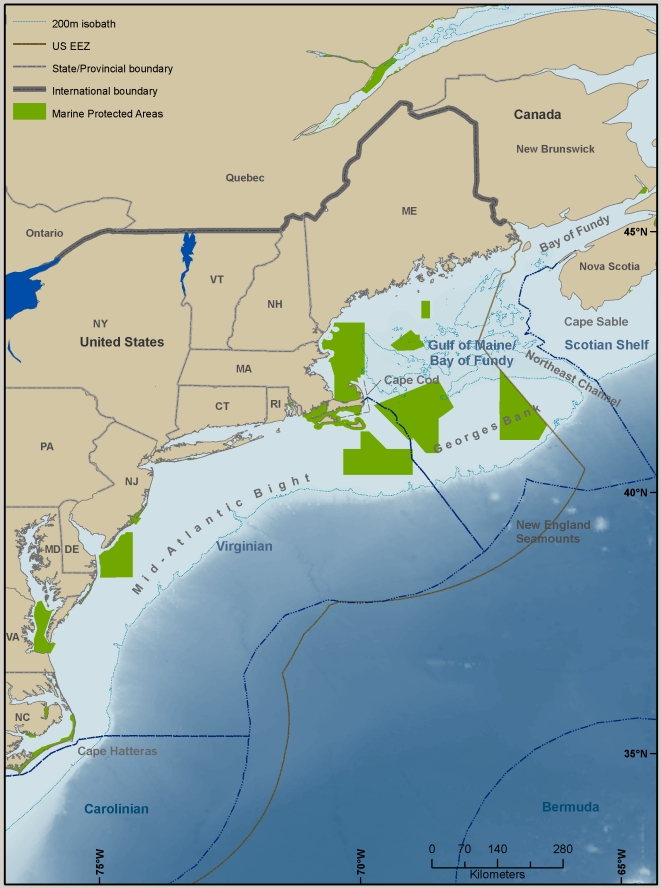
Virginian and Gulf of Maine/Bay of Fundy Ecoregions, showing bathymetry and major geographic landmarks. The New England Seamounts are those extending south from Georges Bank. Only the larger protected areas are shown, and they have different goals and levels of protection.

The Northeast U.S. Continental Shelf LME extends more than 3,000 km from Cape Hatteras, North Carolina, into Canadian waters of the Gulf of Maine (Figure 3) [17]. It includes the Bay of Fundy, Northeast Channel, and all of Georges Bank. The LME includes two biogeographic provinces historically divided at Cape Cod: the Virginian Province extends south to Cape Hatteras, and the Acadian Province extends north to the Gulf of St. Lawrence [17]. While many studies have corroborated Cape Cod as a shoreward “boundary” between these provinces (see Wares [18] for discussion), the extension of this demarcation to offshore habitats is not straightforward. Depending on the faunal and environmental comparisons being made, some studies have drawn a connection between Georges Bank and the northern Mid-Atlantic Bight to the south, while others claim that Georges Bank is distinct from both the Gulf of Maine and regions to the south (Theroux and Grosslein [19] discuss similarities between Georges Bank and the shelf region south of it; Longhurst [20] and others discuss the distinctness of Georges Bank; Cook and Auster [21] provide a thorough discussion). Despite the distinctions between them, Georges Bank and the Gulf of Maine are generally recognized as a closely coupled system. A recent description of world marine coastal ecoregions [22] places the northern border of the Virginian Province at a line extending from Cape Cod southeastward across the shelf, thus assigning Georges Bank to the northern sector. The study also divides the Acadian Province into a Scotian Shelf ecoregion (in the north, not further considered in this paper) and a Gulf of Maine/Bay of Fundy ecoregion (henceforth referred to as the GoM ecoregion), whose northern border of which follows the 100 m isobath south of Nova Scotia (Figure 3). This is close to the border that defines the Northeast U.S. Continental Shelf LME.

**Figure 4:**
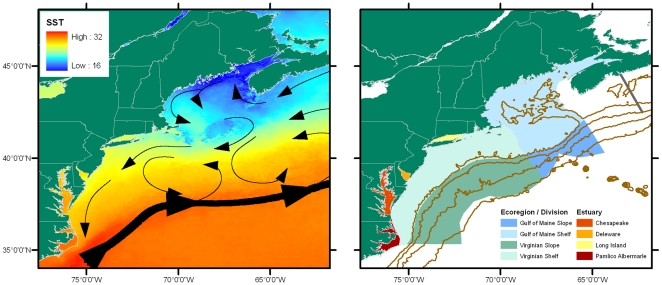
Detail of the study area. Right panel shows shelf and slope areas used in Table 3. The line extending across the shelf and upper slope (“Halifax Line”) northeast of the Gulf of Maine marks the eastern end of the Gulf of Maine Area Program of the Census of Marine Life. The major bays and estuaries of the Virginian ecoregion are also shown. Left panel shows climatological sea surface temperature for August, 1985-2001 (data are from U.S. NASA Pathfinder mission, 4 km resolution). Arrows show schematic of the prevailing residual circulation (after Townsend et al. [22]).

The GoM ecoregion has a strong subboreal affinity due to the northerly sources of water entering the gulf, and restricted exchange with the neighboring ocean (Figure 4). Water temperatures can be cooled to less than 5-6°C down to 100-150 m depth by winter convection [23], [24], and surface temperatures can be slow to warm in spring. Parts of the gulf remain cool throughout the year due to strong tidal mixing and advection of cold water in a cyclonic (counterclockwise) coastal current system [25] (Figure 4). Where waters become stratified during warm months, surface temperatures exceed 20°C, while the bottom layer remains cold. Parts of the gulf are deep enough to maintain an intermediate water layer conditioned by winter mixing and isolated by seasonal warming at the surface [23].

The environmental transition from GoM to Virginian ecoregions is fairly pronounced due to a generally steep latitudinal gradient in atmospheric forcing as well as topographical influences on circulation. The cyclonic circulation of the GoM and the anticyclonic flow around Georges Bank adds to the residence time of water before it arrives south of Cape Cod (Figure 4). In the Virginian ecoregion, stratification and surface warming begin earlier in the year, and summer temperatures exceed 20°C for many months [26]. Residual circulation over the shelf is southward, thus reducing the tendency for planktonic propagules to be transported northward into the GoM ecoregion. However, the ranges of many sedentary species do extend northward both along the coast and offshore, and species show a variety of patterns of distribution indicative of differences in spreading potential, invasive opportunities, past conditions, and life history requirements [27].

Temperatures in the neighboring deep ocean are moderated by strong northward transport of heat in the Gulf Stream, which begins to veer eastward before reaching Georges Bank. Warm core rings occasionally transport warm-water species to Georges Bank and the Gulf of Maine [28], [29], but these organisms are generally out of their temperature tolerances range during winter. Slope water systems from the north (Labrador Sea) and south (Mid-Atlantic Bight) also meet in the region off Georges Bank and affect not only the slope itself, but also the temperature, salinity, and nutrient content of waters entering the deep Gulf of Maine through the Northeast Channel [25], [30]. The latitudinal thermal gradient over the continental shelf in this region is extremely steep, and thus is of particular interest for monitoring biodiversity changes in a warming climate.

The Gulf of Maine is topographically and geologically complex, containing three major basins up to 300 m deep, and numerous smaller basins, sills, and ridges. The shoreline is also diverse, consisting of extensive regions of metamorphic and igneous rock, as well as sandy and gravelly shorelines of various lengths. Marshes, small and sparse in rock-dominated sections of the coast, are extensive along some sections of sandy coast. Rocky sections are typically highly indented, with numerous bays, peninsulas, and islands providing a wide variety of exposed and protected habitats. The tidal range varies from about 3 m in the south to 16 m in the northeastern Bay of Fundy (Minas Basin), reputedly the largest amplitude in the world [31]. In the north, and over the crest of Georges Bank, strong tidal shear contributes to unstratified or only weakly stratified conditions even during warm months of the year. The large, shallow banks (Nantucket Shoals, Georges Bank, and Browns Bank) restrict exchanges between the Gulf of Maine and the open Atlantic, and create a marginal sea distinctive from the relatively smooth and open continental shelf to the south.

From Cape Cod to Delaware Bay, the shoreline is characterized by mixed sandy and rocky regions, and it is predominantly sandy deposits south to Cape Hatteras. The shelf is generally wide and gently sloping, but narrows in the south at Cape Hatteras, which is close to the Gulf Stream. Unlike the Gulf of Maine, the Virginian Province has several large, shallow estuarine systems with comparatively narrow connections to the coastal ocean: Delaware Bay, Chesapeake Bay, and the Pamlico/Albemarle Sound system (Figure 4). Together with Long Island Sound and Narragansett Bay, these estuarine or estuary-like systems contribute significantly to the total area and variety of coastal habitats along the eastern U.S. continental shelf.

The continental slope in the north is cut by numerous submarine canyons, remnants of drainages that formed during periods of lower sea level. South of Cape Cod they are fewer, with the notable exceptions of the large Hudson and Baltimore Canyons. On the mid- to lower slope south of Georges Bank, and within the U.S. Exclusive Economic Zone (EEZ), are four seamounts of the New England Seamount Chain. These rise to summit depths ranging from –1,200 m to –2,800 m, or within the depth range of the slope. Bear Seamount, the closest and shallowest, is 65 km from the shelf break. The seamount chain extends a long distance toward the Corner Rise Seamounts and the Mid-Atlantic Ridge system, possibly serving as a series of faunal stepping-stones.

#### Regional history of biodiversity studies

The U.S. Fish Commission research vessel *Fish Hawk* conducted the earliest intensive surveys of the biology of the outer continental shelf in 1880, sampling with bottom dredges and trawls, opening an era of investigation of shelf sea species and their distributions, collecting many new taxa of mollusks and fishes. In 1882, *Alabtross* was launched as the first large vessel of any nation built expressly for marine research. Both vessels were used in extensive biological and environmental surveys of the North American East Coast from Newfoundland (*Albatross*) and the Gulf of Maine (*Fish Hawk*) to Florida and elsewhere. In 1912, Henry Bryant Bigelow began his pioneering studies of the Gulf of Maine in a joint academic and federal fisheries investigation. Bigelow's unique contribution to science was to combine measurements of temperature, salinity, plankton, circulation (drift bottles), and fishes to develop a holistic view of the ocean and the life it supports. “Nothing in the sea falls haphazard,” he wrote in a 1929 report to the National Academy of Sciences, “if we cannot predict, it is because we do not know the cause, or how the cause works.” From his work in the Gulf of Maine (1912-28) he published treatises on the fishes [32], the plankton [33], and the physical oceanography [34]. The original *Fishes of the Gulf of Maine*, written with William W. Welsh (posthumously) [32], has become a classic. It was updated in subsequent editions by Bigelow and Schroeder [35] and Collette and Klein-McPhee [36]. The latest edition includes 118 families and 252 species, with extensive information describing the organisms, their biology, general range, and distribution in the Gulf of Maine. The treatises on physical oceanography and the plankton continue to be used for comparative purposes. Fahay [37] described the ichthyoplankton of the region from Cape Hatteras to the Scotian Shelf in an illustrated guide to 290 species likely to be collected by plankton or neuston nets, including some oceanic mesopelagic and bathypelagic forms.

Scientific sampling of coastal intertidal and shallow subtidal organisms extends back to the mid-1800s [38], [39]. One area of early focus was Cobscook Bay, near the Canadian border, where the diversity of physical habitats and the large tidal range probably contribute to its diverse invertebrate assemblages. In a historical checklist of marine invertebrates from the bay (an area of only 110 km^2^) spanning 162 years, Trott [39] listed nearly 800 species in 17 phyla. Cobscook Bay and neighboring Passamaquoddy Bay are subjects of joint U.S. and Canadian Census studies of intertidal and shallow subtidal communities (NaGISA), and the history of nearshore ecosystems (HNS). The Gulf of Maine Area Program of the Census includes the GoM ecoregion, plus the southern and western Scotian Shelf, the continental slope to 2,000 m, and the western New England Seamounts.

#### The known, unknown, and future directions

The Census Gulf of Maine Area Program has developed a Gulf of Maine Register of Marine Species (GoMRMS) in collaboration with the Huntsman Marine Science Center, Canada. GoMRMS is a provisional list of taxa based on (1) a previous dataset from the Bay of Fundy (1,408 species); (2) a compilation of species collected in the Gulf of Maine area based on museum specimen holdings, research and survey cruises, and published reports; and (3) species *expected* in the Gulf of Maine area based on published faunal lists and ranges from the Canadian Atlantic RMS. The Canadian Atlantic Register of Marine Species (CARMS) includes species from the Canadian and U.S. Atlantic as far south as Cape Hatteras. Taxonomic updating and validation of GoMRMS are ongoing. Details can be found at http://www.marinebiodiversity.ca/.

The area covered by GoMRMS is the same as the GoMA study area (defined above), with three modifications. GoMRMS also includes a portion of Nantucket Shoals and Nantucket Sound south of Cape Cod; on the slope it includes depths to 1,000 m; and it does not include the seamounts. As of 12 November 2009, GoMRMS listed 3,141 species, about 31% of them validated for updated taxonomy, occurrence, and other documentation (the percentage varies by group). Because GoMRMS was designed to support GoMA, it extends beyond the GoM ecoregion as shown in Figure 4, thus including the Scotian Shelf ecoregion of Spalding et al. [22]. The list of fishes (504 species) includes mesopelagic species from research cruises off the Atlantic coast of Nova Scotia. By contrast, the fishes listed by Collette and Klein-McPhee [36] for the GoM include 252 species, only a third of which are permanent residents, the remainder seasonally migrating (in descending rank order) from the south, from deep water, and from the north.

There is no readily comparable register of species for the Virginian ecoregion. We constructed a preliminary list using regional registers from the Canadian Center for Marine Biodiversity. All species occurring between Davis Strait (Canadian Arctic) and Cape Hatteras (contained in the Northwest Atlantic Register of Marine Species) minus those that occur from Davis Strait to the southern Gulf of Maine (Canadian Atlantic RMS) amount to 952 species *unique* to the Virginian ecoregion. Adding this number to the species in GoMRMS gives a total of 4,093 register species for the two marine ecoregions covered in this section (Table S2). The relatively small number of additions ascribed to the south is the result of a northern bias in the register work to date, and is not a reliable reflection of biodiversity patterns.

We examined three survey databases for potential additions to the species already accounted for in the registers. Two databases from the National Marine Fisheries Service (NMFS)/Northeast Fisheries Science Center are the demersal trawl survey database and the benthic fauna database. The trawl surveys are directed at the assessment of living marine resources and the management of fisheries. Using standardized methods, they have been conducted two or more times per year since the 1960s [40], [41]. The benthic database contains records obtained using a variety of methods, mainly in the 1960s and early 1970s [42]. For this analysis, samples were restricted to three methods that were applied in a similar manner in both ecoregions. Sampling methods were (1) for shelf depths less than 200 m, the fisheries demersal trawls (as above) and 1 m^2^ Campbell and 0.25 m^2^ Smith-McIntyre grabs [42], [43], [44], [45]; and (2) for the upper slope, a Campbell grab (as above). Sample size for the slope was small, but we include the data for interest. While both databases date back to the 1960s, the demersal trawl survey data form the only systematic time series. A third database is from a series of surveys of coastal waters (mostly near shore) and major bays and estuaries along the Atlantic coast of the U.S., conducted by the U.S. Environmental Protection Agency (EPA) as part of its National Coastal Assessment (NCA) from 1990 to 2004 [46], [47]. Samples were obtained with small trawl nets and modified Van Veen grabs (0.04 m^2^, 0.5 mm sieve).

Samples from these databases were selected so that they came only from the GoM and Virginian ecoregions as shown in Figure 4. All sampling methods were used to add to the species list, but only the grab samples were used to compare the two ecoregions because not many trawl samples were available from the Gulf of Maine. The NCA database includes sampling sites that were located far up some estuaries and rivers, but we used data only from stations along the coast and near the mouths of large bays. All three of the databases are either in OBIS or there are plans to submit the data to OBIS in the near future. Analysis was completed in November 2009.

After using the World Register of Marine Species (WoRMS) to vet species names, we compared the list of species found in the three databases with the combined GoM and Virginian list from the registers. Species appearing in our search of the databases but not in the registers represent 952 provisional additions to the named species from the Northeast Continental Shelf, and a grand total of 5,045 named species. Results are summarized in Table 3, and more detail is provided in Table S1 and Table S2. About 5% of the species could not be vetted by WoRMS at the time of analysis, so some errors of synonymies and identification may exist.

**Table 3:** Biotic diversity in the Northeast U.S. Continental Shelf Large Marine Ecosystem.

Taxonomic group	Total no. species^1,2^	Total in registers	Provisional additions from databases
**Domain Archaea**	**UD**		
**Domain Bacteria (including Cyanobacteria)**	**10 (9)**	10	
**Domain Eukarya**	**5,032**		
**Kingdom Chromista**	**376**		
Phaeophyta	154	154	
**Kingdom Plantae**	**246**		
Chlorophyta	98	98	
Rhodophyta	148	148	
Angiospermae	UD		
**Kingdom Protoctista (Protozoa)**	**51**		
Dinomastigota (Dinoflagellata)	49	49	
Foraminifera	2		2
**Kingdom Animalia**	**4,359**		
Porifera	36	32	4
Cnidaria	212	192	20
Platyhelminthes	77	76	1
Mollusca	868	687	181
Annelida	689	445	244
Crustacea	810	549	261
Bryozoa	138	76	62
Echinodermata	138	73	65
Urochordata (Tunicata)	44	42	2
Other invertebrates	173	140	33
Vertebrata (Pisces)	954	877	77
Other vertebrates	220	220	
**SUBTOTAL**	**5,042**	4,090	952
**TOTAL REGIONAL DIVERSITY** 3	**5,045^#^**	4,093	952

**Notes:** Summary of named species from regional registers and provisional additions from three regional databases as of November 2009 (see text for details).

1 Sources of the reports: databases, scientific literature, books, field guides, technical reports, and personal communication with taxonomic experts.

2 Identification guides cited in Text S2.

3 Includes all taxonomic groups as reported in Table S1.

UD  =  Listed in work but number undetermined to date.

#  =  three unclassified Protoctista species bring the sub-total (5,042) to the total diversity (5,045).

We also examined the databases for species occurring in one or both ecoregions (summarized in Table 4). The data show more than twice the number of fish species in the south (364 vs. 154 demersally caught species). Also, relatively few species are unique to the GoM, while a large number are unique to the Virginian region. [Note: these are species caught by demersal trawls, and not the full species list.] For the benthic invertebrates on the shelf, sampling effort was not equally distributed by method between the two ecoregions (a potential source of bias), and only the pooled results are shown. In the pooled assessment, the southern ecoregion showed a third more total species and more than twice as many unique species. The nearshore data show similar patterns. The slope regions contain a small number of samples and a little over 100 identified species in each ecoregion, about half of which were unique to that region. Taxonomic details of the database findings are given in Table S3. The three databases contain 2,356 species, providing spatial information on nearly half of the total named species (n = 5,045, Table 3) in this report. This assessment provides only a preliminary view of the information, and more detailed analysis is needed. Many species that overlap the two ecoregions on the shelf do so primarily in the southern Gulf of Maine and Georges Bank, and species occurrences do not reflect patterns of abundance or dominance. Ecoregion size and sample sizes have not been factored in.

**Table 4:** Species from demersal fish and benthic surveys using the same methods across both the Gulf of Maine/Bay of Fundy and the Virginian ecoregions of the Northeast U.S. Continental Shelf Large Marine Ecosystem.

	Ecoregion	Fish - Shelf	Invertebrates - Shelf	Invertebrates – Upper Slope
**I. Species numbers**	GoM only	9	220	49
	Both	145	619	59
	Virginian only	219	499	59
**II. Number of samples**	GoM	5,579	2,091	42
	Virginian	6,973	3,079	94

**Notes:** The numbers are species unique to each ecoregion, or that occur in both.

Other sources of data, such as East Coast plankton surveys will add to this list over time, but they are not as accessible at present and some are regional in nature. The EPA/NCA database includes many estuarine samples that we did not include here. North of our study area are two other sources of spatially explicit biodiversity data worth mentioning. These are trawl surveys, comparable to the U.S. survey, conducted by the Canadian Department of Fisheries and Oceans and also available through OBIS, and a species compilation for the Gulf of St. Lawrence, which is divided into spatial subunits [48]. The diversity of marine invertebrates has been published [48](2,214 species), and data for all taxa are available upon request (http://www.qc.ec.gc.ca/faune/biodiv/en/methods/data_access.html). Other significant databases containing biodiversity information are listed in Table S4; Text S2 provides a list of useful taxonomic and field guides.

Underused databases represent a wealth of potential information, but are still far from complete. Effective stewardship of marine resources requires new sampling capabilities in remote, as well as accessible, parts of the sea. Exploration requires tools for rapid quantitative measurements at scales from small to very large. Two recent developments in the northeastern U.S. illustrate these two ends of the spectrum. At the small to medium scale, imaging and image-processing technology are opening up opportunities for rapid assessment of benthic communities [49]. At the large scale, new acoustic sampling technology is enabling long-distance, synoptic, and high-frequency observation of the density, distribution, and behavior of large schools of fish [50], [51]. Both are rooted in the basic exploration of patterns in nature, but have potentially important applications for understanding and managing human impacts on marine ecosystems. The establishment of marine and shoreland protected areas, long-term ecological research sites, and other types of natural heritage sites provides means for conserving and studying biodiversity, as well as educating the public about the aesthetic and practical benefits of managing for biodiversity maintenance [52].

#### Trouble spots and emerging issues

Virtually all of the issues cited below are common to other regions of the U.S. and many other countries. We focus on providing citations for recent work in our region.

The greatest immediate threats to regional marine biodiversity are the direct and indirect effects of fishing, which has been conducted at intense, industrial levels for half a century or more. Fishing impacts on biodiversity include severe reductions in upper-trophic-level predators and cascading responses through lower parts of the food web [53], [54], [55]. Selective removals (spatially or by size) can have genetic and population structural effects, with consequences for resilience and adaptation of the populations, and fishing activities alter the structure of bottom communities [56], [57]. The entire region, but particularly the northeast, is faced with the challenge of sustaining or restoring productive and economically viable fisheries while also taking meaningful steps to conserve biodiversity and the ecosystem functions and adaptability that depend on it.

A second challenge, now increasingly apparent in the region, is global change, which brings alterations in temperature, circulation, acidification, and sea level. Rising temperatures and shifts in organism distributions, phenology, and composition have been noted by numerous authors [26], [58], [59], [60], [61], and acidification threatens many important components of the regional biota [14], [62].

Along the coast, the effects of human encroachment and activity on coastal habitats reduces the amount of natural space, introduces pollutants, and impedes natural adjustments to the shore as sea level increases [63]. Eutrophication and hypoxia have become problems in more densely populated regions [64], [65], and seagrass communities in the northeast have been in decline, in keeping with a worldwide trend [66]. Invasive species, of which there are now many in eastern U.S. waters, represent yet another way that natural biodiversity of the region is being altered by human activities.

#### The Census of Marine Life contribution to the Northeast Continental Shelf region

To improve understanding of patterns and processes that support marine biodiversity, the Census program has focused attention on the extensive task of documenting what lives in the ocean. The Gulf of Maine Register of Marine Species (GoMRMS) developed by the Census Gulf of Maine Area Program represents a major advance in compiling biodiversity information for this region.

### Southeast United States Continental Shelf Large Marine Ecosystem

#### Description of the South Atlantic Bight and Florida East Coast region

**Figure 5:**
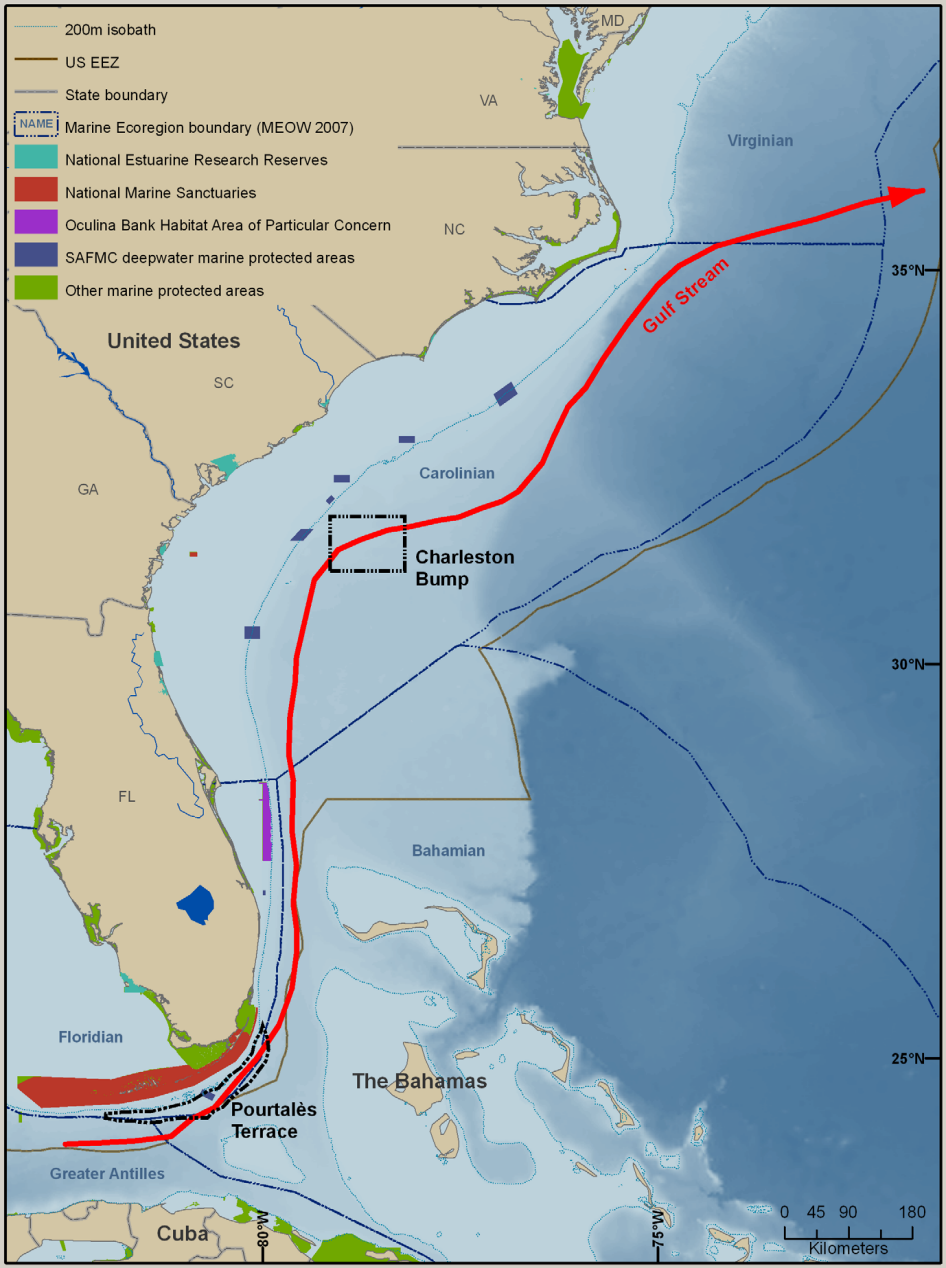
The SAB-Florida East Coast Large Marine Ecosystem. The large red arrow represents the Gulf Stream.

The region includes marine habitats from Cape Hatteras, North Carolina to West Palm Beach, Florida, an area often referred to as the South Atlantic Bight or SAB [67]), plus the remaining southeast coast of Florida, including the Florida Keys (Figure 5). This large, complex, and diverse area, with Cape Hatteras, North Carolina and Cape Canaveral, Florida as well-recognized zoogeographic boundaries, extends seaward to the limit of the U.S. EEZ. Thus, it includes areas beyond the 200 m depth contour (generally considered the outer boundary of coastal and shelf realms, provinces, and ecoregions [22]) and encompassing the waters of the continental slope, Blake Plateau, and the Straits of Florida. For the purposes of this summary, the area considered will be called the SAB-Florida East Coast. For comparison with other biogeographic descriptions, it includes part of the Temperate North Atlantic Realm that contains the Warm Temperate Northwest Atlantic Province, and within that the Carolinian Ecoregion, and the subtropical zone (northern quarter and western Atlantic portions) of the Western Central Atlantic area (Fishing Area 31) of the Food and Agriculture Organization (FAO) of the United Nations.

Circulation, hydrography, and most marine habitats and ecosystems of the SAB-Florida East Coast have been well studied (see Atkinson and others [67], [68], [69] for useful summaries and citations of additional reviews and original studies). The region is dominated hydrographically by the Gulf Stream, which has a major influence on the fauna. Bottom type and topography are also important determinants of diversity, as well as having an influence on circulation and recruitment of larvae. The Gulf Stream is formed from the Florida Current that originates in the tropical Atlantic. The Florida Current, in turn, originates in the Gulf of Mexico as the Loop Current and along the Eastern Caribbean as the Antilles Current. Counter currents and gyres created by bottom features such as the Pourtalès Terrace in the Florida Current and the Charleston Bump in the Gulf Stream help retain the pelagic larvae within the area of the Florida Keys and SAB, respectively, by causing persistent gyres and eddies that spin off the current and retain their pelagic flora and fauna, some of which recruit to benthic and pelagic habitats in the region. The Carolina capes and their seaward shoals, along with the broad shelf (up to 200 km), also influence Gulf Stream flow and result in retention of water masses and their pelagic larvae in gyres between the capes. The Virginia Current, originating in the Labrador Current to the north, flows southward along the middle Atlantic states of the U.S. and occasionally rounds Cape Hatteras, bringing cooler water and larvae of cold-temperate species into the region, where they become temporary or longer-term residents. The region thus has a high diversity of cold-temperate, warm-temperate, and tropical species.

The warming influence of the Gulf Stream in the SAB is especially notable from January through March near the shelf break, where tropical species of fishes, corals, and other animals are found. A warm band of relatively constant temperature (18-22°C) and salinity (36.0-36.2 psu) is observed near the bottom year-round just inshore of the shelf break, bounded by seasonally variable waters on the inshore side, and by fluctuating waters subject to cool-water upwelling events and warm Gulf Stream intrusions on the offshore side.

Fresh water input in the SAB is supplied mainly by the Cape Fear, Pee Dee, Santee, Savannah, and Altamaha rivers. In South Florida, land reclamation and water management projects have diverted most flow from the Everglades (which formerly flowed into Florida Bay) into the Atlantic and Gulf of Mexico. River runoff is highest during March and April, and tropical weather systems provide additional freshwater input from June through October, particularly in South Florida. Seasonal heating and cooling of coastal and shelf waters follow a trend in air temperature increase and decrease, with a lag of approximately one month.

Semidiurnal tides dominate, the range varing considerately because of differing shelf widths. The maximum coastal tides of 2.2 m occur at Savannah, where the shelf is widest, and decrease to 1.3 m at Cape Fear and 1.1 m at Cape Canaveral. Tidal range in the Florida Keys and Florida Bay is less than 1 m.

Small frontal eddies and meanders propagate northward along the western edge of the Gulf Stream every 1-2 weeks, providing small-scale upwelling of nutrients along the shelf break of the SAB. In two areas of the SAB upwelling of nutrient-rich deep water is more permanent. The one just north of Cape Canaveral is caused by diverging isobaths; the other, which is larger and stronger, and which occurs between 32° and 33° N and results from a deflection of the Gulf Stream offshore by the topographic irregularity known as the Charleston Bump. The consistent upwelling of nutrient-rich deep waters in this region is the main steady source of nutrients near the shelf break within the entire SAB, and contributes significantly to primary and secondary production in the region. Eddy formation along the inshore edge of the Gulf Stream results in retention of eggs and larvae and their transport onshore. In the Straits of Florida, a westward-flowing countercurrent inshore of the Florida Current, and cyclonic gyres spun off the Florida current (such as the Pourtalès Gyre associated with the Pourtalès Terrace), similarly retain pelagic larvae in the area of the Florida Keys.

The width of the continental shelf varies from just a few kilometers off West Palm Beach south through the Florida Keys, to 200 km wide off Brunswick and Savannah, Georgia. The gently sloping shelf (about 1m/km) in the SAB can be divided into the following zones:

- Inner shelf (0-20 m), dominated by tidal currents, river runoff, local wind forcing, and seasonal temperature changes;
- Middle shelf (21-40 m), where waters are dominated by winds but influenced by the Gulf Stream. Stratification of the water column changes seasonally, with mixed conditions from October through March and vertical stratification from June through September. Strong stratification allows upwelled waters (caused by the effect of bottom topography on the Gulf Stream) to advect farther onshore near the bottom and, at the same time facilitates offshore spreading of lower-salinity water in the surface layer; and
- Outer shelf (41-75 m), dominated by the Gulf Stream. The shelf break generally occurs at about 50 m depth, but is shallower southward off Florida and deeper off North Carolina.

Temperature and salinity of shelf waters fluctuate seasonally (from 10 to 29°C and from 33.0 to 36.5 psu), whereas warm and salty surface Gulf Stream waters are much less variable. Off of South Florida through the ocean side of the Florida Keys, the shelf is narrow, capped by coral reefs out to the shelf edge, and under the influence of the warm tropical Florida Current.

The continental shelf of the SAB consists of sand/shell bottom, interspersed with rocky outcrops, which are particularly prominent along the shelf break in depths from 45 to 60 m. Sand- and mud-bottom areas of the continental shelf and slope support less biomass and lower diversity of species than hard-bottom areas, but sustain a few important fishery species, such as tilefish (*Lopholatilus chamaeleonticeps*), flounders (*Paralichthys* spp.), sciaenids (drums and croakers), and calico scallop (*Argopecten gibbus*). Coastal sand/mud bottom is an important habitat for penaeid shrimp, and a seasonal bottom-trawl fishery occurs along much of the coast of the region.

Hard-bottom areas of the continental shelf throughout the SAB support a warm-temperate or tropical fauna, owing to structurally complex rocky reef formations and the proximity of warm Gulf Stream waters. The rocky outcrops from North Carolina south to Cape Canaveral and the ridges off South Florida function as reefs and provide substrate for a great diversity and biomass of sessile invertebrates and algae. Diverse assemblages of polychaetes, mollusks, crustaceans, echinoderms, and other invertebrates inhabit the attached sponges, corals, and ascidians and shelter in the complex rocky bottom. Reef fish assemblages of economically valuable snappers (Lutjanidae), groupers (Serranidae), grunts (Haemulidae), porgies (Sparidae), and diverse tropical families such as wrasses (Labridae), damselfishes (Pomacentridae), and others are associated with the complex hard bottom. The areal extent and distribution of productive live bottom habitat on the continental shelf north of Cape Canaveral have not been completely mapped: estimates of extent range from 4 to 30% of the shelf area [68].

South of Cape Canaveral, a ridge system parallel to the shoreline of Florida exists along drowned coral reef tracts; these coral reefs and worm-tube and coquina-shell reefs in shallow water harbor many reef species. South of Miami, the Florida Keys contain the only system of shallow reef-building corals in the continental U.S. Although lower in coral diversity than the nearby Caribbean Sea, these reefs contain a high diversity of other invertebrates and fishes. Whereas the shelf break in the SAB occurs at about 50 m depth, in the Keys it occurs in depths from 10 to 20 m. The Florida reef tract extends in a curve of about 370 km and encompasses 6,000 patch reefs and well-developed spur-and-groove and shelf-edge coral ridge formations.

Saltmarshes and estuaries, which are particularly well developed along the southeast coast [70], include 24,000 km^2^ of coastal wetlands, including 3,600 km^2^ of salt and brackish marsh. The region from Cape Hatteras, North Carolina, through the Dry Tortugas, Florida, includes about 1,700 km of coast interrupted by hundreds of rivers, sounds, estuaries, and inlets that provide a wide diversity of habitats. About 1,000 km of this encompasses the SAB embayment between Cape Hatteras and Cape Canaveral that includes a band (up to 12 km wide) of salt marsh and tidal creeks that serve as important nursery habitat and are particularly well developed off South Carolina and Georgia. The Indian River Lagoon, Biscayne Bay, and Florida Bay estuaries are fringed by mangroves and include mangrove islets, with associated attached epifauna and motile invertebrates and fishes. The Indian River Lagoon system is particularly high in diversity because of tropical influences. These estuaries are important nursery areas for many marine fishes that spend their adult lives offshore.

The continental slope off the southeastern U.S. is unusual in that a large part of it is interrupted by the relatively flat Blake Plateau that separates the inshore Florida-Hatteras slope (200-500 m) and the offshore Blake Escarpment (1,000-3,000 m). The fauna of the slope and Blake Plateau is poorly known because of the difficulty of sampling deep, hard rocky bottoms under the swift Gulf Stream current. The hard bottoms of the Blake Plateau are colonized by a wide variety of deep-sea sponges and corals, and in some places the corals have formed significant mound and ridge systems (up to 150 m tall) with associated sponges, other cnidarians, mollusks, polychaetes, crustaceans, echinoderms, and fishes (see review in [71]).

#### Regional history of biodiversity studies

Surveys and collections of marine organisms of southeastern North America began during the U.S. colonial period (seventeenth and eighteenth centuries), and early work included Catesby's (1731-43) publication of *The Natural History of Carolina, Florida, and the Bahama Islands* [72] (see Pietsch and Anderson [73] for reviews of additional early collections of marine vertebrates). These two volumes contained descriptions and a checklist of many marine crustaceans, fishes, turtles, birds, and mammals. Many collections were sent to Europe for study during the American colonial period, and checklists of marine species were compiled as studies of the specimens were published. Of particular note were the collections of Alexander Garden of Charleston, South Carolina, who collected what were used as type specimens for original descriptions of regionally emblematic species such as striped mullet (*Mugil cephalus*), mummichog (*Fundulus heteroclitus*), black sea bass (*Centropristis striata*), and bluefish (*Pomatomus saltatrix*), among many others. William Bartram, in his *Travels Through North and South Carolina, Georgia, East and West Florida*… and John Edwards Holbrook, in several publications, provided descriptions and checklists of additional coastal marine fishes from South Carolina, Georgia, and Florida [73].

Early exploratory studies offshore along the Atlantic coast of the southeastern U.S. concentrated on finding exploitable fish populations. From 1877 to 1880, the U.S. Coast and Geodetic Survey (a predecessor agency of the current National Oceanic and Atmospheric Administration, NOAA) conducted exploratory surveys aboard the steamer *Blake* along the Atlantic coast to Florida, the Gulf of Mexico, and the Caribbean, in addition to a cruise northward into the Gulf of Maine [74]. These surveys were aimed primarily at bottom-living organisms. Agassiz [74] described habitats (including important hard-bottom and sponge-coral habitats of the southeastern shelf), oceanographic features (including the Blake Plateau, other bottom features, and the Gulf Stream), and organisms (including many new genera and species) of the continental shelf and deep sea of the region, and noted similarities and differences between the fauna and that of the Caribbean to the south. The Gulf Stream was noted as a major influence on faunal composition on the outer continental shelf; cold-temperate benthic species were found inshore and offshore of the Gulf Stream on bottoms of a variety of types. These early descriptions of deep corals collected on the Blake Plateau, along with recent concern for damage to fragile deep-coral habitats by fishing, have led to recent exploration of deep coral banks and other deep habitats in the SAB-Florida East Coast area (see NOAA Ocean Exploration, below). Descriptions of oceanic fishes from early expeditions (including those of the *Blake*) were summarized in 1896 by Goode and Bean [75].

Since the mid-twentieth century, NOAA and its predecessor agencies (e.g., the Bureau of Commercial Fisheries) have explored habitats and their natural resources off the coast of the southeastern U.S. Beginning in the 1950s, ships such as *Silver Bay*, *Pelican*, *Oregon*, and *Gill* conducted exploratory fishing surveys using trawl nets. These early surveys found concentrations of snappers, groupers, and other economically valuable fishes associated with rocky outcrops and other hard-bottom reefs on the continental shelf and shelf-edge. They also documented the significant fishery resources (drums, flatfishes, mullets, herrings, shrimps) of soft-bottom communities. Many taxonomic (e.g., [76]) and a few ecological papers and monographs (e.g., [77]) resulted from these early fishery surveys. Fishery surveys conducted or funded by NOAA, which continue, have included bottom-trawl and plankton surveys that have catalogued biodiversity. Additional surveys using dredges, grabs, and other benthic samplers have collected invertebrates and new species. The most valuable surveys of fish diversity, distribution, and abundance on the continental shelf have been conducted by the NOAA MARMAP (Marine Resources Monitoring, Assessment and Prediction) and SEAMAP (Southeast Area Monitoring and Assessment Program) monitoring programs (e.g., [78], [79]). Significant regional invertebrate surveys of the SAB were conducted under the auspices of the Bureau of Land Management (BLM) and Minerals Management Service (MMS), as baseline environmental studies before anticipated petroleum exploration and production in the region (e.g., [80]). Data from the long-term MARMAP and SEAMAP surveys, along with life history and ecological studies on many of the species collected, have been summarized in several publications and have been used extensively for fisheries stock assessments and for planning of marine protected (MPAs)areas in the SAB [81], [82]. The data have also been included in OBIS-USA and are a significant contribution from the Southeast U.S. to international OBIS.

From the last quarter of the twentieth century to the present, significant contributions to cataloging the biodiversity of the continental shelf and slope off North Carolina and in the tropical western North Atlantic were made by the Duke University Marine Laboratory (DUML) (e.g., [83]) and the Rosenstiel School of Marine and Atmospheric Sciences (RSMAS) of the University of Miami, respectively. Explorations by DUML used the RV *Eastward* and the RV *Cape Hatteras*. RSMAS collections and archives (Marine Invertebrate Museum: http://rsmas.miami.edu/divs/mbf/invert-museum.html) document the biodiversity of Atlantic and Gulf of Mexico tropical and deep-sea species and include material from the Straits of Florida and the Florida Keys National Marine Sanctuary. Marine resource agencies of the states have also conducted faunal and fishery surveys within state waters, particularly within estuaries.

Many of these surveys defined the distribution, life history, abundance, and biodiversity of species of historical, social, or economical importance, and resulted in checklists, identification guides, and life history reviews of ecologically dominant or economically valuable taxa. Many monographs documented diversity within taxa, which included seaweeds, sea anemones, mollusks, crustaceans, echinoderms, sharks, bony fishes, turtles, birds, and whales (e.g., [79], [84], [85], [86], [87], [88]). An important review, checklist, and identification guide for fishes and many economically valuable invertebrates is included in Carpenter [69], which lists 987 fishes for the western Central Atlantic, most of which would be expected to be found in the SAB-Florida East Coast. Hare et al. [89] reported 181 species of fish at Gray's Reef National Marine Sanctuary off Georgia. The Florida Keys ecosystem supports over 6,000 species of plants (367 algae, 5 seagrass), fishes (520), and invertebrates (including 65 stony corals) in the nation's only continental barrier coral reef and the largest contiguous seagrass community in the western hemisphere [90]. Eggs or larvae of at least 70 families of fishes have been collected in ichthyoplankton surveys in the SAB [91]. Diversity of benthic invertebrates from six hard-bottom areas off South Carolina and Georgia sampled with dredge, trawl, and suction/grab samplers yielded 432, 525, and 845 unique taxa (most taxa identified to species, some only to genus or family), respectively, for the three gear types [92]. The Bryozoa (91 taxa), Porifera (89 taxa), and Cnidaria (70 taxa) dominated dredge collections from all seasons in terms of numbers of taxa. Porifera (111 taxa) were also well represented in trawl collections, along with other taxa such as decapod Crustacea (86 taxa), Bryozoa (85 taxa), and Mollusca (85 taxa). The work of Wenner et al. [92] showed that Porifera, Bryozoa, and Cnidaria are the most diverse taxonomic groups of benthic invertebrates encountered on the continental shelf and shelf break off of South Carolina, Georgia, and northern Florida.

Blake and Grassle [93] found that the diversity of benthic slope fauna was much higher off the Carolinas than at similar depths in the Mid-Atlantic Bight. A station at 800 m on the slope east of Charleston produced the highest diversity value ever recorded for the marine environment and supports the view that the region is probably an important reservoir for marine biodiversity [93].

Surveys of estuarine habitats have revealed moderate diversity levels of certain taxa. Ross and Bichy [94] reported 155 and 103 fish species, representing 58 families, from Masonboro Island and Zeke's Island components, respectively, of the North Carolina National Estuarine Research Reserve (NERR). Surveys of the Ashepoo, Combahee, and Edisto (ACE) Rivers in South Carolina (within the boundaries of the ACE Basin NERR) collected 79 species of fishes and 26 species of decapod crustaceans in bottom trawls [95].

#### The known, unknown, and future directions

The areas of the most intense study of species assemblages and biodiversity are generally those of interest to fisheries, petroleum or mineral extraction, or ocean dumping, where faunal surveys and environmental impact assessments have been done. Fishery habitats of interest include estuarine oyster reefs and hard-bottom or coral reefs of the continental shelf and shelf edge. Faunal surveys for environmental assessment of benthic invertebrates and fishes have been done for nearshore dredge and dredged-material-disposal sites. Additional intense studies have been done in and around the Gray's Reef and Florida Keys National Marine Sanctuaries and NERRs.

Areas of well-known biodiversity are the coral reefs of the Florida Keys (e.g., 520 fish species), estuarine oyster reefs (36 fish species), and hard-bottom areas of the continental shelf and slope (181 fish species at Gray's Reef National Marine Sanctuary); these are also areas of high biodiversity [89], [90], [96]. Diversity measurements in fish assemblages of hard-bottom reefs are higher than those noted in similar studies off the middle Atlantic states of the U.S., but not as high as those in the Florida Keys and Caribbean. Within the SAB, fish diversity increases with decreasing latitude on the continental shelf [97]. Within the entire SAB-Florida East Coast, the area of highest species richness is waters surrounding southern Florida, with diversity decreasing northward on either coast of the peninsula. The Straits of Florida are likely the most species-rich area for fishes in the Atlantic [69]. The pattern for species richness in fishes is repeated for levels of endemism, with the greatest concentration of endemic fishes centered on the Straits of Florida [69].

Within the region, several species are of historical, social, or economic importance, or are emblematic of the region and the challenges faced in conservation of biodiversity. Some threatened and endangered species such as loggerhead sea turtle (*Caretta caretta*) and eastern brown pelican (*Pelecanus occidentalis*) have been successfully saved from extinction, yet threats remain for them, as well as for Atlantic bluefin tuna (*Thunnus thynnus*), sturgeons (*Ascipenser* spp.), Atlantic right whales (*Eubalaena glacialis*), and *Acropora* corals. The poor condition of corals of the Florida Keys over the last three decades results from a combination of many factors, including effects of human population through coastal development, overfishing, ship groundings, and water quality degradation from terrestrial, marine, and atmospheric pollution (including temperature increases). In a notice published on 12 February 2010, NMFS announced it will evaluate the status of 82 species of stony coral that the Center for Biological Diversity has asked to be listed as threatened or endangered under the Endangered Species Act. These include *Montastrea* spp., which form large colonies and are important in building reefs of the Florida Keys, and *Oculina varicosa*, which occurs on deep reefs in the region.

Within the pelagic realm, the pelagic brown algae *Sargassum natans* and *S. fluitans* (gulfweed) form a complex but dynamic habitat used by a diversity of cnidarians, bryozoans, crustaceans, polychaetes, and other invertebrates, as well as juveniles and adults of many fishes [98].

Deeper faunas of the continental slope and abyss are less well known than those of shelf areas, but were described in early explorations, and by Menzies et al. [83]. In recent years, the NOAA Office of Ocean Exploration has funded collections and submersible observations of deep-reef and other habitats of the SAB, and additional checklists of species and descriptions of their habitats are emerging. Such studies on deep corals and sponges have revealed a high diversity of polychaetes, mollusks, and crustaceans associated with large sessile epifauna [99]. This is an area for additional exploration. The biodiversity of inquiline species is apparently high, but only a few individuals of a few species of sponge, coral, and ascidian have been examined for associated endofauna. A range of hosts, especially in deep water, is unexamined and may contain a great diversity of endofauna. Poorly known geographic areas include the complex hard bottom of the Blake Plateau and Charleston Bump, including deep coral habitats. Recent submersible explorations have resulted in collection of previously poorly known or rare species, which have been shown with further exploration to be quite common (e.g., [100], [101]). Planktonic communities and microbes of benthic and pelagic habitats are poorly known. All “bioengineered” habitats such as coral banks, worm-tube mounds and reefs, sponge reefs, tilefish burrows, red grouper (*Epinephelus morio*) excavations, and similar structures are special habitats that are poorly studied and have interesting and complex symbiotic relationships.

Areas of relatively low biodiversity include lower reaches of estuaries, anoxic areas, and sand/mud bottom of the shelf. Anoxic dead zones do not naturally occur in the region, but the deep reaches of some dredged estuaries are anoxic. Most sampling has been directed at structurally complex hard bottoms because of their importance in fisheries and as potential areas of petroleum deposits, so low-diversity habitats such as sand and mud bottom may need additional study to determine the factors that affect biodiversity in the region.

Assemblages of fishes support important fisheries. Because of the diversity of species and life history strategies, and the nonselective nature of some fishing gear, traditional management by imposing limits on individual species is difficult. In addition, the complex ecological relationships among targeted species and their predators, prey, and habitats are poorly understood, thus delaying the development of ecosystem-based management. Additional study of assemblages, and of the interrelationships of the species in those communities, is needed.

Much of the taxonomic literature for the region is scattered in descriptions of single species and in monographs on higher taxa, including families, orders, and classes. Much taxonomic and ecological research deals with more than one LME, making it difficult to assess the biodiversity of the region. Compiling regional checklists and identification guides is needed to provide baseline data on biodiversity of the region. Current knowledge of the biota of the Southeast U.S. Continental Shelf LME is summarized in Table 5; more detail is provided in Table S1 and Table S5.

**Table 5:** Biotic diversity in the Southeast U.S. Continental Shelf Large Marine Ecosystem.

Taxonomic group	No. species^1,2^
**Domain Archaea**	**UD**
**Domain Bacteria (including Cyanobacteria)**	**48 (16)**
**Domain Eukarya**	**4,229**
**Kingdom Chromista**	**217**
Phaeophyta	217
**Kingdom Plantae**	**113**
Chlorophyta	65
Rhodophyta	38
Angiospermae	10
**Kingdom Protoctista (Protozoa)**	**165**
Dinomastigota (Dinoflagellata)	
Foraminifera	165
**Kingdom Animalia**	**3,734**
Porifera	111
Cnidaria	362
Platyhelminthes	
Mollusca	698
Annelida	400
Crustacea	696
Bryozoa	91
Echinodermata	
Urochordata (Tunicata)	35
Other invertebrates	41
Vertebrata (Pisces)	1200
Other vertebrates	100
**TOTAL REGIONAL DIVERSITY** 3	**4,277**

**Notes:**

1 Sources of the reports: databases, scientific literature, books, field guides, technical reports, and personal communication with taxonomic experts.

2 Identification guides cited in Text S2.

3 Includes all taxonomic groups as reported in Tables S1 and S5.

UD  =  Listed in work but number undetermined to date.

#### Trouble spots and emerging issues

In addition to general degradation of water quality, increasing sea temperature, and ocean acidification, many habitats in the region are threatened by overfishing and coastal development. Overfishing has severely depleted populations of top-level demersal predatory fishes such as sharks, snappers, groupers, and jacks (Carangidae). Populations of pelagic sharks, tunas, and mackerels (Scombridae) are also currently or periodically overfished. Fishing pressure and demand remain high, and management is often slow to respond. Management efforts are aimed at restoring sustainable stocks of individual species, and little effort has historically been made to restore sustainable functioning ecosystems.

Efforts at restoring some endangered and threatened species have been successful, as in the cases of the eastern brown pelican and loggerhead sea turtle. However, marine mammals, such as Atlantic right whale and the Florida manatee, are still endangered. Reef-forming corals of the Florida Keys are declining [90], and decades of fishing on aggregations of spawning reef fishes have resulted in declining abundance in aggregations. Recent protection of spawning sites has reversed this trend for mutton snapper (*Lutjanus analis*) [102], and may be effective for other species.

Efforts at designating reef areas as no-fishing zones have been successful in restoring populations of top-level predatory fishes in the Florida Keys [103], and recent implementation of small areas where bottom fishing is not allowed in the SAB show promise of restoring predators in those areas as well. The small MPAs in the SAB will be useful in providing data on how no-take zones established for the conservation of habitat and restoration of fishery species affect sustainable fisheries and biodiversity. Unfortunately, those areas now have large populations of the invasive lionfish (*Pterois* spp.), first discovered in the MPAs in 2002, before designation. Because lionfish have no predators in this system and they prey on small fishes, including new recruits, their impact on endemic fish population recovery and restoration within the new MPAs is a concern.

The fisheries operating in the Western Central Atlantic land the greatest diversity of fishes of any Atlantic region [69], and nonselective fishing gear, invasive species, environmental factors beyond the control of the South Atlantic Fishery Management Council (SAFMC) (e.g., global change), along with the high biodiversity, will continue to make management for sustainable fisheries and conservation of biodiversity a difficult task.

Several MPAs exist in the region. In addition to three National Marine Sanctuaries (*Monitor*, Gray's Reef, and Florida Keys), the SAFMC established special zones to protect banks of ivory tree coral (*Oculina varicosa*) on the upper slope off Florida [71] (Figure 5). In 1984, the SAFMC designated the Oculina Bank as a Habitat Area of Particular Concern (HAPC). This action closed an area of 92 km^2^ to trawling, dredging, longlining, and trapping, and established other restrictions. In 1994, the SAFMC created the Experimental Oculina Research Reserve, closing the area to all bottom fishing indefinitely. These restrictions were put into place to protect spawning reef fishes, restore reef fish stocks, and protect particularly sensitive habitats or species assemblages of the coral and associated organisms that include at least 350 invertebrate species [71], [104]. An additional large HAPC has been approved by the SAFMC to protect deep (more than 400 m) banks of the coral *Lophelia* and other coral banks on the Blake Plateau and the Straits of Florida. These efforts are expected to have positive impacts on the conservation of biodiversity.

In February 2009, the SAFMC established eight no-bottom-fishing zones on the outer continental shelf between southern North Carolina and the Florida Keys ranging in area from 27 to 514 km^2^. These small areas are aimed at protecting deepwater reef species and providing areas where a natural reef ecosystem can function.

The region contains several NERRs. These include the North Carolina NERR (which encompasses four sites from Currituck Banks south to Masonboro Island); North Inlet-Winyah Bay NERR and ACE Basin NERR, South Carolina; Sapelo Island NERR, Georgia; and Guana Tolomato Matanzas NERR, northeast Florida. These areas comprise large shallow sounds and other estuarine lagoons and tidal creeks, relatively pristine saltmarsh, mangrove and other wetlands, subtidal seagrass and oyster beds, and upland maritime forest.

The Charleston Bump has seasonal fisheries closures, pelagic longline in February-April and bottom wreckfish (*Polyprion americanus*) in January-April. Thus, for much of the period from mid-January to mid-April, little or no fishing occurs on the Charleston Bump. The Charleston Bump is included in the deep-sea coral HAPC under consideration by the SAFMC.

In spite of recent measures to protect offshore habitats, coastal development continues to have an impact on habitats of estuarine species and estuarine-dependent stages of shelf species. Coral reefs are in decline worldwide, and the Florida Keys are no exception. Global change and concomitant ocean acidification and sea level rise will continue to affect these nearshore habitats. The region lies at the crossroads between tropical and temperate faunas and would be a good area for monitoring effects of climate change on biodiversity and changing faunas.

Fisheries in the region target a diverse assemblage of reef fishes that exist in physically stable environments characterized by biologically accommodated communities. Overfishing of individual species and fishing gear effects are likely to have an impact on the health of populations of associated algae, invertebrates, and other vertebrates; many of the behavioral and trophic interactions among species in these diverse assemblages are poorly understood.

New species and assemblages are likely to be discovered in deep sponge/coral endofauna, and in the complex hard bottom that underlies swift Gulf Stream currents on the Blake Plateau.

#### The Census of Marine Life contribution to the Atlantic Bight and Florida East Coast region

The Census, through OBIS, is currently summarizing and mapping decades of fishery survey data that have not traditionally been used for the valuable biodiversity data that such surveys contain. While some previous mapping of fishery species has been done (e.g., http://ocean.floridamarine.org/efh_coral/ims/viewer.htm), extensive datasets on distribution, abundance, and size of several hundred additional, nonfishery species are available for mapping.

### Gulf of Mexico Large Marine Ecosystem

#### Description of the Gulf of Mexico region

**Figure 6:**
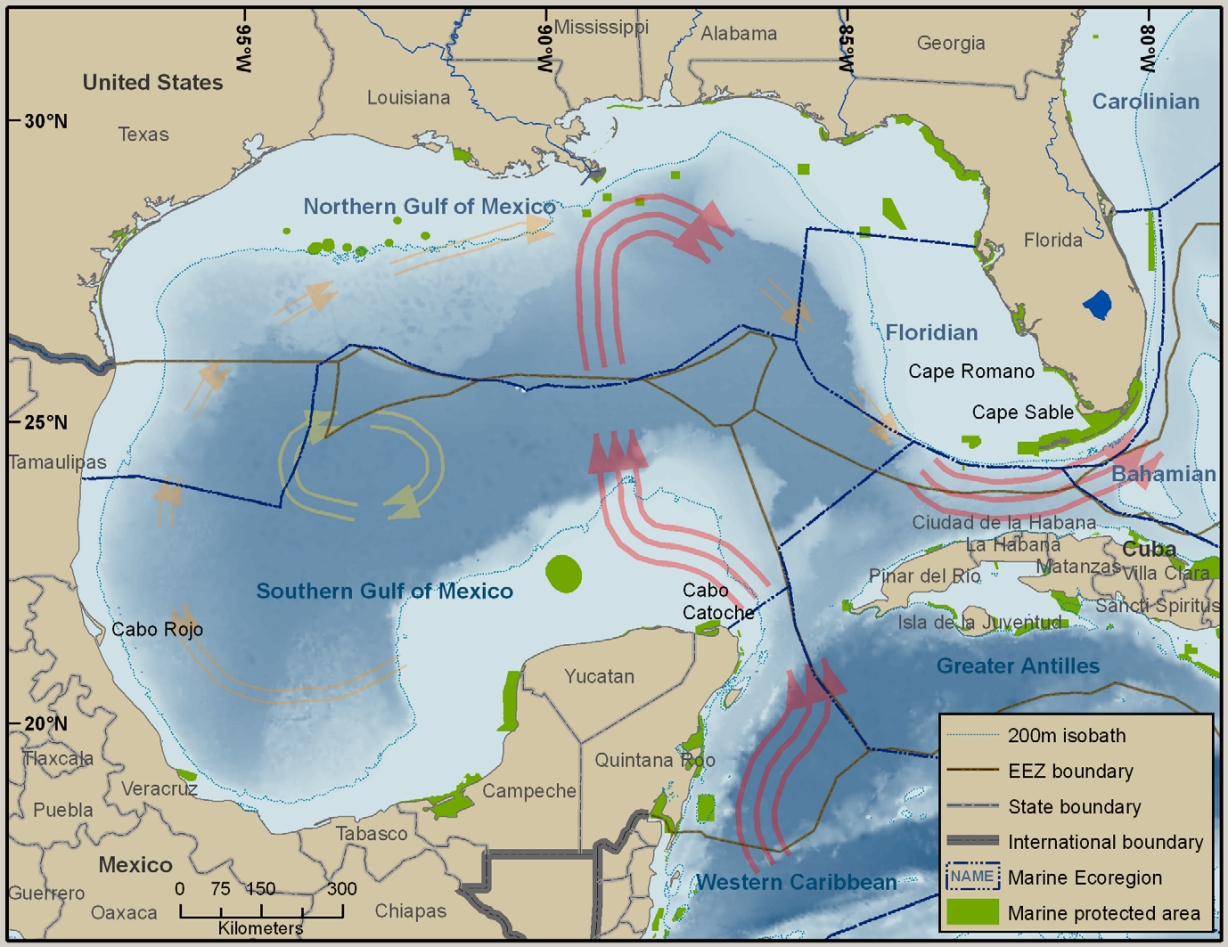
Gulf of Mexico Large Marine Ecosystem, surrounded by United States, Mexico, and Cuba. Map also shows EEZ boundaries, state boundaries, international boundaries, marine ecoregions, and marine protected areas. The large pink arrows in the eastern Gulf represent the dominant Loop Current.

The Gulf of Mexico LME, located in the southeastern part of North America, is surrounded by the U.S., Mexico, and Cuba, and encompasses three ecoregions, the northern Gulf, southern Gulf, and Floridian [105]. Occupying a surface area of more than 1.5 million km^2^, its maximum east-west dimension is 1,573 km and it is 900 km from north to south between the Mississippi Delta and Yucatan Peninsula. The shoreline, extending from Cape Sable, Florida, to Cabo Catoche, Quintana Roo, Mexico, is about 5,696 km long; it includes another 380 km of Gulf shoreline in Cuba from Cabo San Antonio in the west to Havana in the east [106] (Figure 6).

The Gulf of Mexico basin resembles a bowl with a shallow rim. The shallow continental shelves are narrow and terrigenous in the west, moderately broad and terrigenous in the north, and wide, carbonate platforms in the east, adjacent to the Florida and Yucatan peninsulas. On an area basis, roughly 32% of the Gulf is continental shelf, 41% is continual slope (200-3,000 m), and 24% is abyssal plain (more than 3,000 m). The deepest area (more than 3,800 m) occurs within the Sigsbee Deep [106], [107].

Warm, tropical water enters the Gulf of Mexico from the Caribbean Sea between the Yucatan Peninsula and Cuba via the Yucatan Straits, where it forms the main Gulf current, the Loop Current. Large eddies occasionally spin off of this major current and move westward [108]. The Loop Current exits via the Florida Straits between Florida and Cuba and forms one of the world's strongest and most important currents, the Gulf Stream.

As a large receiving basin, the Gulf of Mexico receives extensive drainages from five countries (U.S., Canada, Mexico, Guatemala, and Cuba), including over two-thirds of the U.S. watershed. The Mississippi River dominates drainage in the north and the Grijava-Usumacinta River System dominates in the south. Thirty-three major river outlets and 207 estuaries and lagoons are found along the Gulf coastline [105], [106].

Biologically, the shallow waters of the northern Gulf are warm-temperate (Carolinian Province) and those in the south are tropical (Caribbean Province) [109]. Oyster reefs and salt marshes are the dominant habitat type in northern, low-salinity estuaries, and seagrass beds are common in clearer, more saline bays. In the tropical southern Gulf, mangroves line bay and lagoon shorelines with oyster reefs, salt marshes, and seagrasses distributed in similar salinity conditions as the northern Gulf. In the western Gulf, wedged between two wet regions, the Laguna Madre of Texas and Tamaulipas exist as the most famous of only five hypersaline lagoons in the world [110], where salinity historically ranged over 100. After dredging of the Gulf Intracoastal Waterway in Texas (late 1940s) and barrier island passes and inlets in Texas and Mexico, salinities have moderated and fluctuate around 40. This highly productive lagoon has extensive wind-tidal flats and shallow seagrass beds in a semiarid region. Offshore, coral reefs are common in the Florida Keys, Cuba, and the southern Gulf off the state of Veracruz and on the Campeche Bank [111], and other topographic highs or hard bottoms are sporadic on the normally smooth, soft substratum of the continental shelves [112], [113]. Unique, recently discovered, and highly diverse habitats in deeper Gulf waters include chemosynthetic communities and communities of deepwater corals (*Lophelia* reefs) [114], [115], [116].

#### Regional history of biodiversity studies

The history of research on coastal and marine biota of the Gulf of Mexico can be divided into three periods: early exploration, local coastal studies, and large-scale, multidisciplinary investigations and synthesis [107]. The exploratory period (1850-1939) included collecting on field expeditions and exploratory cruises by federal agencies and museums, primarily from the northeastern U.S. The local coastal period (1940-59) saw establishment of federal, state, and academic laboratories in coastal locations around the Gulf. Prominent locations included (clockwise): Port Aransas and Galveston, Texas; Gran Isle, Louisiana; Ocean Springs and Pascagoula, Mississippi; Alligator Harbor and St. Petersburg, Florida; and Havana, Cuba. Knowledge of the biota around these laboratories increased greatly during this period, and one particularly important publication [117] summarized existing knowledge of the origin, waters, and marine life of the Gulf. In addition to a wide range of topics covered, it represented the first compilation of species from numerous taxa living in the Gulf (2,444 species). Starting in 1960, multidisciplinary investigation and syntheses resulted in an increased number of coastal laboratories and the sponsorship of large state and federal investigations on the continental shelves. Some examples are the NMFS studies (1961-65); Florida *Hourglass Cruises* (1965-67); BLM studies off Mississippi, Alabama, and Florida and the South Texas Outer Continental Shelf in the 1970s; BLM and MMS Topographic Features Program (1975-85); NMFS and EPA Buccaneer Gas and Oil Field Program (1977-80); and Department of Energy Strategic Petroleum Reserve Studies (1981-85). As oil and gas exploration and production moved deeper, MMS began funding studies on the continental slope and deep Gulf of Mexico. In addition to the numerous natural bottom ecosystem studies, MMS also funded studies of oil and gas platforms in the northwestern Gulf, most of which are available on the MMS Gulf of Mexico Region Web site (http://www.gomr.mms.gov/). This “steel archipelago” provides hard-substratum habitat from shallow to deep areas on thousands of platforms (over 4,000 in 2005).

Large ecosystem studies were also conducted in Mexico by scientists from the Universidad Nacional Autonoma de Mexico, Instituto de Ciencias del Mar y Limnologia. The two major studies in the 1990s were OGMEX (Oceanografia del Golfo de Mexico) and COBEMEX (Communidades Bentonicas del Golfo de Mexico) [118].

Several iconic or well-known species are of historical, social, and economic importance [119]. The West Indian monk seal (*Monachus tropicalis*) was probably the first large animal to go extinct because of human activity in the Gulf and Caribbean. It was last seen on the Campeche Bank islands in 1948 and in the Caribbean in the early 1950s [120]. Other Gulf of Mexico species that became endangered include the Kemp's Ridley sea turtle (*Lepidochelys kempii*) and whooping crane (*Grus americana*). Restoration programs for each of these have increased their population numbers in recent decades. West Indian manatees (*Trichechus manatus*) are greatly reduced and now exist only in certain drainages along the west coast of Florida. Menhaden (*Brevoortia patronus*) is the largest commercial fishery by weight, and the penaeid shrimp fishery is the largest by value (white shrimp *Litopenaeus setiferus*, pink shrimp *Farfantepenaeus duorarum*, and brown shrimp *Farfantepenaeus aztecus*). Various grouper and snapper species are predominant commercial species offshore in Cuba and Mexico, as well as in the Florida Keys and on scattered hard-bottom areas off Florida (grouper) and Texas (snapper).

Predominant commercial coastal shellfish in the northern Gulf include the American oyster (*Crassostrea virginica*) and the blue crab (*Callinectes sapidus*) [121], [122]. In the tropical southern Gulf, the spiny lobster (*Panulirus argus*) and the queen or pink conch (*Eustrombus gigas*) are taken. However, these are commercially extinct in many areas now and are taken only by recreational fishers, sometimes under strict regulations [123].

The two species of largest recreational catch and economic value in the northern Gulf are spotted sea trout (*Cynoscion nebulosus*) and red drum (*Scianops ocellatus*) [121], [122]. The red snapper (*Lutjanus campecheanus*) is a favorite offshore recreational species in the northwestern Gulf and one that has recently occasioned fishery management discussions and regulation. The bottlenose dolphin (*Tursiops truncatus*), probably the single most recognizable Gulf species by the public, is abundant in coastal bays and estuaries, as well as offshore in the northern Gulf, and is found gulfwide [120].

All of these studies increased our knowledge of the presence, abundance, and distribution of biota in the Gulf of Mexico. However, there had been no comprehensive species compilation since Galtsoff [117] until the Biodiversity of the Gulf of Mexico Project [124], which lists 15,419 species in 40 phyla of microbes, plants, and animals (Table 6; more detailed Table S1). Subsequent phases of this project will include data analysis, exploration to fill data gaps, and conversion of this benchmark work into a web-based database for OBIS and GulfBase (http://www.gulfbase.org/). The list of contributors to Biodiversity of the Gulf of Mexico Project [124] can be found in Text S3. Significant databases containing biodiversity information are listed in Table S4.

**Table 6:** Biotic diversity in the Gulf of Mexico.

Taxonomic group	No. species^1,2^
**Domain Archaea**	**UD**
**Domain Bacteria (including Cyanobacteria)**	**UD (45)**
**Domain Eukarya**	**15,374**
**Kingdom Chromista**	**1,034**
Phaeophyta	86
**Kingdom Plantae**	**967**
Chlorophyta	195
Rhodophyta	392
Angiospermae	370
**Kingdom Protoctista (Protozoa)**	**2,169**
Dinomastigota (Dinoflagellata)	644
Foraminifera	951
**Kingdom Animalia**	**11,150**
Porifera	339
Cnidaria	792
Platyhelminthes	705
Mollusca	2455
Annelida	866
Crustacea	2579
Bryozoa	266
Echinodermata	522
Urochordata (Tunicata)	102
Other invertebrates	549
Vertebrata (Pisces)	1541
Other vertebrates	434
**TOTAL REGIONAL DIVERSITY** 3	**15,419** 4

**Notes:**

1 Sources of the reports: databases, scientific literature, books, field guides, technical reports, and personal communication with taxonomic experts.

2 Identification guides cited in Text S2.

3 Includes all taxonomic groups as reported in Table S1.

4 Includes 54 species of fungi.

UD  =  Listed in work but number undetermined to date.

#### The known, unknown, and future directions

The areas of most intense study have been near state, federal, private, and academic laboratories around the Gulf coast and in areas of high economic, fishery, or ecological interest. For instance, the northwestern Gulf of Mexico, one of the most active oil and gas production areas in the world, has received funding for decades by MMS, primarily regarding environmental studies, monitoring, and protection. The Florida Keys coral reef and island ecosystem has received extensive attention, funding, and study since being designated a National Marine Sanctuary in 1990 [125], and the Flower Garden Banks National Marine Sanctuary has received similar attention since its designation in 1992 [126]. Coastal habitats, such as oyster reefs and seagrass beds, known for high biodiversity, have received considerable attention.

By contrast, much of the coastal and shelf areas in Mexico and Cuba are little known due to the lack of research funding or coastal infrastructure to support field studies. The Cuban shelf between Havana and the western tip of Cabo San Antonio, which has extensive mangroves, seagrass beds, and coral reefs, is one of the least studied areas of Cuba. Mexico's oceanographic vessel, the RV *Justo Sierra*, has greatly expanded research capability and knowledge in recent decades in the southern Gulf of Mexico. Several recent collaborative publications have broadly increased knowledge about fishery resources [127], environmental contamination and impacts [128], and the environment and its condition of the southern Gulf of Mexico [129], [130]. More remote hard-bottom areas, especially in Mexico and Cuba, have been little explored. These include the offshore area between the U.S.-Mexico border and Tampico, the nearshore and offshore volcanic rocky shores of the state of Veracruz, and the Campeche Bank continental shelf and shorelines.

Gulfwide biodiversity patterns cannot be completely explained for lack of complete information, although we know that the Gulf of Mexico exhibits great habitat complexity that probably supports high levels of biodiversity due to both endemic and cosmopolitan species [118]. Linkage to the Caribbean Sea with large-scale circulation renders the southern and eastern Gulf of Mexico with a distinct Caribbean biota. However, there appears to be strong regional endemism, as demonstrated in large-scale studies across the entire northern Gulf [118], [131], [132]. Eventual analysis of databases from the Biodiversity of the Gulf of Mexico Project will provide considerable insight into spatial distribution of species. Of 15,419 species, 1,511 (10%) are endemic to the Gulf of Mexico and 341 (2%) are nonindigenous [124]. The most diverse taxa are crustaceans (2,579 species), mollusks (2,455), and vertebrates (1,975); the least diverse are kinorhynchs (2 species), entoprocts (2), priapulids (1), hemichordates (5), and cephalochordates (5). Representatives of additional taxa known to exist in the Gulf of Mexico (placozoans, orthonectids, loriciferans, and pogonophorans) have not yet been identified.

#### Trouble spots and emerging issues

Ecosystem goods and services generated by marine biodiversity have been affected in selected areas primarily because of overfishing, habitat loss, or degradation in water quality. Harmful algal blooms [133] and hypoxia [134] regularly drive mobile animals from certain areas, and increasing coastal development encroaches upon or destroys habitats. In recent years, intense hurricanes have caused extensive coastal habitat damage and loss in the Gulf of Mexico.

The range of variability in and knowledge of the status (threatened, endangered, invasive) of species in some higher taxa in the Gulf of Mexico is wide. The larger “charismatic megafauna” (sea turtles, large sea birds, marine mammals) are far better known than microbes, sponges, and worms. Within the Gulf, all five sea turtles, all seabirds and colonial waterbirds (herons, egrets, gulls, terns, etc.), and all marine mammals have protected status at various levels. The most endangered and best-known species include the Kemp's Ridley sea turtle, the whooping crane, the piping plover, the reddish egret, and all the great whales [119], [120], [135].

Recent notable invasive species include the Australian spotted jellyfish (*Phyllorhiza punctata*) and the brown and green mussels (*Perna perna* and *P. viridis*, respectively). The spotted jellyfish bloom in the northern Gulf of Mexico was so great in 2000 that shrimp trawls clogged in minutes and fisheries were shut down [136]. Brown mussels blanketed coastal jetties and rocky shores from Corpus Christi, Texas, to Veracruz, Mexico, in the early 1990s [137], [138]. A die-off caused by El Niño increased mean summer water temperatures [139] and reduced the populations significantly after that, but the green mussel invaded Tampa Bay in 1999 and was discovered in coastal power plant intake pipes [140]. The orange cup coral (*Tubastraea coccinea*), originally from the Indo-Pacific, invaded the Gulf of Mexico in the late 1970s and is now found throughout the Gulf on oil and gas platforms, other artificial structures, and coral reefs [141].

Overfishing in the northern Gulf of Mexico has affected both commercial and recreational fisheries [142], [143]. Impacts have been addressed by regulations imposed on fisher groups, and in several cases positive results have followed. For example, when red drum populations plummeted in the 1980s, commercial harvest was stopped [144], and this species, along with the spotted sea trout, were declared game fish. Aggressive management action to ban commercial netting and sale of these species was successful, as was implementation of strict bag and size limits and hatchery-based restoration efforts were implemented with success.

In the southern Gulf of Mexico, the shrimp fishery, based primarily out of Campeche, Mexico, has almost completely ceased because of a combination of overfishing, underregulation, and environmental change [145]. Octopus is one of the most important fishery resources in the southern Gulf: *Octopus maya* represents 80% of the catch and *O. vulgaris* the rest [146]. The octopus fishery is fully exploited, and management measures need revision to maintain sustainability. Conch and lobster fisheries on coral reefs in the southern Gulf of Mexico are essentially extinct commercially because of overharvesting and lack of regulations or enforcement of regulations [123]. Red grouper (*Epinephelus morio*) is the most important fishery resource on the continental shelf of the Campeche Bank [147]. Reduced catches and sizes of grouper and snapper on coral reefs in the southern Gulf during the 1970s led to the harvesting of herbivorous fishes, which are also now reduced in numbers on many reefs [111]. Heavy shark fishing on these reefs led to reduced populations and catches around almost all reefs in the 1990s.

Historically, MPAs in the Gulf of Mexico, as in most of the rest of the world, focused on coastal areas to protect threatened species or unique habitats, like whooping cranes at the Aransas National Wildlife Refuge in coastal Texas or the coral reefs of John Pennecamp Coral Reef State Park in the Florida Keys. During the 1990s, offshore sites were added with the beginning of the National Marine Sanctuary Program (NMSP) (Florida Keys National Marine Sanctuary and Flower Gardens Banks National Marine Sanctuary) in the U.S. and the National Commission of Protected Natural Areas (Veracruz Reefs National Park and Alacran Reef National Park) in Mexico (see Figure 6). Recently, a network of MPAs has been suggested and considered by both the U.S. and Mexico. This concept, known as the “Islands in the Stream,” would involve a system or network of MPAs around the Gulf of Mexico, primarily on offshore hard-bottom reefs and banks. Ocean circulation would link populations at network sites [148].

Habitat loss and destruction, and degradation of water quality in the Gulf are two of the key issues addressed in the Governors' Action Plan for Healthy and Resilient Coasts [149]. Although habitat loss is greatest in Louisiana, where 80-130 km^2^ of coastal wetlands are lost each year, other states are experiencing significant loss due to coastal development and infrastructure in selected areas. The Gulf of Mexico Alliance is dedicated to working within and across states to slow this loss, as well as encourage and support habitat restoration.

Global change has been the subject of several major multidisciplinary studies involving numerous state and federal agencies, private industry, and academia. Informative and educational reports have been widely distributed in the northern Gulf area as an alert to environmental change and its impact on habitat, biodiversity, and quality of life [150], [151], [152]. In Texas and Louisiana, sea level rise has been compounded by the subsidence of some coastal lands due to water and petroleum extraction [153].

Overexploitation of shell resources by individuals and for commercial sales, principally in the tourism industry, has resulted in restrictions in some places (e.g., Sanibel Island, Florida, and South Padre Island, Texas), but sales of shells, soft and hard corals, and hawks bill sea turtle carapaces continue in street markets of Veracruz, Mexico.

The best opportunities for potential future discoveries of new species and communities are likely in the remote, unexplored areas of the Mexican coast (northeast Mexico and Campeche Bank), the northwest coast of Cuba, and deepwater areas of the lower continental slope and abyss in all areas. Small, soft-bodied and shell-less invertebrates are often undersampled; as better sampling protocols are developed, many new discoveries will be made in the Gulf of Mexico and elsewhere.

#### The Census of Marine Life contribution to the Gulf of Mexico region

Although the Gulf of Mexico Biodiversity Project is an affiliate project within Census, it was well under way before joining the Census. Its final product (a complete all-taxa inventory) is unique among Census projects and will be one of only a few such inventories ever completed for a LME. Two other Census projects had study sites in the Gulf of Mexico. NaGISA had sites in the northeastern Gulf at Destin, Florida, and in the southeastern Gulf at La Habana, Cuba. COMARGE also had two sites, on muddy slopes, on the Mississippi Canyon in the northern Gulf of Mexico and at chemosynthetic ecosystems in the southern Gulf.

### Insular Pacific–Hawaiian Large Marine Ecosystem

#### Description of the Insular Pacific-Hawaiian region

**Figure 7:**
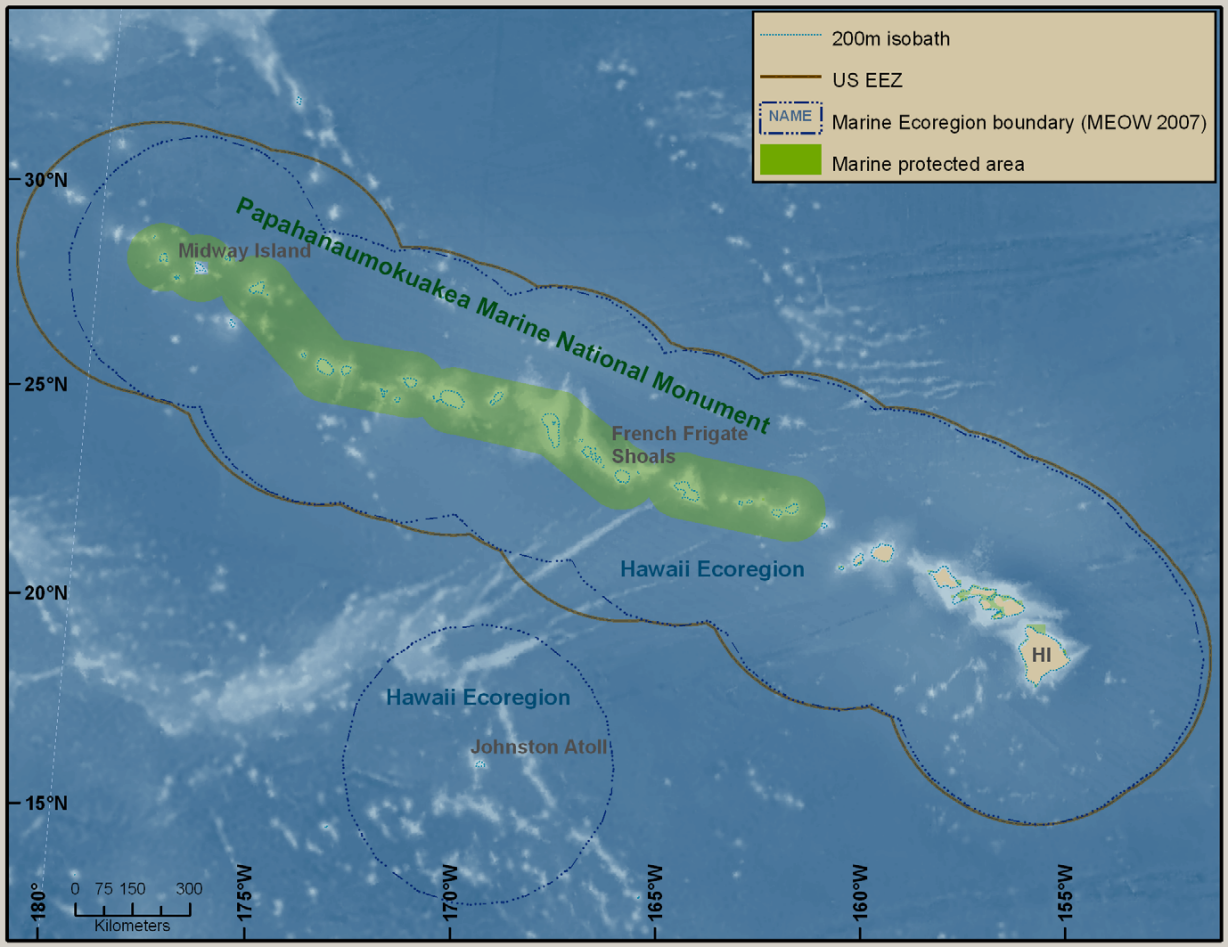
The Hawaiian Archipelago. Map shows the designation of the Marine protected areas, the Marine Ecoregion Boundary, and the U.S. EEZ.

**Figure 8:**
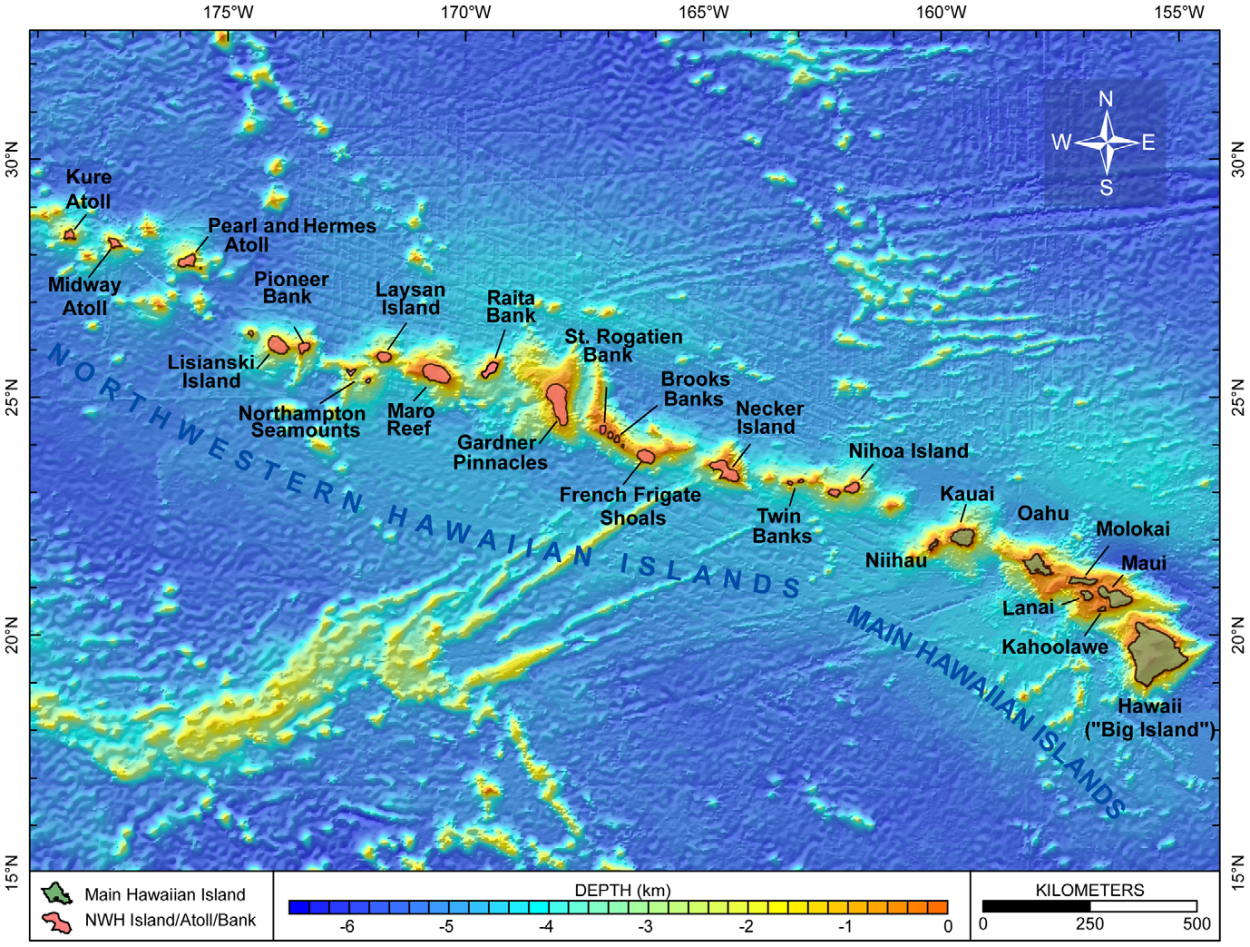
Bathymetric map of the Hawaiian Archipelago. Figure courtesy of [Rooney J, Wessel P, Hoeke R, Weiss J, Baker J, et al (2008) Geology and geomorphology of coral reefs in the northwestern Hawaiian Islands. In: Riegl BM, Dodge RE (eds). Coral Reefs of the USA. Springer, pp. 515-567]

The Hawaiian Archipelago consists of eight high volcanic islands with offshore nonstructural reef communities and fringing reefs abutting the shore [154] at its southern end, and a series of small islands, atolls, shoals, seamounts, and banks stretching to Midway and Kure atolls at its northwestern end (Figure 7). Excluding Midway, which is an unincorporated territory of the U.S., the Hawaiian Islands form the U.S. stat of Hawaii. The eight Main Hawaiian Islands (MHI), which are home to 99% of the state's 1.3 million human residents, are separated from the southernmost area of the mostly uninhabited Northwestern Hawaiian Islands (NWHI) (Figure 8), which span more than 2,000 km, by 250 km of open ocean. The archipelago extends 2,500 km astride the Tropic of Cancer between 154°40′ and 178°25′ W, and 18°54′ and 28° 15′ N. Its total land area is approximately 16,642 km^2^. About 3,000 km from the nearest continent, it is the most isolated group of islands on earth. Undersea mapping is ongoing, including annual multibeam surveys. The forereef slopes, between 20 and 500 m, have been extensively surveyed, but large areas of the shallow reefs and some bank tops are still unmapped (Pacific Islands Benthic Habitat Mapping Center at http://www.soest.hawaii.edu/pibhmc/pibhmc_nwhi.htm).

The Hawaiian Islands lie within the southern portion of the anticyclonic North Pacific Subtropical Gyre. Although mean surface currents are driven west by the northeasterly trade winds, variability of the flow is great. Because the high MHI cast a partial wind shadow in the easterly trade winds that blow almost year round, a narrow zone of weak winds develops on the lee sides of the islands. In the central part of these generally westward currents, the narrow and intermittent Subtropical Countercurrent flows eastward from the western Pacific [155], [156]. This current has been speculated to be a genetic gateway that is most likely responsible for some of Hawaii's flora and fauna [157], [158].

Eldredge and Abbott inventoried 8,427 species of fish, algae, coral, and other invertebrates (unpublished) (Table S6). Befitting its isolated location, Hawaii has estimated rates of endemism of 25% or greater for most coral reef species [159], [160], [161], [162], [163]. Results of the CReefs Expedition in November 2006 to the French Frigate Shoals, in the central portion of the Hawaiian Archipelago, which was the first dedicated biodiversity assessment carried out in the NWHI, suggest that the number of known species underestimates the true biodiversity.

#### Regional history of biodiversity studies

Several French and Russian ships visited the islands in the early 1800s. The U.S. Exploring Expedition, the first of the major exploration expeditions to have passed through the Hawaiian Islands, sailed in the Pacific between 1838 and 1842, making a stop in Hawaii in May-June 1841. Records of material collected on that trip are presented in numerous volumes. The *Challenger*, on its short stop in Hawaii in mid-1875, collected plankton and dredged in Pearl Harbor. The German biologist Hugo Schausinsland spent three months on Laysan; material he collected was the subject of numerous articles. The deep-dredging expedition of the *Albatross* Trans-Pacific cruise in 1891, under the leadership of Alexander Agassiz from Harvard, resulted in reports of many new species. The *Albatross* Expedition in 1902 initiated the first major collections from Hawaii. These were published in three volumes of the *Bulletin of the United States Fish Commission* for 1903: “Aquatic Resources of the Hawaiian Islands; I. Shore Fishes; II. Deep Sea Fishes and Commercial Fisheries; III. Miscellaneous Papers” (which include isopods, brachyuran and macrurans, hydroids, schizopods, nemerteans, sea stars, medusae, and polychaetes). This investigation established a baseline for further investigation, and some new species are still being described from this material. During his two-year Danish Pacific Expedition, Dr. Th. Mortensen visited the Hawaiian Islands, and the holothurian *Opheodesoma spectabilis* was collected and described from his collections in Pearl Harbor. In 1922, a plan was designed for a major survey of 13 Hawaiian Islands and Johnston, and Wake Island, with the *Tanager*, a Navy Department mine sweeper: four trips were made during 1923 and 1924. Material from the *Tanager* Expedition, primarily published in *Marine Zoology of Tropical Central Pacific* [164], included crustaceans, echinoderms, polychaetes, and foraminiferans. An expedition led by P.S. Galtsoff to Pearl and Hermes between July and September 1930, surveyed the abundance of pearl oyster for potential commercial use. He noted the corals, algae, sponges, mollusks, crustaceans, and echinoderms [165].

Since these early cruises, conducting inventories of the biota of Hawaii has largely been the responsibility of the Bishop Museum. Virtually all definitive published treatments and manuals of Hawaiian organisms, beginning with *Fauna Hawaiiensis* in 1901, have been produced by the Museum, or in close collaboration with it. In 1992, the Hawaii State Legislature recognized the Bishop Museum for these contributions and designated it as the Hawaii Biological Survey (HBS). Surveys have occurred in targeted sites in the MHI, such as Kaneohe Bay and Pearl Harbor on the island of Oahu, and waters around the island of Kahoolawe. Electronic datasets for Hawaiian marine biodiversity include: http://hbs.bishopmuseum.org/ (Hawaii Biological Survey); http://cramp.wcc.hawaii.edu/ (Reef Assessment and Monitoring Program); http://www.nbii.gov/portal/community/Communities/Geographic_Perspectives/Pacific_Basin/ (National Biological Information Infrastructure(NBII), Pacific Basin Information Node); and http://www.nbii.gov/portal/community/Communities/Habitats/Marine/Marine_Data_(OBIS-USA)/.

The Bishop Museum has conducted intensive biological inventories since 1995, covering all of MHI, Midway Atoll, French Frigate Shoals, and Johnston Atoll. The results of these inventories have appeared in numerous Technical Reports of the Bishop Museum, including a checklist of all species collected with museum records of previously collected specimens. Many specialists have been consulted for the determination of species, and voucher specimens have been deposited in the Bishop Museum. Initial information on the numbers of Hawaiian species can be found in Eldredge [166].

Fishes have been of interest for many years. Jordan and Everman [167], in their introduction to the *Albatross* Expedition, reported on all previous collections of fishes and added many more. Gosline and Brock [168] presented information on the zoogeography of Hawaiian fishes and provided keys to species. In 1993, a popular book on Hawaiian fishes by Hoover [169] was published. A checklist of fishes of the Hawaiian Archipelago, recording some 1,250 species and including the history of ichthyology in the Hawaiian Islands, was published by the Bishop Museum [170]. Most recently the fishes of Hawaiian waters were thoroughly reviewed in a handsome all-color volume [171].

Stony coral investigations began with Vaughan's [172] monograph on the recent corals of the Hawaiian Islands. This was based primarily on the *Albatross* 1902 collections, but also included some from the U.S. Exploring Expedition, and other material. This and more recent collections were the basis of a review of the scleractinians along with a key to species [173]. An updated species list has also been published [158], as well as a field guide to the Hawaiian corals [174]. Cairns has revised the hydrocorals and ahermatypic corals [175].

The crustaceans have been fairly well documented. Rathbun [176] described the specimens from the *Albatross* 1902 Expedition. Numerous publications resulted from the four decades of investigations of C. H. Edmondson of the Bishop Museum. More recent work has been based on museum material collected also over decades. A series of papers based on collections made in 2006 under the auspices of the Census include descriptions of more than100 new species [177], [178], [179] from French Frigate Shoals; additional reports are in preparation. Eldredge (unpublished) has prepared an authoritative list of the anomuran and brachyuran fauna of the Hawaiian Islands.

The main molluscan study is that of Kay [180], who provided a history of collecting in the islands, as well as a section on biogeography. Numerous additional species have been reported in individual publications and revisionary works. Other groups of invertebrates are less well studied. Opresko has revised the antipatharians in a series of papers [181]. There is scattered information on many groups but no one source of information on the status and biogeography of the Hawaiian marine biota.

Marine algae are among the most poorly understood organisms in Hawaiian reef ecosystems, yet without them coral reefs could not exist. Their importance to Hawaiian ecosystems is staggering: algae form the base of the food chain, occupy much of the benthic substrate, and help oxygenate the water. Coral-to-algal phase shifts that occurred in the Caribbean [182] have caused many reef researchers to erroneously assume that diverse and abundant algal populations in reef settings are detrimental, but new research is documenting the importance of algal populations to healthy reef systems [183], [184], [185], [186]. Comprehensive species lists of algae are just beginning to be assembled for most islands and banks of the Hawaiian Archipelago [186], [187], and most research expeditions add to knowledge of algal diversity. The largest gaps in our understanding of the Hawaiian marine flora are linked to (a) a lack of trained algal taxonomists, (b) public and government apathy toward the study of marine plants, and (c) difficulty of access to many environments. When trained phycologists explore underexamined reef areas, even on the heavily populated island of Oahu, species new to science are regularly discovered [183], [188], [189], [190], and suspected cryptic diversity is only beginning to be investigated [191], [192].

Archaeological evidence reveals that seafood, particularly coral reef species, was part of the customary diet of the earliest human inhabitants of the Hawaiian Archipelago. The sea also provided medicines recognized by Western scientists today [193], [194]. The social and symbolic values of fish include early Native Hawaiian traditions related to the sharing of fish in the extended family and community. The importance of sharing fish is currently found in other ethnic groups in Hawaii, and many customs continue in today's modern and traditional fisheries. Hawaii's commercial fishing-based economy for nearly two centuries was based on pelagic fisheries (tuna), with contributions from precious coral, crustacean, and bottomfish fisheries. Most of the commercial, recreational, and subsistence catch of fishes, invertebrates, and seaweed comes from nearshore reef areas around the MHI, but over half of bottomfish are caught in federal waters surrounding the MHI, and Kona crabs come from Penguin Bank. The lobster fishery, consisting of mainly spiny lobster, *Panulirus marginatus*, and slipper lobster, *Scyllarides squammosus*, was confined to the NWHI until the fishery started to collapse in the early 1990s; it was closed in 1993, and remains closed because stocks have not recovered. Black corals continue to be collected by scuba divers from 30 to 100 m in the MHI, whereas the collection of other precious corals in Hawaii has been limited. The commercial aquarium fishery is now Hawaii's major inshore fishery; landings are reported as more than 220,000 specimens, with a wholesale value of $1.93 million in 2006 [154]. Its retail value can be estimated conservatively at more than $10 million, based on the retail value of yellow tangs (*Zebrasoma flavescens*), each of which sells for at least $25; in 2006, 366,317 yellow tangs were reportedly collected in Hawaii.

#### The known, unknown, and future directions

The Hawaiian Islands are among the best biologically known islands in the Pacific Ocean, many publications having dealt with the general marine biota. In 1933, C.H. Edmondson first published *Reef and Shore Fauna of Hawaii* [165], which included invertebrates as well as fishes. He revised this book in 1946 without the fishes [195]. A popular book, *Seashore Treasures*, followed in 1949 [196]. Fielding and Robinson [197] prepared an underwater guide mainly to the most common species. The most complete field guide to the marine invertebrates was revised by Hoover [198]. Hoover [199] wrote another field guide to the fishes, sea turtles, dolphins, whales, and seals. Still, it is likely that large numbers of new species remain to be discovered throughout the archipelago, where known endemism is the highest of any tropical marine ecosystem on earth [171], [200], [201].

Two workshops on “Marine and Coastal Biodiversity in the Tropical Island Pacific Region,” held in November 1994 in Honolulu, resulted in two publications—Volume 1: Species systematics, and information management priorities and Volume 2: Population, development, and conservation priorities. Volume 1 includes 13 sections on the status of various taxonomic groups authored by specialists who provide information on numbers of species and their biogeography [202].

Studies of Hawaiian algae during the past decade have greatly increased understanding of species diversity and species ranges and led to the publication of three major works: *Marine Red Algae of the Hawaiian Islands* [203], *Marine Green and Brown Algae of the Hawaiian Islands* [204], and *Hawaiian Reef Plants* [205]. At French Frigate Shoals alone, recent work has increased the number of documented species by 380% [187] and led to the discovery of two species new to science [189], [190]. Archipelago-wide studies are revealing nuances in algal biogeography; some species prefer specific habitat types, and some are adapted to cold winter temperature regimes found in the northernmost areas of the Hawaiian Archipelago (Vroom and Braun, in review). Many algal species in the Hawaiian archipelago are pantropical, whereas others share affinities with the Japanese, Australian, or Indo-western Pacific floras [203], [204]. For species that occur in Hawaii and elsewhere, it is often unclear whether their distributional patterns are natural or are the result of introductions. Some species that occur in disparate geographic locations have turned out to represent genetically distinct species that have converged on similar morphologies. Molecular research [191], [192], [206], [207] is greatly helping in understanding both these types of situations and may reveal that many Hawaiian species thought to be representatives of Caribbean or Indo-western Pacific taxa are new species endemic to the Hawaiian Islands.

Recent studies in the NWHI suggest that French Frigate Shoals is where coral reef diversity is highest in the Hawaiian Archipelago, and there is evidence that species arrived here from the southern Pacific via Johnston Atoll [208]. The first marine biodiversity survey with the taxonomic expertise to assess reef taxa over a broad range of flora and fauna was the CReefs Expedition. Although comprehensive biodiversity surveys and analyses are desirable to follow up on this survey, they remain uncertain based on permitting concerns. French Frigate Shoals has the largest number of species of *Acropora*, the major reef-building coral of the rest of the Indo-Pacific; these corals have not been observed south of Kauai in the MHI.

Assessing biodiversity of Hawaiian coral reefs has been difficult because of limited financial and logistical resources, severe shortages of trained taxonomists, and the subjectivity and biases of methods. These challenges are particularly problematic for the small and cryptic invertebrate taxa, among which the greatest diversity is likely to occur. Many techniques and types of equipment are needed to collect mobile and sessile, infaunal and epifaunal, and pelagic and benthic organisms in a variety of habitats that encompass various depths, exposures to wave energy, and other environmental forcing [209]. Although comprehensive CReefs-type biodiversity assessments are desirable at representative regions and habitats across the Hawaiian Archipelago, and elsewhere across the Pacific Islands, such efforts are generally too costly and require more extensive taxonomic expertise and curatorial capacity than exists in the region.

Resource limitation was a problem even for the CReefs Expedition. Although funded adequately for operations, it had inadequate resources for thorough post-cruise processing and analyses of the specimens collected. Techniques used, including such time-honored ones as yabbie pumps and trawls, are described in detail at http://hawaiianatolls.org/research/CoML/collection.php. These techniques were supplemented with GPS position data and with before-and-after photographs at each site. Budgetary constraints and the urgent need for baseline biodiversity assessments prior to additional, and potentially dramatic, biodiversity shifts occurring in response to climate change, particularly ocean acidification, have allowed CReefs and the Pacific Islands Fisheries Science Center Coral Reef Ecosystem Division of NMFS to lead the development, testing, and implementation of Autonomous Reef Monitoring Structures (ARMS) as a standard method to collect sessile or sedentary biota in a reproducible manner [210]. Diversity of specimens collected by ARMS can be assessed using molecular techniques, which can be more rapid than morphological analyses [211].

In October 2006, 12 ARMS were deployed in four sets of three replicates at a backreef site, a lagoon patch reef site, and two forereef sites at French Frigate Shoals. Recovered and analyzed in October 2007, the collection included mollusks (28%), ascidians (24%), crustaceans (19%), and bryozoans (11%). Two non-native solitary tunicates, *Cnemidocarpa irene* and *Polycarpa aurita*, were new records for the NWHI [212]. Crustacean biodiversity was characterized through DNA. The 12 ARMS recovered from the NWHI and 7 recovered from the south shore of Oahu provide confidence that cryptic invertebrate fauna collected are representative of the habitat and intrasite variability is acceptably low. Preliminary results from 9 ARMS recovered in February 2009 from a CReefs site at Lizard Island, Great Barrier Reef, also indicated low intrasite variability (M. Timmers, personal communication). Thus, coupling ARMS with morphological and molecular analyses can be effective in assessing some components of coral reef invertebrate biodiversity.

There is a need for more surveys in different geographic regions, habitats, and depths throughout the Hawaiian Archipelago. In particular, baseline assessments must be made so that invasive species, which are a serious problem in some areas of the MHI, can be detected before alterations in the biodiversity occur. Regions in the MHI that are more remote should particularly be the subject of exploration. Current knowledge of the biota of the Insular Pacific- Hawaiian LME is summarized in Table 7 (more detail is available in Table S1).

**Table 7:** Biotic diversity in the Insular Pacific–Hawaiian Large Marine Ecosystem.

Taxonomic group	No. species^1^
**Domain Archaea**	**UD**
**Domain Bacteria (including Cyanobacteria)**	**UD (183)**
**Domain Eukarya**	**8,244**
**Kingdom Chromista**	**175**
Phaeophyta	84
**Kingdom Plantae**	**821**
Chlorophyta	247
Rhodophyta	574
Angiospermae	UD
**Kingdom Protoctista (Protozoa)**	**798**
Dinomastigota (Dinoflagellata)	43
Foraminifera	755
**Kingdom Animalia**	**6,395**
Porifera	144
Cnidaria	460
Platyhelminthes	676
Mollusca	1345
Annelida	343
Crustacea	1325
Bryozoa	168
Echinodermata	309
Urochordata (Tunicata)	102
Other invertebrates	228
Vertebrata (Pisces)	1214
Other vertebrates	81
**TOTAL REGIONAL DIVERSITY** 2	**8,427** 3

**Notes:**

1 Sources of the reports: databases, scientific literature, books, field guides, technical reports, and personal communication with taxonomic experts.

2 Includes all taxonomic groups as reported in Table S1.

3 Includes 55 species of fungi.

UD  =  Listed in work but number undetermined to date.

#### Trouble spots and emerging issues

Despite a wealth of potential new species, the Hawaiian LME (Figure 7) is considered depauparate. Its coral reefs have significantly fewer species of fishes, corals, and algae than those of Indonesia and Australia (see Table 8 and [213]). In Hawaiian environments, redundancy in ecosystem function is probably low, and there are concerns that it is a relatively fragile ecosystem [214]. The Archipelago's isolation, relatively low species diversity, and high endemism may make the system comparatively non-resistant to perturbations, such as invasion by alien species, human use, and pollution.

**Table 8:** Comparative species numbers of selected groups in Indonesia, Australia and Hawaii.

	Indonesia^a^	Australia^b^	Hawaii^c^
Fish	3,000+	1,500+	1,214+
Corals	700+ (450)	400+ (359)	460 (99 scleractinian)
Algae	n/a	3,000+	500+

**Notes:**

a Fenner D (2002) Reef corals of the Raja Ampat Islands, Papua Province, Indonesia. Part II. Comparison of individual survey sites. Appendix 2. Coral species recorded at individual sites in the Raja Ampat Islands. In: McKenna SA, Allen GA, Suryadi S, editors. A marine rapid assessment of the Raja Ampat Islands, Papua Province, Indonesia RAP Bulletin of Biological Assessment 22. Washington, D.C.: Conservation International. pp. 29-36, 104-112.

b CRC Reef Research Centre (2008) REEF FACTS: Plants and animals on the Great Barrier Reef. Huisman JM (2000) Marine plants of Australia. Nedlands, Australia: University of Western Australia Press. 300 p.

c Abbott IA (1999) Marine red algae of the Hawaiian Islands. Honolulu: Bishop Museum Press. 477 p.

Abbott IA, Huisman JM (2004) Marine green and brown algae of the Hawaiian Islands. Honolulu: Bishop Museum Press. 259 p.

Huisman JM, Abbott IA, Smith CM (2006) Hawaiian reef plants. Honolulu, Hawaii: University of Hawaii Sea Grant. 264 p.

Endemics dominate abundance assessments in many NWHI communities [163], and because many endemics have narrow habitat and physiological tolerances, they may fare worse under climate change than wide-ranging species [215]. The high density of some endemics may allow quick recovery from localized pulsed disturbances, but they may render the habitat vulnerable to large-scale, constant impacts like marine debris, global change factors, and fishing, contributing to the NWHI's probable low resistance and resilience. Populations of some irreplaceable species, like Hawaiian monk seals and some seabirds that are already listed as critically endangered, may have larger impacts on the ecosystem than is realized at the moment.

Current studies indicate that Hawaii's endemic species have evolved to provide multiple ecosystem services (functional compensation). The question of whether this is a common phenomenon in depauparate systems, especially in island environments, requires an assessment of biodiversity, coupled with ecosystem functional analysis [2]. Few studies have examined the effect of genetic heterogeneity, a component of biodiversity that controls whether organisms have the genetic potential to adapt to environmental change, on ecosystem resilience in the marine environment.

An assessment of the trouble spots in the Hawaiian ecosystem was published by Selkoe, Halpern, and Toonen [216], who developed a threat-ranking system that identified areas of concern in the NWHI. Maro Reef emerged as the region of primary concern [217]. Although this island and similar ones are protected to some extent by the designation of the Papahānaumokuākea Marine National Monument in 2006 and the closing of all fishing in the area by 2011, they are threatened by potential impacts of global change, which is likely to affect marine ecosystems across the entire Hawaiian Archipelago. Global change models predict that threats to Hawaii's ecosystem include sea level and temperature rise, and pH decline [218], [219].

#### The Census of Marine Life contribution to the Insular Pacific-Hawaiian region

A major contribution to knowledge of Hawaiian marine biodiversity was the CReefs survey of the French Frigate Shoals. During the 16 survey days, at least 2,025 “unique morphospecies” were collected. Taxonomic experts are in the process of identifying the collected specimens, with a final tally projected to reach more than 100 newly described taxa (including species, genera, and families).

### California Current Large Marine Ecosystem

#### Description of the California Current region

**Figure 9:**
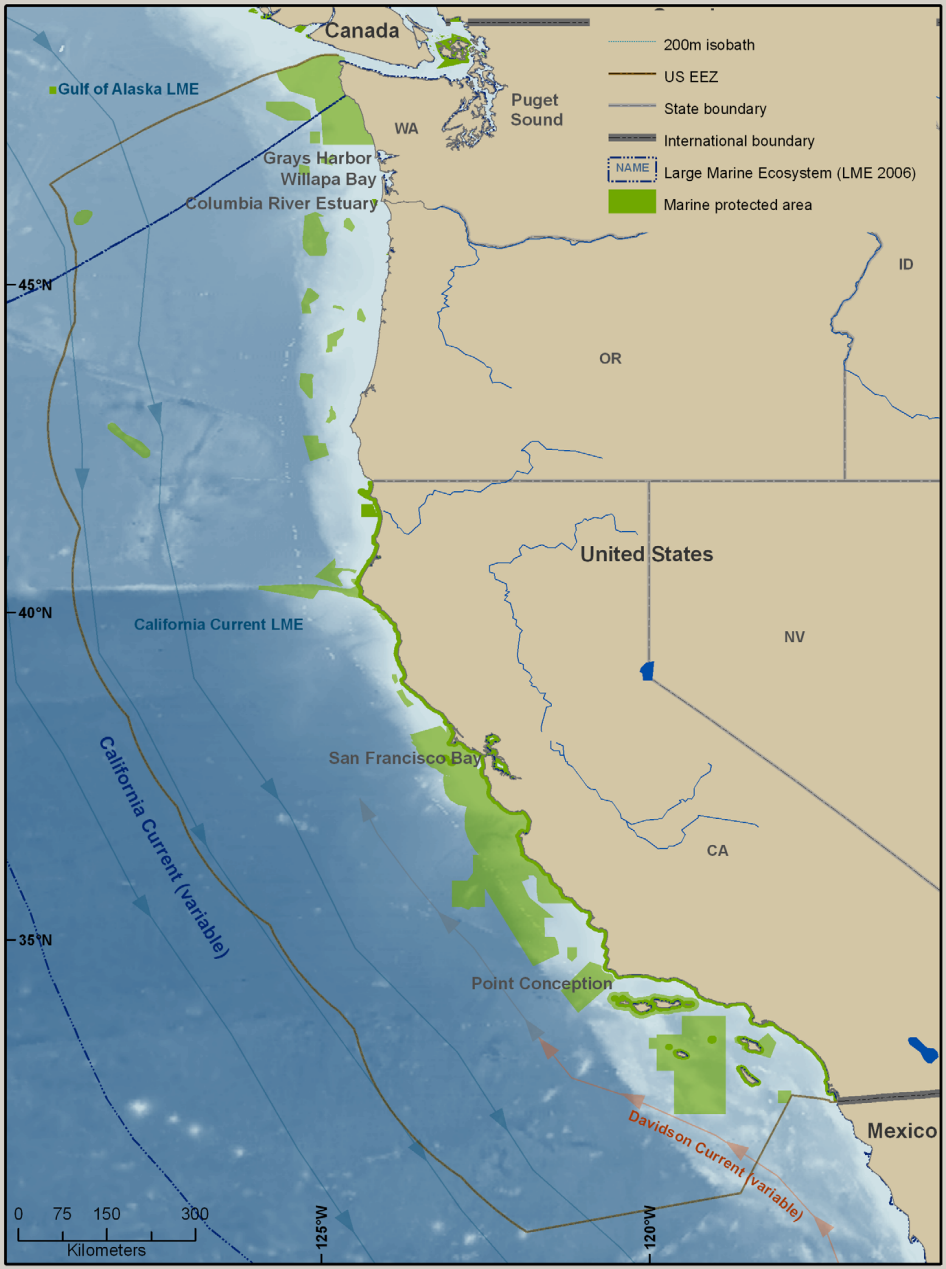
California Current Large Marine Ecosystem (CCLME). Map also shows EEZ boundaries, state boundaries, international boundaries, marine ecoregions, marine protected areas, major embayments, bathymetry and major oceanic currents.

The California Current LME stretches from Baja California to Vancouver, British Columbia, and encompasses the shorelines and offshore ocean environments of Washington, Oregon, and California (Figure 9). The climate is mild and maritime with dry summers and cool, rainy winters. From north to south, average temperature increases and annual rainfall decreases. The region is strongly affected by seasonal and interdecadal climate variability, such as El Niño events and the Pacific Decadal Oscillation. Coastal areas are extremely variable, ranging from the Olympic Peninsula's mountains and misty rainforests to southern California's arid beaches.

Two features dominate the region's marine bathymetry: a narrow continental shelf and a large, deep ocean basin [220]. From Vancouver Island south to Point Conception, the continental shelf is generally 48-68 km wide. Along the Washington and California coasts, submarine canyons transect the shelf and the slope, but along Oregon, canyons are absent. There are only a few large bays, the largest of which is Puget Sound with 6,400 km^2^ of water, over 3,700 km of shoreline, and hundreds of islands. Other major embayments include Grays Harbor, Willapa Bay, the Columbia River Estuary, and San Francisco Bay, including the estuaries of the Sacramento and San Joaquin rivers. These systems are important migratory and nursery areas for fishes and invertebrates.

The coastal region can be subdivided into three physically and ecologically distinct zones [220]. The northern portion, part of the Oregonian Province Bioregion, is strongly influenced by the colder waters of the southward flowing California Current, and has water temperatures that range generally from 5 to 15°C. It also is characterized by a relatively undivided continental shelf, considerable runoff, especially from the Columbia River, and extremely complex flow patterns within the California Current system, including upwelling. The plume of the Columbia River, one of the continent's largest, varies seasonally and exerts influence over a broad area at the Washington-Oregon border. The central zone, also part of the Oregonian Province, has much less freshwater input, a narrower continental shelf, strongly seasonal upwelling, and more consistent southward flow offshore. Across these two areas, shorelines vary extensively, from Puget Sound's protected, deep-water fjords and inlets to the outer coast's mixture of islands, submerged reefs, rocky cliffs and headlands, cobble and boulder fields, expansive dunes and sandy beaches, estuaries, embayments, and lagoons. The ecosystem features diverse marine and coastal habitats that support a wealth of living marine resources characterized by temperate marine flora and fauna.

The southern zone, south of Point Conception, is a defined part of the San Diegan Province; it is characterized by warmer temperatures as a result of the east-west orientation of the California coast, where the core of the California Current is farther offshore. Known as the Southern California Bight, this region is influenced by the warmer waters of the Davidson Current, as well as by the California Current. Two water masses converge at Point Conception, creating a transition zone with high species diversity, where many marine species reach their northern or southern range limits. The continental shelf is narrow and the continental border is a complex of islands, banks, and deep basins.

Offshore, marine circulation is the major factor determining species distribution patterns. Summer surface temperatures range from 13 to 20°C and winter temperatures from 8 to 17°C. Annual primary productivity is moderate to high, with a peak in late spring to early summer (March-May). Nutrients come from upwelled water, and productivity is likely to be limited by nitrogen. Shelf influence is negligible. Many regional species are endemic, such as northern anchovy and Pacific hake [220].

High coastal productivity translates into high marine diversity and numerous species of social and economic importance. More than 40 federally recognized Native American tribes inhabit this region; marine species are central to the heritage, culture, and quality of life of many of them. Marine species like killer whales (*Orcinus orca*) have assumed totemic importance in tribal and Northwest culture. Once supporting major commercial, recreational, and tribal fisheries, six salmonid species have been a keystone of western coastal economies and traditions: sockeye salmon (*Oncorhynchus nerka*), Chinook salmon (*O. tshawyscha*), coho salmon (*O. kisutch*), chum salmon (*O. keta*), steelhead (*O. mykiss*), and pink salmon (*O. gorbuscha*).

In addition to salmon, commercial and tribal fishery resources include important invertebrate populations, particularly in nearshore and estuarine waters, significant stocks of groundfish along the continental shelf, and large populations of pelagic and highly migratory species [221]. These include important regional fisheries for Dungeness crab (*Cancer magister*), rockfish (*Sebastes* spp.), albacore tuna (*Thunnus alalunga*), Pacific hake (*Merluccius productus*), sablefish (*Anoplomoma fimbria*), northern anchovy (*Engraulis mordax*), Pacific sardine (*Sardinops sagax*), market squid (*Loligo opalescens*), and sea urchins (*Strongylocentrotus* spp.) [222]. The Northwest supplies about half of the U.S. production of oysters and there are major aquaculture operations for both native and introduced shellfish species. There are also important populations of marine mammals and seabirds, and tourism centered on whale watching and wildlife viewing is another economically important activity, particularly in southern California and in Puget Sound.

#### Regional history of biodiversity studies

The history of research and species discovery in the California Current LME follows the general pattern observed in other coastal areas of the U.S. For thousands of years, seafarers and coastal native populations relied on abundant living marine resources, particularly marine mammals, salmon, and shellfish. Many of the indigenous communities continue to hold important ecological knowledge. In the late eighteenth and early nineteenth centuries, European explorers, including Cook, La Perouse, Vancouver, and Bodega y Quadra, focused on mapping the largely unknown northwest territory [223]. In the mid-nineteenth century, U.S. naval expeditions led by Perry and Maury collected fish specimens and other information, such as whale sightings, on living marine resources. The growth of commercial whaling and sealing during the same period yielded increased data on the distribution of target populations, as thousands of whales and hundreds of thousands of walruses, seals, sea lions, and Steller's sea cows were hunted and killed.

The twentieth century saw the establishment of marine research laboratories throughout the region, many of which continue to operate. In 1892, Stanford President David Starr Jordan oversaw the opening of the Hopkins Seaside Laboratory in Monterey, California. Today the Hopkins Marine Station is one of about two dozen academic, federal, and state marine research facilities that participate in the Monterey Bay Crescent Ocean Research Consortium. The Consortium uses the Bay as a natural laboratory to promote the scientific understanding of coastal and marine systems and to facilitate the application of that knowledge for public policy, environmental awareness, and decision making. Similar infrastructure development has occurred throughout the region. In 1904, University of Washington professors Trevor Kincaid and T.C. Frye established Friday Harbor Laboratories in the San Juan Islands. Today the Western Association of Marine Laboratories has 20 West Coast members, and there are an estimated 40-50 marine research facilities in the region, a research staff of several hundred, and a sizable government and academic research fleet.

Numerous reviews and syntheses of various taxonomic groups have been written for the region, focusing particularly on nearshore species and those of commercial significance (e.g., [224], [225], [226], [227], [228], [229], [230]). General information on larger and better-known species, such as marine mammals and seabirds, also is readily available [231], [232]. Table 9 summarizes those data; more detail is available in Table S1.

**Table 9:** Biotic diversity in the California Current Large Marine Ecosystem.

Taxonomic group	No. species^1,2,3^
**Domain Archaea**	**UD**
**Domain Bacteria (including Cyanobacteria)**	**UD**
**Domain Eukarya**	**10,160**
**Kingdom Chromista**	**187**
Phaeophyta	187
**Kingdom Plantae**	**703**
Chlorophyta	139
Rhodophyta	557
Angiospermae	7
**Kingdom Protoctista (Protozoa)**	**896**
Dinomastigota (Dinoflagellata)	UD
Foraminifera	670
**Kingdom Animalia**	**8,374**
Porifera	134
Cnidaria	400
Platyhelminthes	1389
Mollusca	663
Annelida	830
Crustacea	2680
Bryozoa	150
Echinodermata	290
Urochordata (Tunicata)	62
Other invertebrates	733
Vertebrata (Pisces)	909
Other vertebrates	134
**TOTAL REGIONAL DIVERSITY** 4	**10,160** 5

**Notes:**

1 Sources of the reports: databases, scientific literature, books, field guides, technical reports, and personal communication with taxonomic experts.

2 Identification guides cited in Text S2.

3 Taxonomic experts cited in Text S4.

4 Includes all taxonomic groups as reported in Table S1.

5 Includes 198 parasite-only other protozoans (Haplosporida, Microsporida, Myxosporida, Sarcomastigophora, Sporozoa).

UD  =  Listed in work but number undetermined to date.

While significant capacity exists on the West Coast for collection of biodiversity information, existing activities are generally not integrated, have limited geographic coverage, and sample infrequently. Nearshore and intertidal information is collected at local scales, and integrating observations can be difficult [221]. Much of the available long-term data is a product of fishery management efforts, and a substantial proportion is funded through NOAA. Interest in integrated ocean observing systems, ecosystem approaches to management, and assessment of regional environmental change have sparked renewed interest in existing long-term datasets and spurred new partnerships to collect and integrate marine data. Potentially useful databases for biodiversity information are listed in Table S4.

#### The known, unknown, and future directions

As a Census contribution, the North Pacific Marine Science Organization (PICES) recently completed an overview of what is known and unknown with respect to marine biodiversity in the North Pacific Ocean [233]. It concludes that regional knowledge is based on aggregate values derived from limited coastal sampling and detailed information related to commercially important species, or proximity to a marine science facility. The report discusses six categories of marine life, focusing on taxonomy, geographic distribution, abundance, life history, productivity, and variability. A summary of the PICES report is provided below, along with taxon-specific information on the state of knowledge regarding biodiversity in the California Current system, where available, from personal communications with taxonomic experts.

Bacterioplankton may be very abundant, numbering about 3.1×10^28^ single-celled organisms in the world ocean. Growth is controlled by dissolved organic carbon and, in surface waters, temperature. Cyanobacteria such as *Synechococcus* are important in the California Current region and some species fix nitrogen. Identification of abundant groups in bacterial communities is important in assessing roles in carbon cycling and ocean biogeochemical processes, and as a component of some marine food webs. The role and importance of bacterioplankton are largely unknown, because of poorly defined taxa and a basic lack of core census information [233].

Ocean-color-sensing satellites measuring productivity are the primary source of phytoplankton information. The California Current system is less productive than similar South American upwelling systems because of the breadth of the shelf and differences in wind stress. Diatoms often dominate regional phytoplankton species composition; *Coscinodiscus*, *Nitzschia*, and *Tripodonesis* species form 81% of the biomass, particularly in upwelling areas. Although phytoplankton is better known than marine bacteria, critical unknowns include smaller organisms, temporal and spatial variability, and the dynamics of species composition and harmful algal blooms [233].

The rich seaweed flora of the Pacific coast has been extensively explored and cataloged. The accuracy of biodiversity estimates is affected by limited knowledge of deep communities, of microscopic forms, of species that have been collected rarely, and of species complexes that need further study, especially with molecular methods. Future studies will reveal that some species represent complexes of multiple species, while some species should be merged. The calcified red seaweeds (Corallinales) are a good example of a group that will profit from extensive revision. Many species on the West Coast were described from distant parts of the world; as a consequence, it is likely that names have been misapplied and revisions will reveal a higher degree of endemism (K.A. Miller, personal communication).

The zooplankton is generally better known than bacterioplankton and phytoplankton, although limited information is available on the species composition and ecology of smaller zooplankters. In the 1990s, dramatic shifts in species composition between large cold-water and small warm-water taxa mirrored environmental oscillations in the northern areas of the California Current. Shelf copepods, including species both large (*Calanus marshallae, Acartia clausii*, and *A. longemirus*), and small (e.g. *Pseudocalanus* species and *Oithona similas*), dominate this biota. Knowledge is limited with respect to distribution and abundance of rare species, zooplankton productivity, spatial and temporal variability, gelatinous zooplankton, midwater oceanic shrimps, and deep oceanic zooplankton [233].

Invertebrates are fairly well known, at least for the macrofauna of the continental shelf. However, deep-sea species are poorly known and there is little information on the life histories or biogeography of noncommercial species. In addition, few estimates of benthic productivity exist, and information on spatial or temporal variability exists for only selected areas [233]. What is known and unknown also varies with taxon:

- The richest component of the cnidarian fauna on the continental shelf appears to be hydrozoans, which make up just over half the known species of the phylum (greater than the proportion of hydrozoans in the phylum worldwide). Cnidaria are best known in the intertidal zone; knowledge declines with depth, and for some groups, like anemones, the deep fauna is almost unknown. This is especially problematic for the West Coast, where the continental shelf is narrow, so most of the subtidal benthic area within the region is deep (D. Fautin, personal communication).
- According to Crandall and Norenburg [234], [235] there are 137 species of Nemerteans in the Pacific region of the U.S. (not including Alaska or Hawaii), 119 of which are benthic and 18 bathypelagic. This number is likely an underestimate, based on the rate of discovery of undescribed species. For example, about 50 species are known from Central California to Oregon [233] and recently at least 10 species collected from Oregon opportunistically are either new to the area or possibly to science (S. Maslakova, personal communication). This is also true for other areas in the U.S. Pacific Coast.
- West Coast Gastrotricha species are vastly understudied; only 8 species have been published from the intertidal and shallow coastal waters of California and Washington. No gastrotrichs were reported from Oregon until 2001, when Hummon recorded 30 species in 12 genera [224].
- Most nematodes are known from either the holdfasts of intertidal kelp or from intertidal sediments. Nematodes inhabit virtually all habitats, so it is likely that many other species are either undescribed or unrecognized in this region [224].
- Numbers for Echinodermata are likely to be conservative, with new taxa and biogeographic records to be added as regions below 100 m are explored. This will be especially true for brittlestars and sea cucumbers, as these are less well known from abyssal regions than are, for example, the sea urchins. Distribution of species among the five major clades is approximate and as follows: Crinoidea (sea lilies and feather stars), 10; Asteroidea (sea stars), 100; Ophiuroidea (brittlestars and basketstars), 60; Holothuroidea (sea cucumbers), 40; Echinoidea (sea urchins, heart urchins, and sand dollars), 80 species (R.J. Mooi, personal communication).

More is known about fishes than most other species groups because of their larger size and use as a sustainable resource. There are an estimated 912 West Coast fishes (J. Orr, personal communication), of which 7 are agnathans (hagfish and lampreys) and 80 are chondrichthyians (sharks, rays, and ratfish). Also included are 11 species that have been recognized but not yet named. Primary unknowns for fishes in general include the life histories of many species, productivity estimates, and spatial and temporal variability [233].

Seabirds and marine mammals are perhaps the two best-known groups because they are relatively large and charismatic, many are of economic importance, and certain species, particularly those that breed on land, can be easily observed and tracked. A total of 92 species of seabirds has been recorded on the West Coast [231]; of these, 52 are associated with the northern California Current and 49 with its southern reach [233]. Thirty-eight marine mammal species can be found within the region [232], 16 in the north and 30 in the south [233]. The list of marine mammal species was considered complete until 2002, when a new species of beaked whale was identified from genetic analysis of five animals stranded on the California coast. As with other groups of species, unknowns for both seabirds and marine mammals include species productivity and variability [233].

#### Trouble spots and emerging issues

Over the past decade, marine endangered species have emerged as an increasingly serious problem with wide-ranging societal implications. At present, about 25 species of West Coast marine mammals, seabirds, turtles, fish, and shellfish are listed as threatened or endangered under the federal Endangered Species Act (http://www.nmfs.noaa.gov/pr/pdfs/esa_factsheet.pdf). Many of the listed whales and turtles are migratory oceanic species that were designated at the time the law was enacted (http://www.nmfs.noaa.gov/pr/pdfs/esa_factsheet.pdf). Over a little more than a decade, however, the depletion of salmon and steelhead runs (as a result of dams, overfishing, loss of habitat, and hatchery fish interactions) has led to the need to protect dozens of salmonid populations. Today, 52 evolutionarily significant units are recognized for the 6 regional salmonid species (http://www.nwr.noaa.gov/ESA-Salmon-Listings/Salmon-Populations/); 5 species are designated as endangered and 23 as threatened (http://www.nwr.noaa.gov/ESA-Salmon-Listings/upload/snapshot-7-09.pdf). Hundreds of millions of dollars are being spent to restore critical habitat, and serious consideration is being given to dam removals. Federal managers recently concluded that current water-pumping operations in California's Federal Central Valley Project and the California State Water Project should be changed to ensure survival of winter and spring-run Chinook salmon, Central Valley steelhead, the southern population of North American green sturgeon, as well as southern resident killer whales that rely on Chinook salmon runs for food [236]. In response, the federal government will spend $109 million to construct a pumping plant to allow salmon and green sturgeon unimpeded passage (http://www.noaanews.noaa.gov/stories2009/20090604_biological.html). While the primary focus currently is on salmon, other listed and candidate species are on a collision course with human development and activities in the region.

Overfishing continues to be a problem for salmon. The U.S. Department of Commerce in April 2009 extended a disaster declaration for the California and Oregon fisheries in response to expected poor salmon returns in the Sacramento River (http://www.noaanews.noaa.gov/stories2009/20090430_salmon.html). In a typical year, about half a million fall-run Chinook return to the river to spawn (http://www.noaanews.noaa.gov/stories2009/20090430_salmon.html). In 2007 and 2008, poor ocean conditions and overreliance on hatchery fish reduced returns to a fraction of that number [237], necessitating a near closure of the Oregon and California fisheries.

The West Coast groundfish fishery includes more than 90 species of rockfish, flatfish, roundfish, sharks, and skates (http://www.pcouncil.org/facts/groundfish.pdf). Seven species (widow rockfish, canary rockfish, yelloweye rockfish, darkblotched rockfish, bocaccio, Pacific ocean perch, and cowcod) currently are overfished and subject to rebuilding efforts (http://www.pcouncil.org/facts/groundfish.pdf). While rockfish populations in some locales are relatively healthy, others face severe localized depletions. Three rockfish populations in Puget Sound and the Strait of Georgia currently are being considered for listing under the Endangered Species Act as a result of overfishing (http://www.nmfs.noaa.gov/mediacenter/docs/04_22_2009.pdf). In the Georgia Basin, canary and yelloweye rockfish are proposed for threatened status, and bocaccio is proposed for endangered status (http://www.nmfs.noaa.gov/mediacenter/docs/04_22_2009.pdf).

The top headline in the June 14, 2009, *Seattle Times* asked “Is the Pacific Ocean's chemistry killing sea life?” (http://seattletimes.nwsource.com/html/localnews/2009336458_oysters14m.html). The West Coast shellfish industry is facing a fifth consecutive year of oyster hatchery failures, and scientists are beginning to examine possible links to ocean acidification (http://seattletimes.nwsource.com/html/localnews/2009336458_oysters14m.html). Other regional climate change concerns are alteration in coastal habitats as a result of sea level rise, changing ocean water circulation and upwelling patterns, shifts in the abundance and distribution of marine species, and increased incidence of harmful algal booms and other nuisance species [222].

Aquatic invasive species (AIS) are nonindigenous species that threaten the diversity or abundance of native species, the ecological stability of infested waters, or human activities that depend on such waters. On the West Coast, the introduction and spread of AIS have emerged as major environmental, economic, and public health problems tied to expansion in international trade and transportation [222]. Recent studies suggest that AIS are a significant threat to biodiversity, second only to habitat loss and degradation and more serious than pollution and overharvesting (e.g., [238]). In San Francisco Bay, AIS dominate many important habitats in number of species, population size, and biomass [239]. Researchers concluded that San Francisco Bay is one of the most invaded estuaries in the world; a new species arrives and becomes established every 14 weeks [239]. In the Northwest, concerns have grown regarding AIS capacity to undermine shellfish harvests. Species such as the invasive tunicates *Didemnum* and *Styela clava*, European green crab, Japanese oyster drill, *Spartina* (cordgrasses), and various pathogens and parasites represent ongoing threats to the regional aquaculture industry (http://wdfw.wa.gov/fish/ans/index.htm; http://www.sfei.org/bioinvasions/BioInvproginfo.htm).

Another emerging West Coast concern is hypoxia, or low oxygen conditions, that may be caused by numerous factors. In urban estuaries, hypoxic events are attributed, at least in part, to excess anthropogenic nutrient input [240]. The duration and severity of the event may be determined by additional factors, such as water depth, wind, and flushing rates [240]. By contrast, offshore events that reduce or eliminate populations of fish and benthic invertebrates in historically productive habitats may be responses to other processes. Although seasonal wind-driven upwelling is known to transport nutrients and low-oxygen water to coastal waters, it is not yet fully understood why ocean events occur some years and not others. Reports of long-term decreases in oxygen concentrations at open ocean and coastal locations have prompted concern about the consequences for marine ecosystems [241]. In August 2006, an event with severe hypoxic and anoxic conditions on the central Oregon coast led to the complete absence of all fish from normally populated rocky reefs and to high mortality of large benthic invertebrates [242].

#### The Census of Marine Life contribution to the California Current region

In 2002, 2003, and 2005, the Census and Scripps Institution of Oceanography's Center for Marine Biodiversity and Conservation sponsored three workshops to examine marine biodiversity in the past, present, and future. In addition, two of the initial field projects of the Census have been focused on the West Coast. TOPP attaches satellite tags to 22 species of top marine predators to study migration patterns and the oceanographic factors that influence them. POST is a tool for tracking the movement of marine animals along the coast, using acoustic transmitters implanted in a variety of species and a series of receivers running in lines across the ocean floor. One major POST focus is the development of a permanent continental-scale marine telemetry system.

### Alaska's Large Marine Ecosystems – the Gulf of Alaska, Eastern Bering Sea and Aleutian Islands, and Chukchi and Beaufort Seas

#### Description of the Alaska region

Alaska's marine waters are some of the most productive in the world; Alaskan commercial fisheries yield over half of fish landings from U.S. waters. Because this is a large region, various ways have been proposed for dividing it ecologically, from a few large ecosystems to many smaller ecoregions (e.g., [243], [244], [245]). For purposes of this overview, the region includes at least parts of four LMEs listed in the Introduction, with emphasis on areas within the U.S. EEZ. The LMEs differ in ecosystem structure, function and biodiversity, in commercial, recreational, and subsistence uses, and in resource management issues [246], as well as in climate, seasonal weather patterns, and sea ice conditions. All are graced with relatively pristine waters, and all have significant deep ocean basin waters.

**Figure 10:**
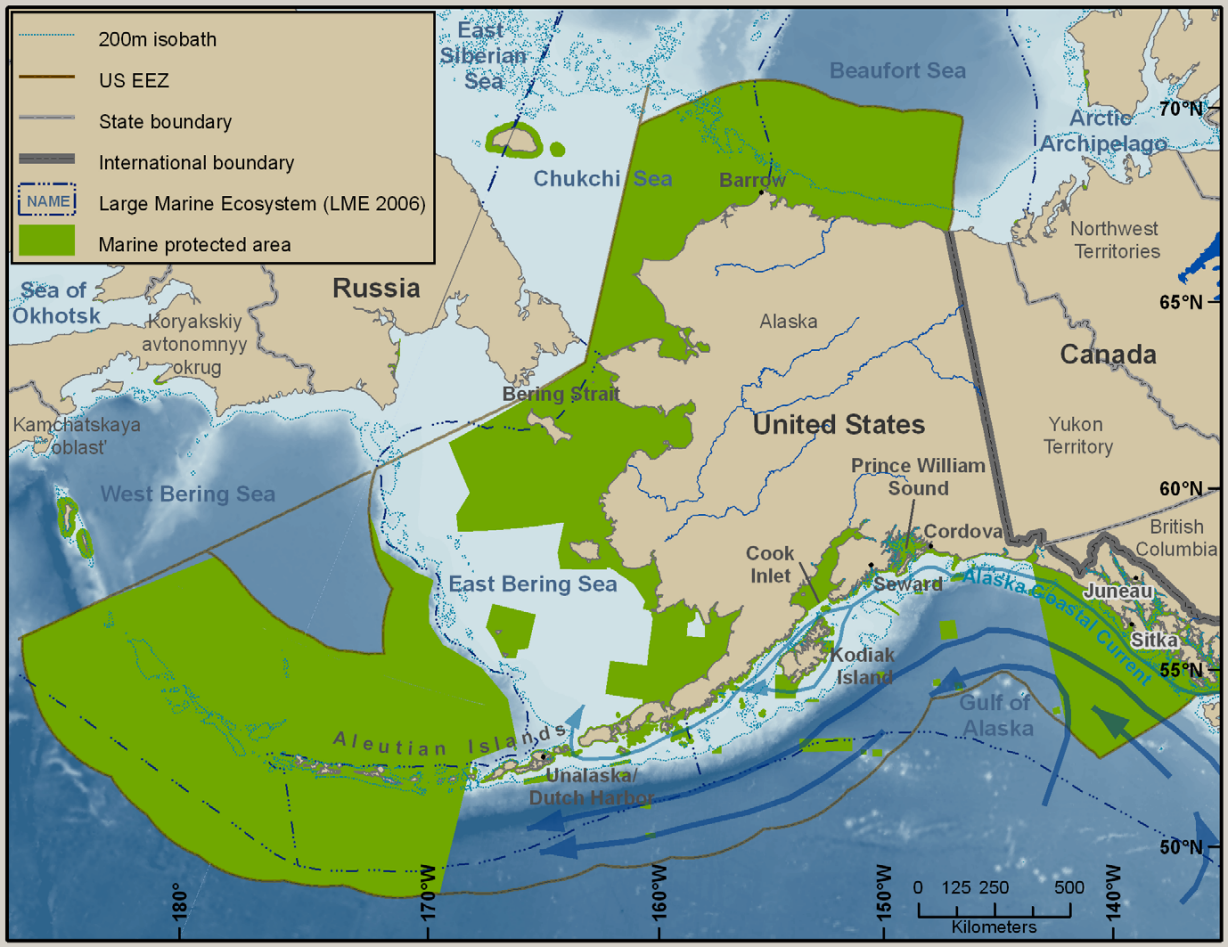
Gulf of Alaska, Bering Sea and Aleutians, and Chukchi and Beaufort seas. Map also shows large marine ecosystems, EEZ boundaries, state boundaries, international boundaries, marine ecoregions, and marine protected areas.

The Gulf of Alaska (GoA) LME ranges from Vancouver Island, B.C. (about 50°N and 125°W) to Samalga Pass in the Aleutians, at roughly 52°N and 169°W (Figure 10). Several significant bodies of water adjoin the Gulf, including Southeast Alaska inside waters, Prince William Sound, and Cook Inlet. The continental shelf is relatively narrow off Southeast Alaska, broadens around Kodiak Island, and then narrows toward the Aleutians. This relatively deep shelf is extremely irregular, reflecting tectonic and glacial influences. A nearly continuous coastal mountain barrier results in enhanced winds and precipitation [247], [248].

The Bering Sea and Aleutian Islands (BSAI) LME extends northward from the Aleutians to the Bering Strait. It has a broad, highly productive continental shelf that supports robust cod, pollock, flatfish, and crab fisheries. Several large underwater canyons cut into the shelf, bringing nutrient-laden waters onto the shelf and providing hard substrate areas of high biodiversity for corals and sponges [249], [250]. The waters are generally well mixed by strong tides and winter winds, although transitional zones or fronts separate the southeastern Bering Sea shelf into coastal, middle, and outer shelf domains in the spring and summer [251], [252].

The high Arctic comprises the Alaska portions of the Chukchi and Beaufort sea LMEs. The boundary between the Arctic and BSAI LMEs is indistinct, and in many respects the hydrographic characteristics of the northern Bering Sea relate more to the Arctic Chukchi Sea than to the southern Bering Sea shelf [253]. These continental shelves are among the largest in the world [253]. Because those of the northern Bering and southern Chukchi Seas are typically less than 50 m deep, they formed a continuous land bridge from Alaska to Siberia during the last ice age, which ended about 10,000 to14,000 years ago [254]. The Alaska Beaufort Sea shelf is relatively narrow.

Coastal currents thread the LMEs together and the presence or absence of seasonal and permanent sea ice helps differentiate them. The Alaska Coastal Current influences all regions, forced mainly by a combination of coastal, wind-driven convergence, and freshwater runoff from land [247]. The Alaska Coastal Current flows northward from British Columbia along the entire Gulf of Alaska coast through Unimak Pass, and onto the southern Bering Sea shelf. There it is low in nutrients because of the strong freshwater and terrestrial influences along its path that extends all the way to the western Beaufort Sea [255]. Upwelling in the Gulf of Anadyr transports nutrient-laden water onto the northern Bering Sea shelf, and the Chirikov Basin, whence it enters the Chukchi Sea through the western portion of Bering Strait [253], [256]. This nutrient transport system drives high primary and secondary production along the way, supporting abundant and diverse marine mammal, bird, fish, and shellfish populations. The Chukchi shelf is influenced on the Russian side by the Siberian Coastal Current flowing east along the northern coast of Russia from the Laptev Sea, bringing considerable river discharge and ice melt. The Alaska side is influenced by freshwater from the Yukon and Kuskokwim rivers transported north by the Alaska Coastal Current.

Seasonal and multiyear sea ice distinguishes the BSAI and Arctic. Sea ice provides a seasonal or year-round substrate for primary and secondary production inside and below the ice [251], [257]. Seasonal ice begins to form on the leeward side of coastlines in late fall, and frigid northerly winds blow the ice southward. The timing of ice advance and retreat and the extent of coverage vary greatly from year to year. The maximum southern sea ice boundary in the Bering Sea in March is predicted to move north over the next 50 years because of global warming. In the Chukchi and Beaufort seas, seasonal ice begins to add to the polar pack ice in early October. By late October or early November, it extends far south through the Bering Strait, joining the ice forming in the Bering Sea. Ice begins to melt in mid-June in the southern Chukchi Sea. The Arctic coastal regions of the Chukchi and Beaufort Seas generally are covered with shore-fast ice for about eight months, but over the past two decades, sea ice extent and thickness have diminished. Record seasonal retreats of sea ice northward into the Chukchi and Beaufort seas were reported for 2002-2005 [258], but they were exceeded by ice retreats in 2007 and 2008 [255]. During the first half of July 2009, Arctic sea ice extent declined more quickly than in 2008, but not as fast as in 2007 (National Snow and Ice Data Center: http://nsidc.org/arcticseaicenews/). Some climate-ocean models predict the ice cover over the Arctic Ocean will decline in extent by roughly 10-50% by 2100, and in summer will completely disappear by 2040 [259]. By 2090, average annual air temperatures are projected to rise across the entire Arctic region by roughly 3 to 5°C over land areas and up to 7°C over the oceans.

The Gulf of Alaska shelf is dominated by gravel, sand, silt, and mud, punctuated by areas of hardrock. There are numerous banks and reefs with coarse, rocky bottoms, but much of the shelf is covered by glacial silt from the Copper River and the Bering and Malaspina glaciers [247]. In contrast, the Aleutian shelf is narrow, with a complicated mixture of substrata, including a significant proportion of hard pebble, boulder, and rock. The Aleutian passes are very deep and have bedrock outcrops and coarsely fragmented sediment interspersed with sand.

The Bering Sea shelf is composed mainly of sand and mud [243]. Small amounts of gravel are common around the shallow eastern and southern perimeter of the shelf, near the Pribilof Islands and in the Bering Strait area. Undersea canyons on the outer shelf with hard substratum are particularly rich areas of marine epifaunal biodiversity. Near St. Matthew and St. Lawrence islands, mud and sand substrata predominate, turning to a complex mixture of substrata north of St. Lawrence Island and into Norton Sound, supporting a rich infaunal community.

The offshore Chukchi Sea is composed of silty sand and mud, whereas the nearshore eastern Chukchi Sea is composed of more heterogeneous and coarser sediments, including sand and gravel. The coastal region is also influenced by river sediments from the Yukon and other Alaska rivers. The Beaufort Sea is blanketed mainly with silty sands and mud from many rivers, especially the MacKenzie River west to the Kuparuk and Colville rivers (e.g., [260]). The Boulder Patch off Prudhoe Bay on the North Slope of the high Arctic with cobbles and boulders that support many species of algae and invertebrates unknown from the surrounding soft-bottom areas [261]. It is protected from deep-draft sea ice by a chain of offshore barrier islands and shoals. The multiyear sea ice provides unique habitat for ice-associated phytoplankton, zooplankton, small invertebrates, and a few species of fish, as well as resting, breeding, and hunting platforms for marine mammals of several species [262].

#### Regional history of biodiversity studies

Marine research off Alaska is not for the faint of heart. High winds and waves, and freezing sea spray often preclude fieldwork during the long winters from October through April or May, and regions of the Bering Sea and Arctic Ocean covered by sea ice require special logistics. These include ice breakers or at least ice-strengthened hulls on research vessels, or in other cases, remote camps on multiyear pack ice. The Gulf of Alaska is the most accessible LME. It has winter storms, and sometimes extensive broken sea ice in Cook Inlet. Research facilities affiliated with the University of Alaska Fairbanks (UAF) or NOAA are located in coastal communities, including Sitka (UAF), Juneau (UAF and NOAA), Cordova (Prince William Sound Science Center), Seward (UAF and Alaska SeaLife Center), Kachemak Bay (NOAA and UAF), and Kodiak (NOAA and UAF). In the Bering Sea and high Arctic, it is likely that most large-scale federally funded research programs will continue to focus on support of fisheries and oil and gas activities. Research there requires ships capable of operating in high seas and storm conditions. These large vessels, such as the *Healy*, *Miller Freeman*, and *Oscar Dyson*, typically load and offload in Seattle, Kodiak, and Dutch Harbor. Transit times are long and operations expensive. This also applies to Arctic operations, which will require icebreaker support for the foreseeable future [263]. Establishing and maintaining camps on the pack ice are even more expensive and time consuming.

This region has many, diverse Native communities with intimate cultural connection to the marine ecosystems that sustained them for thousands of years. A wealth of traditional ecological knowledge is held in these communities, but is only just beginning to be incorporated into scientific understanding of Alaska's marine ecosystems [246].

There have been many scientific expeditions over the years, despite the high cost and adverse conditions. PICES funded an illustrated historical review of seafaring discovery and scientific exploration of the North Pacific Ocean from 1500 to 2000 [223]. Voyages of early explorers were mostly for mapping. Vitus Bering's Second Kamchatka Expedition (1741-42) included Georg Steller, the first scientist to be carried on a Pacific voyage, who identified five species of salmon, named various seabirds and marine mammals, and described life cycles and ecological relationships. In the 1820s, Russian voyages associated with the Russian-American Company carried naturalists who collected fishes, crustaceans, and birds that apparently were deposited in the Imperial Russian Academy of Sciences in St. Petersburg, Russia. In 1874, deep benthic species were collected in the Aleutian Trench on an expedition led by George Belknap on the U.S. steamer *Tuscarora*. In 1886, the U.S. Fisheries Commission sent its flagship, the *Albatross*, to work Alaskan waters each summer, concentrating on the species harvested for subsistence and commercial use. In 1899, the Harriman Alaska Expedition explored the Pacific coastal waters from Seattle through Prince William Sound, out to the Aleutians and north along the Russian coast of the Bering Sea [264]. Invertebrates were surveyed extensively and specimens were distributed to universities and museums for analysis and identification, resulting in four volumes on species ranging from ribbon and segmented worms to sea stars and sea spiders [265], [266], [267].

In 1955, the NORPAC (North Pacific) Expeditions used 19 ships from 14 oceanographic institutions in three countries to collect near-simultaneous ocean data across the North Pacific between 20 and 60°N. Phytoplankton and zooplankton composition and abundance data were collected along with oceanographic data. The Japanese research vessel *Oshoro Maru* and the University of Washington's *Brown Bear* were primarily responsible for data collection off Alaska. The data were published in the *NORPAC Data* and *NORPAC Atlas* of the annual publication *Oceanographic Observations of the Pacific*. Starting in the 1950s, the Russian research vessel *Vityaz*, the Bering Sea Commercial Research Expedition, and the Pacific Research Institute of Fisheries and Oceanography (TINRO), as well as the *Oshoro Maru*, collected zooplankton and deep-sea fauna in the North Pacific [268]. A review of investigations in the Gulf of Alaska is provided by Hood [269] and reviews of mostly benthic investigations in the Bering, Chukchi, and Beaufort seas are provided by Grebmeier et al. and by Sirenko and Gagaev [253], [270].

The early expeditions in the Aleutians and Gulf of Alaska inventoried marine biodiversity qualitatively rather than quantitatively, but the most comprehensive and accessible programs are more recent ones funded by MMS and NOAA. These programs originally aimed at providing baselines for oil and gas development. Fish stock assessments became mandatory in the 1980s as foreign fisheries were replaced by U.S. fisheries. The MMS Outer Continental Shelf Environmental Assessment Program (OCSEAP), which began in 1974 and continues today, although at a lower level since the mid-1980s, has collected extensive species information. NOAA's bottom trawl surveys collect information on fishes and many species from the Bering Sea and Aleutians and Gulf of Alaska to support fishery management decisions by the North Pacific Fishery Management Council and the Secretary of Commerce. Biodiversity information has also been collected by the *Exxon Valdez* Oil Spill Trustee Council in Prince William Sound, continuous plankton recorder surveys across the North Pacific, Seward Line zooplankton collections in the Gulf of Alaska, and Hokkaido University's annual training cruises on the *Oshoro Maru* to the Bering Sea and Strait and, less frequently, to the Chukchi Sea.

Biodiversity research in the high Arctic Ocean is described by Hopcroft et al. [271]. Pack ice provided a platform for sampling from stations such as T-3 and Arctic Ice Dynamics Joint Experiment in the 1960s and 1970s, and extensive Arctic nearshore research was funded by MMS through OCSEAP in the 1970s and 1980s. Among numerous expeditions by icebreakers and other research vessels to the northern Bering Sea and Strait, the Chukchi Sea, and other Arctic regions [272] is the Western Arctic Shelf-Basin Interactions Project in 2002-2008, which assessed the effects of variability in sea ice cover and hydrography on the marine ecosystem and the impacts of climate change. The project included extensive collection of specimens and mapping of benthic fauna [255], [273].

The NOAA Office of Ocean Exploration supported cruises to study biodiversity in 2002 and 2005. An international team of 50 scientists from the U.S., Canada, China, and Japan used a remotely operated vehicle specially designed to operate under ice and at great depth to explore biota over the full range of habitats in the deep Canada Basin, from brine channels in the sea ice to the benthic communities. Among other activities, the program sampled under-ice fauna and gelatinous zooplankton, and collected cephalopods and deep-sea benthic fauna. The Russian-American Long-term Census of the Arctic in 2004-12 involves a partnership with the Russian Academy of Sciences and other Russian institutions to create a benchmark dataset to study the distribution and migration patterns of organisms in the Pacific gateway area of the Bering Strait and southern Chukchi Sea, regions that are thought to be particularly sensitive to climate change [270]. Three biodiversity-focused programs supported under the Census are ArcOD [274], NaGISA [275], and the Census of Marine Zooplankton (CMarZ) [276], [277]. Recently, MMS initiated programs in Arctic waters because of renewed national interest in oil and gas exploration and development. For example, in August 2008, NOAA's Alaska Fisheries Science Center was funded by MMS to survey the offshore waters of the Beaufort Sea to provide estimates of abundance and species composition of marine fishes and invertebrates, as well as information on the macro- and microzooplankton communities and their oceanographic environment [278]. Also in 2008, the oil and gas industry began new biological assessment programs (Chukchi Sea Offshore Monitoring in Drilling Area: Chemical and Benthos) in the Chukchi Sea in response to the sale of leases for new offshore prospect areas.

Understanding ecosystem processes and relationships of organisms within ecosystems is a focus of large-scale research programs off Alaska (e.g., see the joint National Science Foundation–North Pacific Research Board Bering Sea study at http://bsierp.nprb.org). In addition to such hypothesis-driven ecosystem research are significant efforts to support resource management by monitoring fish and invertebrate distribution and abundance through time. These surveys contribute to broader knowledge of biodiversity, continuing to add to the many efforts over the past 40 years to enumerate species from the coastal rocky headlands to the deep ocean and even in sea ice. However, no species inventory exists of all realms for any region of Alaska. A census of all organisms, including plants, will require intensive discovery and compilation (and in some cases, translation) of a wide variety of taxonomic works for the North Pacific, including those from Japan and Russia, two countries that have done significant research in northern waters for decades. For example, Sirenko [279] provides the most extensive taxon lists available for free-living invertebrates in the high Arctic. ArcOD is working under the leadership of Dr. Sirenko in St. Petersburg, Russia, on a multi-volume taxonomic inventory for the Arctic that eventually will be published by Alaska Sea Grant. The first volume treated mostly groups of free-living crustaceans [280]. A special issue in *Deep-Sea Research Part II* includes papers on sea ice, pelagic and Benthic communities, food web structure, and barcoding, with a focus on the Chukchi Sea and Canada Basin [281].

Significant databases containing biodiversity information are listed in Table S4. What data from federal programs sponsored by MMS and NOAA are available at the National Ocean Data Center (NODC) is unclear because data and metadata submission requirements are unevenly enforced by federal agencies. Many individual project reports from OCSEAP are available online at the Alaska Resources Library and Information Services (ARLIS: http://www.arlis.org/docs/vol1/OCSEAP2/macro.html), but it is unclear whether all datasets in those reports were digitized and submitted to NODC.

Electronic project reports for research funded by the Exxon Valdez Oil Spill Trustee Council (EVOSTC) on benthic nearshore biodiversity in Prince William Sound and along the North Gulf coast are available at http://www.evostc.state.ak.us/, but most EVOSTC funds were passed to agencies, such as the Alaska Department of Fish and Game, to do the work, and now, years later, EVOSTC is just beginning to compile the project data for a central database. There seems also to be a large dataset at the Institute for Marine Science at UAF containing data on a broad range of species collected along the Alaska shoreline just after the 1989 oil spill, at a cost of some $20 million, but the extent to which these data are being rescued and made accessible is unclear.

#### The known, unknown, and future directions

Surveys and monitoring, such as described above, must continue to document how the species mix may change as organisms migrate into and out of Alaska waters because of global change. Although currently there is no comprehensive list of species for all of Alaska because either there are no Alaskan surveys of particular taxa or the literature is inadequate for compiling such an all-inclusive list. However, comprehensive lists exist for particular regions. Bodil Bluhm, Rolf Gradinger, and Russ Hopcroft, who are associated with ArcOD, have compiled the most complete list available for any region of Alaska, an inventory of nearly 6,000 species for the Arctic Ocean (Table 10; more detail is available in Table S1) that is based heavily on Sirenko [279]. More conservative lists for each LME have been provided by Bruce Wing (Curator, Reference Collections, at the Auke Bay Lab of NOAA's Alaska Fisheries Science Center), estimating 542 species in the Gulf of Alaska, 572 in the Bering Sea and Aleutian Islands, and 220 in the Arctic (Table S7), with roughly 2,500 species Alaska-wide. The total number is smaller than for just the Arctic (albeit the entire Arctic) cited above, which would imply the total number of species for Alaska as a whole is at least several times and possibly an order of magnitude greater than that given in Table S7. The large discrepancies in various estimates stem from, and are evidence of, the incomplete knowledge of Alaskan marine biodiversity. We will not know how many species exist off Alaska until a major effort is made to combine and reconcile species lists and then “ground-truth,” or verify, identifications using accurate, scientifically acceptable taxonomic guides. Until then, investigators interested in marine biodiversity will need to rely on primary research articles and summaries for specific assemblages and subregions. See Text S2 for a list of useful taxonomic guides. In addition to taxonomic studies, knowledge of marine habitats off Alaska and habitat mapping are needed.

**Table 10:** Biotic diversity in the high Arctic (not exclusively the U.S.).

Taxonomic group	No. species^1,2^
**Domain Archaea**	**UD**
**Domain Bacteria (including Cyanobacteria)**	**UD**
**Domain Eukarya**	**5,925**
**Kingdom Chromista**	**287**
Phaeophyta^3^	
**Kingdom Plantae** 3	**150**
Chlorophyta	
Rhodophyta	
Angiospermae	
**Kingdom Protoctista (Protozoa)**	**759**
Dinomastigota (Dinoflagellata)	70
Foraminifera	325
**Kingdom Animalia**	**4,729**
Porifera	163
Cnidaria	227
Platyhelminthes	134
Mollusca	488
Annelida	533
Crustacea	1525
Bryozoa	331
Echinodermata	151
Urochordata (Tunicata)	64
Other invertebrates	600
Vertebrata (Pisces)	415
Other vertebrates	98
**TOTAL REGIONAL DIVERSITY** 4	**5,925**

(Bluhm, Gradinger and Hopcroft, personal communication).

**Notes:**

1 Sources of the reports: databases, scientific literature, books, field guides, technical reports, and personal communication with taxonomic experts.

2 Identification guides cited in Text S2.

3 The 150 species of seaweeds listed by Bluhm, Gradinger and Hopcroft for the entire Arctic include Phaeophyta. For more detail regarding seaweeds in the Arctic Ocean, see Table S1.

4 Total regional diversity for the Arctic Ocean, including all taxonomic groups, is reported in Table S1.

UD  =  Listed in work but number undetermined to date.

Atlas and Griffiths [282] reviewed studies of bacteria in the Gulf of Alaska, identifying 13 genera of isolates: Microcyclus, Moraxella, Acinetobacter, Vibrio, Beneckea, Aerimonas, Flavobacterium, Alcaligenes, Arthrobacter, Bacillus, Pseudomonas, Chromobacterium, and Micrococcus. In the Arctic Ocean, Bano and Hollibaugh [283] identified approximately 18 phylotypes, which they consider a minimum estimate of richness. They concluded that Arctic Ocean bacterioplankton assemblages, which are as complex as assemblages from California coastal waters, represent novel groups of organisms, at least compared with those from tropical and temperate waters.

Photographs and descriptions of the more abundant and visible species of seaweeds are at http://www.seaweedsofalaska.com: they include 23 species of Chlorophyta (green), 37 of Phaeophyta (brown), and 61 of Rhodophyta (red). This is only a partial list of Alaskan seaweeds. A recent survey of specimens collected over the past two centuries, together with the application of molecular techniques to recent collections, shows surprisingly high diversity, given the history of glaciation, large areas of unsuitable habitat, and otherwise harsh environmental conditions [284]. The number of recognized species has increased from 376 in 1977 to about 550 today, and may actually be around 600 species, with recent discoveries of previously unknown seaweeds in the Aleutians (e.g., a new species of kelp on Kagamil Island [285]) and the northern Gulf of Alaska (S. Lindstrom, personal communication). There is a wide range of biogeographic patterns: species that occur primarily to the south and have their northern limit in Alaska, species that occur primarily to the west and have their eastern limit in Alaska, species that are primarily Atlantic but extend through the Arctic to Alaska, and some endemics. Southeast Alaska alone has on the order of 368 species of seaweeds, making it the most diverse region in the state [284], [286]. The entire Arctic is estimated to have about 150 species [287] of which about half are verified to occur in Alaska (S. Lindstrom, personal communication). The Alaska Seaweed Database (http://herbarium.botany.ubc.ca/herbarium_data/algae_alaska/search.htm) lists 25 Phaeophyceae species, 18 Rhodophyta, 15 Chorophyta, and 1 Plantae species (Table S1).

One of many lists of planktonic species is that by Sambrotto and Lorenzen [288] for phytoplankton in the eastern subarctic Pacific north of 42°N and east of 180°W, which includes the Gulf of Alaska and much of the Aleutian Islands area. Cooney [289] provided a list of zooplankton for the northern Gulf of Alaska. R. Hopcroft (personal communication) compiled a list for the Arctic Ocean, which he noted was continually evolving and will be included in the more formal Arctic Register of Marine Species now under development. Combined, as shown in Table 11, roughly 350 species of phyto- and zoo-plankton occur in the Gulf of Alaska. The most speciose group is Crustacea, which includes an abundance of copepods, amphipods, decapods, and mysids. Crustaceans are also diverse in the Bering Sea and the Gulf of Alaska. Cooney [290] listed 310 species of zooplankton in the Bering Sea. Motoda and Minoda [291] provided a list of 327 species (including phytoplankton) for the Bering Sea. For gelatinous zooplankton of the Arctic Ocean, Hopcroft [292] listed 6 ctenophores, 45 medusae, 12 siphonophores, 4 pteropods, and 5 larvaceans, but noted that twice as many species will be identified when sampling is completed. Kosobokova and Hopcroft [293] listed a total of 111 species, including 74 species of crustaceans (55 copepods, 2 euphausiids, 11 amphipods, 1 decapod, 5 ostracods), 17 cnidarians, 1 foraminiferan, 4 ctenophores, 2 pteropods, 4 larvaceans, 4 chaetognaths, and 5 polychaetes from a single Canada Basin cruise. For the Arctic Ocean in general, Horner [294] estimated there are some 287 diatom species. In a single sea ice core taken in the Chukchi Sea, Quillfeldt et al. [295] found 237 diatom species. Hopcroft's estimate of 357 plankton species in the Arctic is based on his unpublished data added to species lists provided by Sirenko [279].

**Table 11:** Plankton species estimates for Gulf of Alaska, Bering Sea and the Arctic Ocean (numbers should be considered minimum estimates).

Taxa	Gulf of Alaska plankton^a^	Bering Sea plankton^b^	Bering Sea zooplankton^c^	Arctic Ocean^d^
Radiolaria	116	24		40
Chlorophyta		5		
Foraminifera		111		3
Dinoflagellates	14	16		
Cnidaria	42	21	41	73
Ctenophora	3	1	1	13
Annelida	9	1	17	6
Crustacea	152	139	235	203
Mollusca	9	1	8	5
Chaetognatha	5	6	6	10
Urochordata	4	2	2	4
**Total**	**354**	**327**	**310**	**357**

**Notes:**

a Sambrotto RN, Lorenzen CJ (1986) Chapter 9. Phytoplankton and primary production. In: Hood D, Zimmerman S, editors. Gulf of Alaska, physical environment and biological resources. Washington, D.C.: NOAA Ocean Assessments Division, Alaska Office. pp. 249-282. Cooney RT (1986) Chapter 10. Zooplankton. In: Hood D, Zimmerman S, editors. Gulf of Alaska, physical environment and biological resources. Washington, D.C.: NOAA Ocean Assessments Division, Alaska Office. pp. 285-303.

b Motoda S, Minoda T (1974) Plankton of the Bering Sea. In: Hood DW, Kelley EJ, editors. Oceanography of the Bering Sea with emphasis on renewable resources: Institute of Marine Sciences, University of Alaska Fairbanks. pp. 207-241.

c Cooney RT (1981) Bering Sea zooplankton and micronekton communities with emphasis on annual production. In: Hood DW, Calder JA, editors. The eastern Bering Sea shelf: Oceanography and resources Vol. 2. Seattle: University of Washington Press. pp. 947-974.

d Sirenko BI (2001) List of species of free-living invertebrates of Eurasian Arctic seas and adjacent deep waters. Explorations of the Fauna of the Seas 51(59). (Plus unpublished data from Russ Hopcroft).

As with plankton, there are many lists of benthic invertebrate species (e.g., [261], [279], [296], [297], [298], [299], [300], [301]). Some examples are given in Table 12. The detailed study of macrofauna in Prince William Sound by Foster [299] provided one of the most extensive lists, with a total of 1,582 species, most of which were crustaceans, mollusks, annelids, and cnidarians. To the north, Stoker [296] found fewer macrofaunal species in the eastern Bering Sea and Chukchi Sea shelf than in the Gulf of Alaska: most of the 492 species were mollusks, annelids, and crustaceans. In the Boulder Patch, Dunton and Schonberg [261] found 204 species, with annelids, mollusks, and crustaceans predominating. To the east, in the southern Beaufort Sea and west Amundsen Gulf, Chapman and Kostylev [302] compiled a list of 855 benthic species, dominated by annelids, crustaceans, and mollusks.

**Table 12:** Invertebrate taxa in various regions off Alaska.

Taxa	Prince William Sound macro-fauna^a^	Eastern Bering & Chukchi Seas shelf macro-fauna^b^	Arctic Boulder Patch epi-benthos^c^	Chukchi Sea free-living invertebrates^d^	Central Arctic Basin free-living invertebrates d	Beaufort Sea & W. Amundsen Gulf benthic invertebrates e
Ciliophora				4	1	
Radiolaria				13	11	
Foraminifera				61	191	18
Porifera	12	1	6	18	27	5
Cnidaria	106	9	15	73	64	44
Ctenophora	5			4		
Platyhelminthes				1		1
Nemertea	59			13	2	1
Kinorhyncha				5		1
Priapulida	1	1	1			5
Sipuncula	4	3		7	6	3
Echiura	1	1		2		
Annelida	301	147	59	185	73	230
Pogonophora					6	1
Crustacea	554	143	46	414	379	291
Chelicerata (non-arachinid)				14		13
Mollusca	340	143	49	185	32	155
Bryozoa/Ectoprocta	82		14	109	1	28
Brachiopoda	5	2		2	4	2
Echinodermata	72	32		33	26	38
Chaetognatha	5		1	11	10	
Urochordata	35	10	4	14		19
**Total**	**1,582**	**492**	**195**	**1,168**	**833**	**855**

**Notes:**

a Foster NR (2003) Database on the marine invertebrate macrofauna of Prince William Sound: An addition to the University of Alaska Museum's ARCTOS Network. Exxon Valdez Oil Spill Gulf of Alaska Monitoring and Research Project 030642 Final Report.

b Stoker SW (1978) Benthic invertebrate macrofauna of the eastern continental shelf of the Bering and Chukchi Seas. Ph.D. Thesis: University of Alaska Fairbanks. 259 p.

c Dunton KH, Schonberg SV (2000) The benthic faunal assemblage of the Boulder Patch kelp community. In: Truett JC, Johnson SR, editors. The natural history of an arctic oil field. New York: Academic Press. pp. 371-397.

d Sirenko BI (2001) List of species of free-living invertebrates of Eurasian Arctic seas and adjacent deep waters. Explorations of the Fauna of the Seas 51(59).

e Chapman AS, Kostylev VE (2008) Distribution, abundance and diversity of benthic species from the Beaufort Sea and western Amundsen Gulf – a summary of data collected between 1951 and 2000. Geological Survey of Canada. Open File 5685. 47 p.

In a study of over 14,000 stations in Arctic seas, Sirenko and Piepenburg [303] found 4,296 animal species, about 87% invertebrates. Benthic species predominated, the richest group being crustaceans (1,075 species, or 25%). They noted that in general, benthic taxa are not as well studied as planktonic taxa, and that among the benthic taxa, macrobenthic groups tend to be best known. They estimated that about 90% of species of foraminiferans, sponges, bryozoans, mollusks, and echinoderms are known, whereas turbellarians, nematodes, scyphomedusae, ascidians, and ostracods are particularly poorly studied. They concluded that an estimated 1,800 invertebrate species remain unknown, which, when added to the known species, would result in an estimate of about 5,600 invertebrate species in Arctic seas. Expanding on that study, Sirenko [279] provided a list of free-living invertebrates, 1,168 species from the Chukchi Sea (updated to 1,436 species in 2009 [304]) and 833 species from the central Arctic Ocean Basin, for a total of 4,784 from the entire Arctic. Sirenko included both planktonic (about 300 multicellular species, which comprise 6% of Arctic species) and benthic species (macrobenthos are 60% of species, and meiobenthos are 34%). As with other lists, crustaceans, mollusks, and annelids contributed high numbers of species, and more species remain to be found. For example, after discovering 9-10 new invertebrate species in only about 2 m^2^ of seafloor in the Canada Basin, MacDonald et al. [305] concluded that potentially hundreds of more new species may be found in future inventories in the Arctic basins or even in the Canada Basin alone.

The most recent and comprehensive compilation of fish species off Alaska is by Mecklenburg et al. [306], with updated georeferenced species information in the authors' database at http://www.arcodiv.org/Database/Fish_datasets.html (linked to OBIS). They reported 521 confirmed species Alaska-wide, plus another 80 reported but not confirmed, or probably in Alaska but not reported. The 521 species include 474 saltwater species, 22 freshwater species, and 25 that are anadromous or euryhaline. Sculpin and rockfish dominate the list. There are roughly 341 species in the Gulf of Alaska, 367 in the Bering Sea and Aleutians and 78 in the Arctic. These contrast with earlier estimates of 287 species belonging to 55 families in the Gulf of Alaska [307], and 300 in the Bering Sea [308]. From NOAA bottom trawl surveys along the Aleutians, Logerwell et al. [309] found 245 fishes in three provinces (Arctic-Kurile, Kurile, and Oregonian). The most diverse assemblage, composed mainly of arrowtooth flounder, Pacific cod, Pacific halibut, rock soles, and walleye pollock, was in the northeast shallow continental shelf of the Aleutians, east of Adak Strait. In a synthesis of information on the Arctic Ocean, Mecklenburg et al. [310] reported up to 104 fish species. Mecklenburg et al. [306] noted that their catalog added a minimum of 90 new confirmed species to the inventory done in 1972. Andriashev and Chernova [311] list 415 species of marine, diadromous, and freshwater species occurring in brackish waters for the entire Arctic and adjacent waters.

Twenty-six species of marine mammals, which include seals, sea lions, walrus, whales, dolphins, porpoises, sea otters, and polar bears, exist off Alaska; 25 are documented by Angliss and Allen [312] and the narwhal was added recently by NOAA. Seventeen of those species occur in the Gulf of Alaska, 25 in the Bering Sea, and 14 in the Arctic (J. Ferdinand, NOAA, personal communication).

Alaska seabirds are represented mainly by albatrosses, shearwaters, fulmars, storm petrels, cormorants, gulls, puffins, murres, auklets, and murrelets. Other bird groups using marine waters, such as loons, grebes, phalaropes, and sea ducks technically are not “seabirds”. Some 38 seabird species breed in Alaska and up to about 33 additional species return regularly to Alaska to feed, but breed elsewhere (http://alaska.fws.gov/mbsp/mbm/seabirds/species_list.htm). Fifteen species of sea ducks inhabit Alaska waters (http://seaduckjv.org/meetseaduck/toc.html). Hunt et al. [313] estimated that about 45 species of seabirds occur regularly in the Bering Sea and Aleutians. Gill and Handel [314] estimated that about 52 species of shorebirds frequent the Bering Sea and Aleutian coastal areas. About 26 species of seabirds nest around the rim of the Gulf of Alaska [248], and Springer et al. [315] estimated that 34 species of seabirds nest in the U.S. part of the western Arctic and that an additional 6 migrate to it during summer to feed on locally abundant prey. This estimate of about 40 species of seabirds that nest in, or visit, the Arctic contrasts with the 82 species estimated by Bluhm, Gradinger and Hopcroft (personal communication) for the entire Arctic (Table 10).

Marine turtles are casual visitors to Alaska waters and are not necessarily just carried in occasional warm currents [316]. Since 1960, marine turtle occurrences include 19 leatherbacks, 9 greens, 2 Pacific Ridleys, 2 loggerheads, and 2 unidentified hard-shell turtles. Marine turtles were observed in 14 of 39 years from 1960 to 1998; 75% of the occurrences were in July through October.

Species of historical, social, and economic importance are mainly invertebrates and fishes that are fished commercially and at a subsistence level, although marine mammals are increasingly important with latitude, mostly because of their cultural prominence and subsistence use. Salmon and halibut, iconic species in the Gulf of Alaska and Bering Sea, are important to most of the coastal communities in the region and serve as economic drivers in both commercial and recreational fisheries. Other important harvested species in the Gulf and the Bering Sea include pollock, Pacific cod, flatfish, sablefish, rockfishes, herring, scallops, and crabs of various species [317]. Farther north, species of most interest are those important to commercial or subsistence fisheries: Arctic cod, ciscoes and other whitefishes, salmon, and trout. The exact mix of species will change over the coming years if climate warming allows southern species to move north into potentially more ice-free, warming waters.

Marine mammals of cultural and subsistence significance include walruses, northern fur seals, Steller sea lions, sea otters, ice seals (ribbon, ringed, bearded, and spotted), harbor seals, and certain whales, such as bowheads and belugas [246]. Seabirds important to subsistence include the red-faced cormorant, spectacled and Steller's eiders, gulls, kittiwakes, murres and murrelets, and auklets. Species such as albatrosses, fulmars, and petrels affect the economics of the commercial fishing fleet because they are prone to direct strikes and incidental catch. Protection measures may include commercial fisheries closures triggered by the numbers of seabirds taken as bycatch and mandatory deployment of seabird deterrence measures throughout the fishing fleet [246].

Some regions have been studied intensively and stand out for their biotic richness. For example, the 1989 *Exxon Valdez* oil spill stimulated comprehensive baseline studies of nearshore areas in Prince William Sound and downstream from the spill, well past Kodiak [248]. These highly productive, diverse nearshore areas provide nursery habitat for juvenile pink and chum salmon and juvenile herring, and the annual growth of microalgae, seaweeds, and seagrasses in the intertidal and shallow subtidal zones supports many invertebrates, which are prey for fishes, seabirds, and marine mammals. Foster [299] identified nearly 1,600 marine invertebrate species in Prince William Sound, and even that is an underestimate, because not all phyla were included. Prince William Sound's rocky intertidal and shallow subtidal habitats also have been studied by Konar et al. [318] to determine taxon richness, invertebrate abundance, and macroalgal biomass and depth-stratified community zonation patterns. These types of in-depth studies document changes in the Sound as it slowly recovers from the 1989 oil spill.

A second example of high biodiversity is the Aleutian Islands, well known to be globally important for marine birds. In addition, the region may harbor the highest diversity and abundance of cold-water corals in the world [250]. Discoveries of wondrous, possibly unique, communities of corals, sponges, and bryozoans have prompted fisheries closures to protect the diverse species from gear impacts [319]. Submersible observations have documented representatives of six major taxonomic groups and at least 50 species or subspecies of corals that may be endemic to the region. Alaska has about 141 species of corals in Alcyonacea (soft corals), Gorgonacea (sea fans, bamboo corals, and tree corals), Scleractinia (cup corals or stony corals), Stylasterina (hydrocorals), and Antipatharia (black corals). The coral gardens are characterized by a rigid framework, high topographic relief, and high taxonomic diversity. The species mix varies by region; gorgonians and black corals and most common in the Gulf of Alaska, and gorgonians and hydrocorals most common in the Aleutian Islands. Soft corals are common on Bering Sea shelf habitats, and corals are found as far north as the Beaufort Sea.

The biodiversity of the Bering Strait and southern Chukchi Sea has also been well studied [253], [262], [270], [279]. The region has an abundant and diverse macrobenthos of relatively high biomass, dominated by polychaetes, crustaceans, bivalve mollusks, and ophiuroid echinoderms [301]. Epifaunal numbers are dominated by gastropods, abundance by crustaceans, and biomass by echinoderms, mainly sea stars [300]. Most dominant species are of boreal Pacific rather than Arctic origin, owing to the prevailing northward flowing currents.

The Boulder Patch, another well-studied area [261], is one of the richest and most diverse biological communities in the American Beaufort Sea. It contains about 140 taxa of benthic infauna from 11 invertebrate phyla dominated by polychaetes, mollusks, and bryozoans, and an even more diverse assemblage of epifauna, including 158 taxa dominated by fishes, sponges, mollusks, bryozoans, cnidarians, and polychaetes. These communities are important to Arctic nearshore food webs and could be vulnerable to anthropogenic activities that increase siltation or add contaminants. NaGISA has sampled macroalgal-associated habitats in the Boulder Patch and also discovered a new boulder field about 100 km farther west in Camden Bay [320]. Camden Bay has 13 macroalgal species and 58 invertebrate taxa, making it a hot spot for biodiversity along the Beaufort Sea coast.

Sea ice serves as habitat for a unique, highly specialized community of bacteria, algae, protozoans, and metazoans, which contribute to the biogeochemical cycles of polar seas [262]. Inside the sea ice in brine pockets and channels, more than 200 diatom and 70 flagellate species have been identified. Metazoan fauna is thought to be less diverse, although several taxa remain unidentified or have only recently been described [321]. These are grazed by amphipods, an important food source for diving birds and Arctic cod. Arctic cod provide a crucial link between the sea ice food web and marine mammals. ArcOD is a Census project that is providing more understanding of life living in sea ice and brine channels, some of the coldest habitats in the global ocean.

The many deep-sea canyons, such as Pribilof, Bering, and Zemchug canyons, that incise the continental shelf as well as the surrounding shelf break and slope, are features ripe for discovery of new epifaunal species. Also of interest is Bowers Ridge, a submerged structure that forms an arc extending to the north and west from the Aleutian Islands. The top of the ridge rises to less than 200 m from the surface near its southern end, with a deeper area to the north. Relatively unexplored, the ridge is likely to include habitats for corals as well as fishes and crabs.

A greater understanding is needed of the biogeographic patterns of cold-water corals and how they relate to corals elsewhere [250]. This will require more research in the field using multibeam surveys and submersibles to collect specimens and data on distribution, and more laboratory analyses of palaeontology, phylogeny, taxonomy, and genetics. It may be that the endemism and high diversity and abundance of corals in the Aleutians are evidence that that is the center of origin for some taxa [250].

Additional research is needed on the many seamounts and pinnacles in Alaska waters. There have been submersible dives on some pinnacles, such as at Cape Edgecumbe near Sitka. These rise from about 160 m to within 40 m of the ocean surface. The sides and tops of the pinnacles are composed of columnar basalt, and gorgonian corals grow on the steep walls. The boulder fields at the base of the pinnacles provide refuge for adult rockfish, lingcod, and giant Pacific octopus. The top of the pinnacles are covered with anemones, tunicates, and hydrocorals. Adult lingcod aggregate there during the late spring and early summer [319]. The NOAA Office of Ocean Exploration program supported studies of five seamounts, Giacomini, Pratt, Welker, Denson, and Dickens, which stretch over a 750 km section of the northeast Pacific, called the Kodiak-Bowie Seamount Chain. The 2004 Gulf of Alaska Seamount Expedition used the deep submersible vehicle *Alvin* in 2004 to make 17 dives on the seamounts to depths of 3,500 m. It discovered a complex community of organisms, ranging from microscopic to macroscopic, living on corals. The voyage and plan are summarized at http://oceanexplorer.noaa.gov/explorations/04alaska and http://oceanexplorer.noaa.gov/explorations/02alaska/background/plan/plan.html.

The high Arctic also needs more sampling. It is undergoing dramatic change with retreat of the permanent, as well as seasonal, ice. Special techniques will be needed to sample the deep basins where most new species probably will be found. A more complete inventory of benthic species is needed along with estimates of their genetic diversity, and detailed maps of their distribution. ArcOD has begun such research, barcoding 360 Arctic species of benthos, plankton, and fish, many from Alaska [322]. Microbial and meiofaunal communities also are poorly known.

Finally, a cautionary note. Information in existing databases and portals is not necessarily accurate. In the Arctic, for example, unresolved taxonomic questions and controversial classifications plague efforts to evaluate and synthesize information on Arctic species of fish and lower-trophic-level species [271]. Because of limited sampling, all but the most common species are known from small numbers of specimens, and the available specimens and distributional records are inadequate to determine taxonomic and distributional boundaries, especially for similar-looking species (or subspecies) that inhabit the same areas, so are frequently misidentified. Voucher specimens should be deposited to help allay this problem in the future. Name changes due to taxonomic reorganization are inevitable. For example, the taxonomy and phylogenetic relationship of kelps in the GoA have been reevaluated, resulting in the most common kelp species having been transferred from genus *Laminaria* to genus *Saccharina* [323]. One of the more abundant canopy-forming kelps in Alaska has experienced two name changes in about that many years: the species known as *Alaria fistulosa* was changed by Lane et al. [324] to *Druehlia fistulosa*, and then by Wynne (2009) [325] to *Eualaria fistulosa*.

There also should be a concerted effort to retrieve data from Japanese and Russian research cruises in the Bering Sea over the past 50 years, as well as old records from past expeditions such as on Fletcher's Ice Island T-3. Private industry data from oil and gas companies, such as BP and ConocoPhillips, also should be retrieved. NOAA and MMS should be encouraged to submit biodiversity data to OBIS from new research activities in the Beaufort and Chukchi seas.

#### Trouble spots and emerging issues

Global change will be the most significant issue for biodiversity in Alaska in the near future: it can affect diversity within species, between species, and of ecosystems [326]. Warming will alter the geographic distribution of marine organisms (e.g., [270]), the flow of energy within the ecosystem [327], and ecosystem productivity and resilience. Most of the present ice-covered areas are likely to have reduced ice cover, especially in summer, which could lead to increased primary and secondary production and possibly enhanced fish production [328]. Arctic benthic communities of Atlantic and Pacific origin are likely to expand, displacing colder-water species, especially those with narrow temperature preferences. There also will be a shift northward in the distribution of many species of fishes, which could lead to extinction of some current Arctic species.

In the Bering Sea and Arctic Ocean, sea ice is predicted to decline significantly, a process that has already begun (e.g., [258]). There probably will always be seasonal ice, even in the northern Bering Sea, but multiyear ice will decrease, disappearing altogether in some regions. At the very least, as is happening already, weather and wind patterns could shift multiyear ice far to the east in the Canada Basin, leaving large stretches of open water in the Chukchi and western Beaufort Seas.

Warmer temperatures and little sea ice cover in the Bering Sea are causing a shift in the size composition of crustacean zooplankton to smaller species, with potential food web implications. Warm years result in increases of zooplankton predators such as chaetognaths, another indication that global change may be having a significant impact on community organization [329]. Warmer temperatures also are thought to be accompanied by earlier and higher zooplankton production, with more of the ice-related and pelagic primary production going into the pelagic system, whereas cold temperatures result in later, lower zooplankton production with energy flowing predominately to the benthos [330]. Loss of energy to the benthos could result in major changes in the biomass and species assemblages of Bering Sea benthic communities and their avian and mammalian predators [253], [331].

The Chukchi Sea may be transformed into an ecological extension of the Bering Sea with a northward shift of the subarctic-to-arctic front that is accompanying warming [253]. This could lead to a decline in benthic infaunal biomass as the ecosystem shifts from benthic to pelagic dominance of organic matter consumption. The upper trophic structure could change significantly [262]. For example, a shift to pelagic production would favor some pelagic feeders, such as bowhead, fin, minke, and blue whales, but disadvantage benthic feeders, such as gray whales, walrus, bearded seals, and diving ducks [253], [272], [331].

The benthic food web could be affected by hydrographic changes that may accompany ice retreat. For example, samples taken during the Western Arctic Shelf-Basin Interactions project in 2002 in the Chukchi and Beaufort seas showed that large-bodied copepods are prevalent on the outer continental shelf. If ice retreats and there is strong upwelling, these copepods could end up on the inner shelf and possibly outcompete smaller zooplankters. Grazing by these species would probably drive the ecosystem toward a pelagic food chain. A mismatch of the timing of the phytoplankton bloom with the advection of large-bodied copepods into the shelf region would result in large exports to the benthic food chain [332].

Changes in community structure likely will accompany climate change [253], [329], [331], [333] as subarctic species move north and compete with the colder-water species present now. Mueter and Litzow [334] have estimated that the southern edge of the summer cold pool in the Bering Sea has retreated northward by about 230 km from the early 1980s to 2006 (but 2008-2009 saw the reemergence of an extensive cold pool on the Bering Sea shelf). Subarctic taxa, which were at the northern limit of their thermal tolerance, are now in areas formerly covered by the cold pool: the centers of distribution of 40 taxa, including pollock, halibut, rock sole, and snow crab, have moved northward an average of 34 km [334].

Pacific species of mollusks and crabs have moved north into the Chukchi Sea [270]. NOAA [278] has documented fish of six species that have extended their range into the Beaufort Sea from the Chukchi or Bering seas. Displacement of fish stocks northward is likely to be accompanied by (1) economic stresses as fishing fleets are compelled to expand their operations at higher fuel costs, and (2) geopolitical stresses if the U.S. fish stocks relocate to near the boundary with Russia, adding a layer of complexity to the management of these species. On the positive side, northward movement of fish stocks could provide additional subsistence fishing opportunities for local communities.

Foraging and resting habitats also will change. Sea ice substratum provides the resting and foraging base for ice seals, walrus, and polar bears [335], [336]. If it is not available, or the ice moves offshore into deep waters, the normal benthic or pelagic prey base may be unavailable. Moving between distant floes also will cost energy. There may be a decline in reproductive success for those mammals, such as ice seals, that den and raise their pups on the ice [259]. Loss of sea ice also could cause overcrowding of land haulouts, as occurred with walrus on the Chukotka Peninsula in 2007, when thousands of individuals were crowded into a small beach and many died from suffocation and injuries [337]. The loss of sea ice as a hunting platform for local native residents could have a profound impact on their subsistence hunting success, and it would be more dangerous to hunt in more open water [259].

Increased temperatures have already led to increased river runoff in the Arctic, which could change nutrient content, increase sediment loads, and decrease salinities in nearshore waters. This could reduce benthic biomass and diversity [262]. Nearshore benthic communities also could undergo significant change induced by reduced ice cover, longer open-water season, changing flows through the Bering Strait, increased frequency and intensity of storms, increased river and freshwater runoff, and increased ice scouring and coastal erosion.

A shipping assessment projected through 2020 by the Arctic Council [263] concluded that Arctic natural resource development (hydrocarbons, hard minerals, and fisheries) and regional trade will be key drivers of marine activity. Oil and gas development in particular may increase significantly. The MMS currently has about 5,400 km^2^ under lease in the Beaufort Sea and about 11,000 km^2^ in the Chukchi Sea. The most recent lease sale, Chukchi Sea Lease Sale 193, held on February 6, 2008, broke records with 667 bids on 488 blocks. Four additional lease sales are planned for 2009-12 [338], although they may be delayed or stopped by litigation.

Such activities will result in increased shipping. The most significant threat from ships to the Arctic marine environment is the release of oil through accidental or illegal discharge. Oil can reduce insulating properties of marine mammals and seabirds, causing hypothermia, and can be fatal if ingested, inhaled, or absorbed. Birds and mammals are typically concentrated in leads and polynyas, increasing the risk to these animals if there is an oil spill. Other potential impacts of shipping are ship strikes on mammals, introduction of alien species through fouling and in ballast water, disruption of migratory patterns of mammals, noise, and garbage and other debris. Release of debris can cause entanglement, introduction of bacteria and disease, and ingestion of plastics and other foreign items [263].

Ocean acidification could have an impact on the prey base, fisheries species, and deepwater corals. In particular, calcification by bivalves that dominate polar shelves could be adversely affected, and thus the food web that relies on them [339]. Cooley and Doney [340] estimated that mollusk stocks contributed $748 million (19%) of the $3.8 billion ex-vessel revenues of the annual domestic U.S. commercial fisheries harvest in 2007. To the extent mollusk populations are diminished or imperiled, there could be revenue declines, job losses, and indirect economic costs. Ocean acidification also is predicted to reduce the absorption of low-frequency sound, leading to a noisier environment for marine mammals [341].

The marine ecosystems and their biodiversity will be changing dramatically over the coming decades. Surveys and continued monitoring of organisms will be needed, particularly in highly diverse areas. Long-term monitoring programs are expensive and a coordinated plan will be needed. Such planning for Arctic marine biodiversity is under way by the Marine Expert Monitoring Group, which is implementing the Circumpolar Biodiversity Monitoring Program under the umbrella of the Conservation of Arctic Fauna and Flora working group of the Arctic Council [342]. This multinational effort is being designed to identify biodiversity changes within a reasonable timeframe, to identify possible links between biodiversity trends and anthropogenic stressors, and to make information available and useful to managers in developing strategies for sustainable use of Arctic living resources. Biodiversity indices must cover central physical and biological elements in an ecosystem, include organisms important to local human communities, be relatively simple to measure, and be sensitive to ecosystem change. Monitoring sites must be in focal marine areas that already have long datasets, are biological hot spots (e.g., marginal ice zones, polynyas, boundaries, and fronts), and are key to biogeochemical properties, biota, and invasive species. For example, the Pacific-Arctic Gateway through the Bering Strait would be an ideal monitoring area because of increased heat and freshwater flow, increased marine mammal migration, declining sea ice cover, increased oil and gas exploration, exploratory fisheries in the last decade, and flow of pollutants, especially persistent organic pollutants largely from Asia.

In establishing such programs, it also will be critical to determine which taxon level is most cost-effective to monitor. For example, detecting invasive species will require monitoring at the species level, but for some macroalgal assemblages, Konar and Iken [343] have shown that monitoring higher taxonomic levels or functional groups might be more appropriate to help eliminate environmental “noise” caused by natural variation.

#### Census of Marine Life Contributions in the Alaska region

Three Census projects have been active in Alaska. NaGISA has sampled 11 sites in the Gulf of Alaska and 2 in the Beaufort Sea. The GoA sites are rocky macroalgal and seagrass habitats at Kodiak Island, Kachemak Bay, and Prince William Sound. A notable discovery was a bed of rhodoliths (free-living calcareous red algae) in Prince William Sound, a significant northward extension of their distribution [344]. Another finding in the GoA is that intertidal macroalgal biodiversity is among the highest in the world. In sampling along the Beaufort Sea coast, four species of brown and red macroalgae found in the Boulder Patch may represent invasions since studies in the 1980s. The boulder field in Camden Bay, discovered by NaGISA, has less encrusting coralline red algae and harbors particularly high abundances of the gastropod *Boreocingula martyni*, which was only rarely found in the Boulder Patch [320]. The 71 taxa found exceeded numbers reported for soft-bottom environments in the Beaufort Sea, but was lower than in the Boulder Patch in Stefansson Sound. Because species richness is comparable in the two regions, both should be considered biodiversity hot spots. These findings are opening interesting new questions about the dispersal potential and exchange of larvae between these boulder communities. They will provide critical benchmarks against which to evaluate ecosystem change in the Arctic and for restoration should an oil spill or other catastrophe occur.

ArcOD examines the full spectrum of marine life from microbes to mammals in all oceanographic realms, from the shallow shelves to the deep basins. About 30 expeditions or field activities were related to ArcOD, including many conducted within the framework of the International Polar Year Arctic Marine Biodiversity cluster, for which ArcOD was the lead project. Scientific exploration and discovery included (1) expeditions into previously understudied areas, such as the deep Canada Basin, (2) investigation of previously understudied taxonomic groups, such as gelatinous zooplankton, (3) study of understudied habitats, such as sea ice pressure ridge systems and seafloor pock marks, and (4) establishing time-series to begin assessing change, for example for zooplankton in the Canada Basin and for various taxonomic groups in the Chukchi Sea. New expeditions and investigations of previously collected material have yielded over 40 species new to science. Algae such as diatoms and flagellates, and meiofaunal invertebrates thrive in this environment in concentrations of thousands of individuals per liter [257], [345]. In the sea ice, researchers discovered a new genus and species of hydroid that moves about 20 cm per hour [346], possibly a key predator because it devours tiny shrimplike crustaceans, and was named *Sympagohydra tuuli* after the discoverers' newborn daughter [347], [348]. The first biological data ever collected on sea ice ridges in the Alaska region suggest high potential importance of these structures at a time of ice shrinkage because of the abundance of ice meiofauna and under-ice amphipods is high along their deep-reaching keels [334].

In the pelagic realm, ArcOD has sampled throughout the Arctic (e.g., [349]) and has undertaken the largest consolidation of zooplankton records to date. Using high-definition cameras on an ROV, researchers found many new species and more than 50 taxonomic categories of gelatinous zooplankton: almost two-thirds were medusae, one-fifth was siphonophores, and one-tenth was larvaceans [349]. In the benthic realm, ArcOD has undertaken one of the most extensive deep-sea sampling efforts of the Canada Basin in- and epi-fauna [305], [350]. The first new species formally described from the 2005 expedition was a seafloor polychaete *Sigambra healyae* named in honor of the research vessel, the U.S. Coast Guard Cutter *Healy* [351]. ArcOD affiliates have also undertaken the largest examination of fish species known for the Chukchi Sea [352] and established species lists and patterns of community structure for Chukchi Sea pelagic, benthic, and fish assemblages [270].

CMarZ is linked to an ongoing, multidisciplinary monitoring program along the Seward Line in the northern Gulf of Alaska that has been operating for over a decade, with an emphasis on seasonal and interannual patterns of the zooplankton species inhabiting the upper 100 m of water [276], [277]. Diversity is lower than in warmer waters, but biomass and productivity are high. As for most planktonic systems, the dominant species change as the seasons progress, and in warmer years there are variable contributions from expatriated southern species. An as-yet-unanalyzed series of collections down to 600 m holds the promise of understanding the mesopelagic realm in this region.

## Discussion

Knowledge about marine biodiversity of the U.S. is extensive owing to two or three centuries of its study in many places and by a variety of enterprises. These enterprises include governmental agencies from federal to local levels, with mandates both to extract and to protect marine resources, living (e.g., fisheries) and not (e.g., petroleum and other minerals). The innumerable academic institutions with shore facilities for study of the marine environment (private ones such as the Duke University Marine Laboratory and public ones such as Friday Harbor Laboratories of the University of Washington) have provided foci of research and knowledge. Other academic resources are seagoing facilities and natural history museums, supported both publicly (such as the U.S. National Museum of Natural History and the Natural History Museum of Los Angeles County) and privately (such as the B.P. Bishop Museum in Honolulu and the American Museum of Natural History in New York City). Non-American contributions to knowledge of U.S. marine biodiversity have been from expeditions such as the British *Challenger* (in Hawaii) and Japanese ones in Alaska.

However, as is clear from the biotic diversity inventories compiled in these six sections as an activity of the Census, knowledge of U.S. marine biodiversity is fragmentary. Lists, such as these for regions and the composite one for the country as a whole (Tables 1 and S1), are essential as baselines for making management decisions and for assessing biotic changes – both good and bad [122]. However, a national list is no better than the best of the regional ones, and ever the most complete list – for the Gulf of Mexico – is based on records scattered in space and time. Comparable knowledge is available for only parts of the other areas, such as the MHI and the Bay of Fundy, and less taxonomically extensive inventories are more common. That of Cobscook Bay, Maine, is notable for its temporal depth, a dimension missing from most compilations in any explicit way.

Knowledge is also uneven taxonomically. Even animals exploited commercially are incompletely known: Eschmeyer [353] estimated that 200 new species of fishes are being described worldwide each year, and thus it is very likely that new species remain to be discovered in all parts of the U.S. As a generalization, body size is directly correlated with knowledge of a species. And knowledge is uneven spatially, diminishing with depth and with distance from shore. Biodiversity is commonly tallied by species, but ecosystem diversity and genetic diversity are also essential to understand (e.g., [2], [9]). A major challenge is to interrelate components assessed at different levels of space, time, and taxonomic resolution. As the volume of genetic data grows (e.g., the ICoMM inventory), cross-referencing the sorts of “instantaneous” assessments of biodiversity in these data with the metrics that are common in policy, legislation, and public information will become increasingly important. Further, microbial data are currently available for discrete times and places [354]; integrating them meaningfully with species inventories over large spans of space and time is one of the great challenges, because it is through these small organisms that the range shifts resulting from human agency or global change are likely first to be perceived in the future.

Health of a marine system is not necessarily directly proportional to its biodiversity. In general, a naturally species-poor system seems to function as well as a naturally richer one – health appears to be related to the degree that an ecosystem is intact [2]. An ecosystem with redundancy contains multiple species that perform similar functions, but in a system with low redundancy, the loss of a species may remove a particular function; it is feared that Hawaii represents such an unresilient system. This condition may be related to Hawaii's having such a large proportion of endemic species, which are typically characterized by narrow habitat and physiological tolerances.

Another point of consensus among the sections is the inventory of threats to marine biodiversity. Indeed, most threats identified for the U.S. are true for the entire world. Foremost is overexploitation of living resources, especially fishes and invertebrates for food, by both commercial and recreational fishers. However, in Hawaii aquarium fishes now constitute the most valuable fishery, and in some places there is a souvenir trade in mollusk shells and turtle carapaces. Coastal development removes land from its natural function, commonly perturbs the seafloor during the building process, and reduces water quality by introducing sediment, chemicals, and particularly nutrients into the marine environment during the building process and afterward. Shipping presents dangers both on- and offshore from pollution by purposeful and accidental release of oil and other noxious substances, by transporting potentially invasive species, and, on coasts, by groundings. The consequences of global change include rising sea levels attendant upon rising temperatures, shifting currents, and rising concentrations of carbon dioxide in the surface ocean, all of which are likely to affect the geographic and bathymetric distributions of marine organisms. Such changes, along with diminished water quality, probably contribute to the growing number and size of hypoxic or anoxic areas, such as those in the Gulf of Mexico and along the West Coast, which have profound biotic impacts. Warming poses a different threat in Alaska – it reduces the amount, duration, and thickness of ice, which is an essential component of the habitat for organisms from polar bears to some hydroids. Perhaps a combination of global change and other indirect human perturbations to the marine environment account for the apparent growth in number and duration of harmful algal blooms: in recent years New England has experienced some of the worst episodes on record. Increased carbon dioxide has already lowered the pH of the surface ocean; this is expected to have a negative effect on survival of plankton, the base of the marine food chain, and the growth and health of corals, which form biodiverse reefs in shallow waters of the Hawaiian Islands and Florida, and deep reefs in Alaska and the Southeast U.S. Invasive species are increasingly being recognized. In some cases their introduction was due to clear human agency, such as the Indo-Pacific lionfish (*Pterois*) that was brought to the coast of the Southeast U.S. by home aquarists. The orange cup coral (*Tubastraea coccinea*), also from the Indo-Pacific, lives on oil platforms in the Gulf of Mexico, a habitat of human construction. Warming seas are likely to enhance both extirpations and introductions, making some areas inhospitable for organisms that have long lived there while allowing others to establish populations. A complete biotic inventory for an area is essential to recognize invasive species before they become conspicuous by the damage they inflict, and to recognize that species previously present are gone before their absence has follow-on effects.

Certainly more information must be obtained through field and laboratory research and monitoring. Innovations in sampling ranging from genetics to acoustics have been identified in these sections. But the sections also make clear that many more data currently exist than are easily accessible. Mobilizing those resources so they are available in an integrated data system would enhance knowledge of marine biodiversity in the U.S. enormously, and would be an important step in monitoring the effects of management practices and identifying threats to the biota.

Biodiversity databases, which can be useful as management tools as well as for basic science, are commonly organized and searchable taxonomically and geographically, but temporal organization is also essential so that trends through time can be identified (e.g., [16]). An apparent limitation is the existence of historical records. But, as has been shown repeatedly by the Census History of Marine Animal Populations (HMAP) project, they do exist, some in unlikely forms, and can be mobilized. A species list for a place, a habitat, or an ecosystem is a beginning, necessary but not sufficient for scientific understanding and development of long-term policy. Species richness (number of species) is the simplest measure of diversity, but knowledge of evenness, relative abundance, and dispersion [355] is also needed. An example of the sorts of biotic data available but not accessible are those from fisheries surveys concerned with nontarget species. Integrating such information can provide a much fuller inventory of the ecosystem to which the target species belongs than is now possible. But integrating data over space and time can be difficult – techniques must be developed to make certain that scales are compatible, to combine and reconcile data collected for various purposes with disparate gear, and to automate taxonomic changes. Interactive links between information on biotic features and that on abiotic elements of the environment will provide insights. OBIS, an initial Census effort at assembling the more obvious datasets, including some of those from Census projects, provides tools to create maps so organism distributions can be visualized, and to model potential distributions based on abiotic characteristics of the environment.

Three other impediments to assembling existing data and collecting new data on marine biodiversity were explicit or implicit in all sections: logistical problems, shortages in finances, and shortages in taxonomic expertise. Finances, of course, are essential to provide the other two. Offshore and deep-water biotas are more poorly known than those of the coasts largely because of the expense and logistical challenges of operating there, as is also true for high latitudes. A threat not to biodiversity itself but to increasing the knowledge of it is a continuing downturn in training of taxonomists, a problem that is particularly acute for taxa not of direct human relevance. Molecular techniques are improving for routine identifications, but taxonomists will continue to be essential to identify specimens from which molecules are then extracted, to identify poorly-known taxa, and to describe new taxa. And all sections demonstrated that progress in understanding marine biodiversity was enhanced by activities of the Census that built capacity and international collaborations because limited resources of equipment, knowledge, and money could be shared.

## Supporting Information

Table S1 Biotic diversity of the six U.S. geographically-based sections in the text, and a worldwide estimate.
(0.15 MB DOC)

Table S2 Taxonomic detail of species of the Northeast U.S. Continental Shelf Large Marine Ecosystem in registers (first three columns) and provisional additions identified from a survey of three databases.
(0.11 MB DOC)

Table S3 Taxonomic detail of species of the Northeast U.S. Continental Shelf Large Marine Ecosystem from databases.
(0.08 MB DOC)

Table S4 Significant databases containing biodiversity information for inclusion in the Ocean Biogeographic Information System (OBIS - http://www.iobis.org/) (from described surveys in five of the six regions described in this overview).
(0.12 MB DOC)

Table S5 Assessment of marine biodiversity, represented as number of described species by phylum, in the Southeast U.S. Continental Shelf Large Marine Ecosystem.
(0.08 MB DOC)

Table S6 Compared assessments of large scale/regional marine biodiversity (Insular Pacific-Hawaiian Large Marine Ecosystem vs. worldwide estimate), represented as number of described species by phylum.
(0.08 MB DOC)

Table S7 Alaska regional estimates of marine species, represented as number of described species by phylum (Bruce Wing, NOAA Auke Bay Lab, Juneau).
(0.10 MB DOC)

Text S1 Abbreviations from An Overview of Marine Biodiversity in United States Waters.
(0.03 MB DOC)

Text S2 Taxonomic and Regional Guides for the Northeast U.S. Continental Shelf, Southeast U.S. Continental Shelf, Gulf of Mexico, California Current and Gulf of Alaska, Eastern Bering Sea, and Aleutian Islands, and Chukchi and Beaufort Seas Large Marine Ecosystems.
(0.17 MB DOC)

Text S3 List of Contributors to the Harte Research Institute for Gulf of Mexico Studies Gulf of Mexico Biodiversity Project [Felder DL, Camp DK (eds) (2009) Gulf of Mexico Origin, Waters, and Biota. Volume 1, Biodiversity. College Station, Texas: Texas A&M University Press. 1384 p.]
(0.12 MB DOC)

Text S4 California Current Large Marine Ecosystem Contributing Taxonomic Experts
(0.03 MB DOC)
