# Supplementary material for: An Overview of Marine Biodiversity in United States Waters
Source: PLoS One. 2010 Aug 2;5(8):e11914. doi: 10.1371/journal.pone.0011914 (PMC2914028; doi:10.1371/journal.pone.0011914)
Supplement: Table S1 — Biotic diversity of the six U.S. geographically-based sections in the text, and a worldwide estimate. (0.15 MB DOC) [file pone.0011914.s001.doc]

**Table S1. Biotic diversity of the six U.S. geographically-based sections in the text, and a worldwide estimate.** This table provides greater detail than Tables 3, 5, 6, 7, 9, and 10.

|  | **Northeast U.S. Continental Shelf LME** | **Southeast**  **U.S. Continental Shelf LME** | **Gulf of Mexico** | **Insular-Pacific Hawaii LME** | **California Current LME** | **Arctic Ocean** | **Worldwide Estimate** |
| --- | --- | --- | --- | --- | --- | --- | --- |
| **Taxon** |  |  |  |  |  |  |  |
| Bacteria | 1 | 32 | UD | UD | UD | UD | 4800 |
| Cyanophyta/ Cyanobacteria | 9 | 16 | 45 | 183 | UD | UD | 1000 |
| Ciliophora |  |  | 574 | UD | 28 | 324 |  |
| Radiolaria |  |  | # | UD | UD | 40 | 550 |
| Fungi |  |  | 54 | 55 | UD | UD | 500 |
| Chlorophyta | 98 | 65 | 195 | 247 | 139 | 15^1^ | 2500 |
| Foraminifera | 2 | 165 | 951 | 755 | 670 | 325 | 10,000 |
| Bacillariophyta | 222 |  | 948 | 91 | UD | 287 | 5000 |
| Phaeophyta | 154 | 217 | 86 | 84 | 187 | 25^1^ | 1600 |
| Rhodophyta | 148 | 38 | 392 | 574 | 557 | 18^1^ | 6200 |
| Plantae |  |  | 10 | UD | 7 | 1 |  |
| Angiospermae |  | 10 | 370 |  |  |  |  |
| Dinoflagellates | 49 |  | 644 | 43 | UD | 70 |  |
| Porifera | 36 | 111 | 339 | 144 | 134 | 163 | 5500 |
| Placozoa |  | 1 | UD | 1 | 1 |  |  |
| Cnidaria | 212 | 362 | 792 | 460 | 400 | 227 | 9795 |
| Ctenophora | 5 |  | 18 | 14 | 28 | 6 | 166 |
| Platyhelminthes | 77 |  | 705 | 676 | 1389 | 134 | 15,000 |
| Dicyemida/Rhombozoa |  |  | 7 | UD | 18 |  | 82 |
| Orthonectida |  |  | UD | UD | 4 |  | 24 |
| Nemertea | 37 |  | 42 | 49 | 200 | 79 | 1180–1230 |
| Rotifera | 4 |  | 23 | 3 | 10 | 16 | 50 |
| Gastrotricha |  |  | 42 | 2 | 38 | 28 | 390–400 |
| Kinorhyncha |  |  | 2 | 2 | 8 | 11 | 130 |
| Nematoda | 28 |  | 190 | 54 | 200 | 362 | 12,000 |
| Nematomorpha | 2 |  | UD | 4 | 0 | 0 | 5 |
| Acanthocephala | 31 |  | 33 | 6 | 70 | 0 | 600 |
| Entoprocta |  |  | 2 | 2 | 10 | 0 | 165–170 |
| Gnathostomulida |  |  | 19 | 8 | 1 | 1 | 97 |
| Priapulida |  |  | 1 | 1 | 1 | 5 | 8 |
| Loricifera |  |  | UD | UD | 1 | 0 | 18 |
| Cycliophora |  |  | # | UD | 0 | 0 | 1 |
| Sipuncula | 18 |  | 27 | 14 | 8 | 12 | 144 |
| Echiura | 7 |  | 7 | 6 | 2 | 2 | 176 |
| Annelida | 689 | 400 | 866 | 343 | 830 | 533 | 12,000 |
| Pogonophora |  |  | UD | 2 | 15 | 9 | 148 |
| Tardigrada |  |  | 14 | 2 | 13 | 7 | 212 |
| Crustacea | 810 | 696 | 2579 | 1325 | 2,680 | 1525 | 44,950 |
| Chelicerata  (non-arachinid) | 26 |  | 59 | 19 | 30 | 38 | 2,267 |
| Mollusca | 868 | 698 | 2455 | 1345 | 663 | 488 | 52,525 |
| Phoronida | 1 | 2 | 3 | 5 | 7 | 1 | 10 |
| Bryozoa/Ectoprocta | 138 | 91 | 266 | 168 | 150 | 331 | 5700 |
| Brachiopoda | 1 |  | 26 | 10 | 5 | 9 | 550 |
| Echinodermata | 138 |  | 522 | 309 | 290 | 151 | 7000 |
| Chaetognatha | 6 | 33 | 24 | 18 | 39 | 13 | 121 |
| Hemichordata | 5 |  | 5 | 3 | 23 | 1 | 106 |
| Urochordata | 44 | 35 | 102 | 102 | 62 | 64 | 4900 |
| Cephalochordata | 2 | 5 | 5 | 3 | 1 |  | 32 |
| Vertebrata | [1174] | [1300] | [1975] | [1295] | [1043] | [513] | [16,585] |
| Pisces | 954 | 1200 | 1541 | 1214 | 909 | 415 | 16,475 |
| Reptilia | 4 | 7 | 9 | 5 | 4 | 0 |  |
| Aves | 182 | 73 | 395 | 52 | 92 | 82 |  |
| Mammalia | 34 | 20 | 30 | 24 | 38 | 16* | 110 |
|  |  |  |  |  |  |  |  |
| **Total (est.)** | **5,042^1^** | **4,277^2^** | **15,419^3^** | **8,427^4^** | **10,160^5^** | **5,834^6^** | **224,787^7^** |

**NOTES**:

UD number undetermined

# data not provided

* species reported in the Arctic Ocean range from 14 to 16

[ ] sum of vertebrate constituent groups

^1^Information also available in Table 3.

^2^Information also available in Table 5.

^3^Information also available in Table 6. Includes 54 species of fungi.

^4^Information also available in Table 7. Includes 55 species of fungi.

^5^Information also available in Table 9. Includes 198 parasite-only other protozoans (Haplosporida, Microsporida, Myxosporida, Sarcomastigophora, Sporozoa).

^6^Information also available in Table 10. Values for Chlorophyta, Phaeophyta and Rhodophyta are from the Alaska LMEs; other values are from the broader Arctic (Bluhm, Gradinger, and Hopcroft, as reported in Table 11).

Values are from the following resources:

**Northeast U.S. Continental Shelf LME**: Values represent an assessment of marine biodiversity in the Virginian and Gulf of Maine/Bay of Fundy Ecoregions based on species registers and a search of available databases. Many more entries could be made from other archived sources, and some sources are being prepared for release.

**Southeast U.S. Continental Shelf LME**: Values were compiled and estimated based on information from sub-regions (South Atlantic Blight and Florida East Coast; Cape Hatteras to Cape Canaveral; and Florida Keys). A full list of the experts who provided information for these estimates can be found in the Southeast U.S. Continental Shelf LME Acknowledgements.

**Gulf of Mexico LME**: Values are from Felder DL, Camp DK (eds) (2009) Gulf of Mexico Origin, Waters, and Biota. Volume 1, Biodiversity. College Station, Texas: Texas A&M University Press. 1384 p.

**Insular Pacific –Hawaii LME**: Values are based on the work of Lucius E. Eldredge and Isabella Abbott and personal communications.

**California Current LME**: Values are based on publications and experts cited in Texts S2 and S4. Most sources are listed by phylum.

**Alaskan LMEs**: There is no comprehensive list of species for all of Alaska, which includes four Large Marine Ecosystems (East Bering Sea; Gulf of Alaska; Chukchi Sea; Beaufort Sea). Bodil Bluhm, Rolf Gradinger and Russ Hopcroft, all associated with the Census project ArcOD, used data sources and their experience to compile the most recent species list for the Arctic Ocean (Table 11 in Alaskan LME section), which is based heavily on Sirenko (2001). The number of seaweed species is based on the Alaska Seaweed Database (<http://herbarium.botany.ubc.ca/herbarium_data/algae_alaska/search.htm>). Bruce Wing (Curator, Reference Collections, Auke Bay Laboratories, AFSC/NMFS/NOAA/DOC, Ted Stevens Marine Research Institute, 17109 Pt. Lena Loop Road, Juneau, AK 99801, [Bruce.Wing@noaa.gov](mailto:Bruce.Wing@noaa.gov)) has estimated 542 total species in the Gulf of Alaska, 572 in the Bering Sea and Aleutian Islands, and 220 species in the Arctic (Table S6). The disparity between the Arctic estimates in the two tables illustrates the difficulties of attaining agreement on the exact numbers of Alaskan species. Plankton species estimates for the Gulf of Alaska and Bering Sea can be found in Table 9, and examples of invertebrate taxa in various regions can be found in Table 10.

**^7^Worldwide Estimate**: Values are based upon Bouchet P (2006) The magnitude of marine biodiversity. In: Duarte CM, editor. The Exploration of Marine Biodiversity Scientific and Technological Challenges. Bilbao: Fundación BBVA. pp. 31–62.
