# Supplementary material for: An Overview of Marine Biodiversity in United States Waters
Source: PLoS One. 2010 Aug 2;5(8):e11914. doi: 10.1371/journal.pone.0011914 (PMC2914028; doi:10.1371/journal.pone.0011914)
Supplement: Table S2 — Taxonomic detail of species of the Northeast U.S. Continental Shelf Large Marine Ecosystem in registers (first three columns) and provisional additions identified from a survey of three databases. (0.11 MB DOC) [file pone.0011914.s002.doc]

**Table S2. Taxonomic detail of species of the Northeast U.S. Continental Shelf Large Marine Ecosystem in registers (first three columns) and provisional additions identified from a survey of three databases.**

| **Taxon group** | **GoMRMS** | **Virginian (derived from NWARMS, see text for details)** | **Total in registers** | **Provisional additions from databases** | **Total species** |
| --- | --- | --- | --- | --- | --- |
| Bacteria | 1 |  | 1 |  | 1 |
| Cyanophyta/Cyanobacteria | 9 |  | 9 |  | 9 |
| Ciliophora |  |  |  |  |  |
| Radiolaria |  |  |  |  |  |
| Fungi |  |  |  |  |  |
| Chlorophyta | 98 |  | 98 |  | 98 |
| Foraminifera |  |  |  | 2 | 2 |
| Bacillariophyta | 222 |  | 222 |  | 222 |
| Phaeophyta | 154 |  | 154 |  | 154 |
| Rhodophyta | 148 |  | 148 |  | 148 |
| Plantae |  |  |  |  |  |
| Dinoflagellates | 49 |  | 49 |  | 49 |
| Porifera | 28 | 4 | 32 | 4 | 36 |
| Placozoa |  |  |  |  |  |
| Cnidaria | 170 | 22 | 192 | 20 | 212 |
| Ctenophora | 4 | 1 | 5 |  | 5 |
| Platyhelminthes | 72 | 4 | 76 | 1 | 77 |
| Dicyemida/Rhombozoa |  |  |  |  |  |
| Orthonectida |  |  |  |  |  |
| Nemertea | 35 | 1 | 36 | 1 | 37 |
| Rotifera | 4 |  | 4 |  | 4 |
| Gastrotricha |  |  |  |  |  |
| Kinorhyncha |  |  |  |  |  |
| Nematoda | 28 |  | 28 |  | 28 |
| Nematomorpha | 2 |  | 2 |  | 2 |
| Acanthocephala | 27 | 4 | 31 |  | 31 |
| Entoprocta |  |  |  |  |  |
| Gnathostomulida |  |  |  |  |  |
| Priapulida |  |  |  |  |  |
| Loricifera |  |  |  |  |  |
| Cycliophora |  |  |  |  |  |
| Sipuncula | 6 |  | 6 | 12 | 18 |
| Echiura | 1 |  | 1 | 6 | 7 |
| Annelida | 367 | 78 | 445 | 244 | 689 |
| Pogonophora |  |  |  |  |  |
| Tardigrada |  |  |  |  |  |
| Crustacea | 428 | 121 | 549 | 261 | 810 |
| Chelicerata (nonarachnid) | 12 | 2 | 14 | 12 | 26 |
| Mollusca | 378 | 309 | 687 | 181 | 868 |
| Phoronida | 1 |  | 1 |  | 1 |
| Bryozoa/Ectoprocta | 65 | 11 | 76 | 62 | 138 |
| Brachiopoda | 1 |  | 1 |  | 1 |
| Echinodermata | 62 | 11 | 73 | 65 | 138 |
| Chaetognatha | 5 |  | 5 | 1 | 6 |
| Hemichordata | 5 |  | 5 |  | 5 |
| Urochordata | 41 | 1 | 42 | 2 | 44 |
| Cephalochordata |  | 1 | 1 | 1 | 2 |
| Pisces | 504 | 373 | 877 | 77 | 954 |
| Reptilia | 2 | 2 | 4 |  | 4 |
| Aves | 182 |  | 182 |  | 182 |
| Mammalia | 27 | 7 | 34 |  | 34 |
| Unknown Protoctista | 3 |  | 3 |  | 3 |
| **Totals** | **3,141** | **952** | **4,093** | **952** | **5,045** |

**Note:** Data are number of species per taxon.
