# Supplementary material for: An Overview of Marine Biodiversity in United States Waters
Source: PLoS One. 2010 Aug 2;5(8):e11914. doi: 10.1371/journal.pone.0011914 (PMC2914028; doi:10.1371/journal.pone.0011914)
Supplement: Table S3 — Taxonomic detail of species of the Northeast U.S. Continental Shelf Large Marine Ecosystem from databases. (0.08 MB DOC) [file pone.0011914.s003.doc]

**Table S3. Taxonomic detail of species of the Northeast U.S. Continental Shelf Large Marine Ecosystem from databases.**

| **Taxon group** | **Occurring in both GoM and Virginian ecoregions** | **Unique to GoM samples** | **Unique to Virginian samples** |
| --- | --- | --- | --- |
| Bacteria |  |  |  |
| Cyanophyta/Cyanobacteria |  |  |  |
| Ciliophora |  |  |  |
| Radiolaria |  |  |  |
| Fungi |  |  |  |
| Chlorophyta |  |  |  |
| Foraminifera | 1 | 1 |  |
| Bacillariophyta |  |  |  |
| Phaeophyta |  |  |  |
| Rhodophyta |  |  |  |
| Plantae |  |  |  |
| Dinoflagellates |  |  |  |
| Porifera |  | 3 | 4 |
| Placozoa |  |  |  |
| Cnidaria | 15 | 16 | 16 |
| Ctenophora |  |  |  |
| Platyhelminthes |  |  | 1 |
| Dicyemida/Rhombozoa |  |  |  |
| Orthonectida |  |  |  |
| Nemertea |  |  | 2 |
| Rotifera |  |  |  |
| Gastrotricha |  |  |  |
| Kinorhyncha |  |  |  |
| Nematoda |  |  |  |
| Nematomorpha |  |  |  |
| Acanthocephala |  |  |  |
| Entoprocta |  |  |  |
| Gnathostomulida |  |  |  |
| Priapulida |  |  |  |
| Loricifera |  |  |  |
| Cycliophora |  |  |  |
| Sipuncula | 8 | 1 | 7 |
| Echiura | 1 | 1 | 4 |
| Annelida | 282 | 39 | 207 |
| Pogonophora |  |  |  |
| Tardigrada |  |  |  |
| Crustacea | 272 | 97 | 199 |
| Chelicerata (nonarachnid) | 11 | 9 | 5 |
| Mollusca | 225 | 65 | 189 |
| Phoronida | 1 |  |  |
| Bryozoa/Ectoprocta | 32 | 52 | 17 |
| Brachiopoda |  | 1 |  |
| Echinodermata | 55 | 29 | 30 |
| Chaetognatha |  | 1 |  |
| Hemichordata |  |  | 2 |
| Urochordata | 3 | 7 | 8 |
| Cephalochordata |  |  | 2 |
| Pisces | 167 | 7 | 260 |
| Reptilia |  |  | 1 |
| Aves |  |  |  |
| Mammalia |  |  |  |
| Unknown Protoctista |  |  |  |
| **Totals** | **1,073** | **329** | **954** |

**Note:** Number of species are shown that were common to both ecoregions, or occurred in just the GoM or Virginian ecoregions.
