# Supplementary material for: An Overview of Marine Biodiversity in United States Waters
Source: PLoS One. 2010 Aug 2;5(8):e11914. doi: 10.1371/journal.pone.0011914 (PMC2914028; doi:10.1371/journal.pone.0011914)
Supplement: Table S4 — Significant databases containing biodiversity information for inclusion in the Ocean Biogeographic Information System (OBIS - http://www.iobis.org/) (from described surveys in five of the six regions described in this overview). (0.12 MB DOC) [file pone.0011914.s004.doc]

**Table S4. Significant databases containing biodiversity information for inclusion in the Ocean** **Biogeographic** **Information** **System (OBIS -** <http://www.iobis.org/>**) (from described surveys in five of the six regions described in this overview).**

**Northeast U.S. Continental Shelf Large Marine Ecosystem***

| **Type of data** | **Data / Brief description** | **Illustrative references** |
| --- | --- | --- |
| Seafloor | usSEABED, U.S. Geological Survey (USGS) | <http://walrus.wr.usgs.gov/usseabed/> |
|  | Canadian Marine Multibeam Bathymetric Data, Natural Resources Canada | <http://gdr.nrcan.gc.ca/multibath/index_e.php> |
|  | Gulf of Maine Mapping Initiative | http://gulfofmaine.org/gommi/coverage-map.php |
| Benthic | Environmental Monitoring and Assessment Program, U.S. Environmental Protection Agency (EPA) | http://www.epa.gov/emap/nca/index.html |
|  | Northeast Fisheries Science Center Benthic database (NEFSC), National Marine Fisheries Service | Planned for OBIS |
|  | NOAA National Benthic Inventory | [http://nbi.noaa.gov](http://nbi.noaa.gov/) |
| Demersal | DFO Maritimes Research Vessel Trawl Surveys, Department of Fisheries and Oceans (DFO) Canada | Available through International OBIS  ([iobis.org](http://www.iobis.org/)) |
|  | NEFSC Bottom Trawl Survey, NMFS | Available through International OBIS  ([iobis.org](http://www.iobis.org/)) |
| Plankton | Bay of Fundy Phytoplankton Monitoring Program | Presently not available on-line |
|  | Continuous Plankton Recorder Survey | Presently not available on-line |
|  | Ecosystem Monitoring Program (EcoMon) | Presently not available on-line |
| Marine mammals and turtles | Marine mammals and turtle sightings and strandings | Available through OBIS-SEAMAP: <http://seamap.env.duke.edu/> |
| Seabirds | Historical seabird data for the U.S. Atlantic waters from Maine to Florida are currently in many separate databases. USGS, U.S. Fish and Wildlife, and Minerals Management Service are collaborating to unify and house a single database (in development) | Available through OBIS-SEAMAP: <http://seamap.env.duke.edu/> |
| Exotic species | National Estuarine and Marine Exotic Species Information System, Smithsonian Environmental Research Center | <http://invasions.si.edu/nemesis/index.html> |
|  | Non-indigenous Aquatic Species, U.S. Geological Survey and Smithsonian Environmental Research Center | [http://nas.er.usgs.gov](http://nas.er.usgs.gov/) |
| Long-term national monitoring programs | Long-Term Ecological Research Sites Data Catalog | <http://metacat.lternet.edu/knb> |
|  | National Estuarine Research Reserve’s System-wide Monitoring Program | <http://cdmo.baruch.sc.edu/> |
| Regional programs | Chesapeake Bay Program, EPA | <http://www.chesapeakebay.net/dataandtools.aspx?menuitem=14872> |
|  | Chesapeake Bay Ecosystem Integrated Information Systems (CBEIIS) will compile and collate data from more than 40 recent surveys and monitoring programs (in development by NOAA/NMFS/Chesapeake Bay Office and partners) |  |
|  | Chesapeake Bay Introduced Species Database, Smithsonian Environmental Research Center | <http://invasions.si.edu/nemesis/chesapeake.html> |
|  | Narragansett Bay Fish Trawl Survey, University of Rhode Island Graduate School of Oceanography | <http://www.gso.uri.edu/fishtrawl/home.htm> |
|  | Narragansett Bay.org Research Portal | <http://www.narrbay.org/> |
|  | New York Ocean and Great Lake Atlas, NY Ocean and Great Lakes Ecosystem Conservation Council | [http://nyoglatlas.org](http://nyoglatlas.org/) |
|  | Northwest Atlantic Eco-regional Assessment, The Nature Conservancy (in development) | <http://www.nature.org/initiatives/marine/strategies/assessments.html> |

***** This table includes key sources of data for the Virginian and Gulf of Maine/Bay of Fundy Ecoregions. The list is representative of available data, but is by no means exhaustive.

**Southeast U.S. Continental Shelf Large Marine Ecosystem**

| **Data set** | **Brief description** | **Illustrative references** |
| --- | --- | --- |
| MARMAP | Marine Resources Monitoring, Assessment and Prediction, a cooperative program between NOAA Fisheries Service and South Carolina Department of Natural Resources. A regional (SAB) fishery-independent sampling survey of demersal fishes (1973-present; 9-373 m depth). Several thousand fish collections in ACCESS database. | Wenner and Read (1982); Wenner (1983);  Wenner and Sedberry (1989);  Rowe and Sedberry (2006); Sedberry et al. (2006). |
| SEAMAP-SA | Southeast Area Monitoring and Assessment Program-South Atlantic. Coastal trawl survey database, SAB (1988-present). Several thousand fish collections, 4-19 m depth. | <http://www.dnr.sc.gov/marine/mrri/SEAMAP/SMreports.html> |
| NOAA-OE | National Oceanic and Atmospheric Administration, Office of Ocean Exploration: collections, videotape annotations, species lists. Submersible surveys in the SAB, 45-1000 m depth. | Schobernd and Sedberry (2009);  <http://www.ncddc.noaa.gov/interactivemaps/oceanexploration-digital-atlas-south-atlantic-bight> |
| NODC | National Oceanographic Data Center; houses data from biodiversity surveys and faunal characterizations of the continental shelf and slope, from surveys funded by the Minerals Management Service. | Sedberry and Van Dolah (1983);  Wenner et al. (1984) |
| SAFMC | South Atlantic Fishery Management Council | Internet map server that hosts MARMAP and other data: <http://ocean.floridamarine.org/efh_coral/ims/viewer.htm> |
| ONMS | Office of National Marine Sanctuaries: Gray’s Reef National Marine Sanctuary and Florida Keys National Marine Sanctuary: Management Plans and Condition Reports summarize monitoring and biodiversity surveys. | http://sanctuaries.noaa.gov/management/mpr/welcome.html  <http://sanctuaries.noaa.gov/science/condition/welcome.html> |
| NERRS | National Estuarine Research Reserves. NERRS conducts surveys and monitoring in each of the five sites in the region; some of these include biotic surveys. | Upchurch, S. and E. Wenner (2008); Ross and Bichy (2002) |

**Gulf of Mexico Large Marine Ecosystem**

| **Data set** | **Brief description** | **Illustrative references** |
| --- | --- | --- |
| NOAA-ELMR Program | National Oceanic and Atmospheric Administration, Estuarine Living Marine Resources Program; Spatial and temporal distribution of 44 key fish and invertebrate species in 31 northern Gulf of Mexico estuaries | Nelson (1992)  Patillo et al. (1997) |
| SEAMAP-Gulf | Southeast Area Monitoring and Assessment Program – Gulf of Mexico. Initiated in 1981. Annual plankton, trawl and reef fish surveys. (Gulf States Marine Fisheries Commission) | [www.gsmfc.org/programs](http://www.gsmfc.org/programs) |
| TPWD-CFMP | Texas Parks and Wildlife – Coastal Fisheries Monitoring Program. Initiated in 1975. Bag seine, trawl, oyster dredge and gill net data for all Texas bays and estuaries. | [www.tpwd.state.tx.us/fishboat/fish/management](http://www.tpwd.state.tx.us/fishboat/fish/management) |
| NOAA-NCDDC | National Oceanic and Atmospheric Administration, National Coastal Data Development Center: collections, videotape annotations, species lists. Submersible surveys in Gulf of Mexico. | [www.ncddc.noaa.gov/interactivemaps/gulf-of-mexico](http://www.ncddc.noaa.gov/interactivemaps/gulf-of-mexico) |
| NOAA-NODC | National Oceanic and Atmospheric Administration, National Oceanographic Data Center; houses data from biodiversity surveys and faunal characterizations of the continental shelf and slope, from surveys funded by the Minerals Management Service | [www.nodc.noaa.gov/](http://www.nodc.noaa.gov/) |
| GMFMC | Gulf of Mexico Fisheries Management Council; fisheries management data and regulations | [www.gulfcouncil.org/](http://www.gulfcouncil.org/) |
| NOAA-NMSP | National Oceanic and Atmospheric Administration, National Marine Sanctuary Program: Flower Gardens National Marine Sanctuary and Florida Keys National Marine Sanctuary; monitoring, assessment, and biodiversity surveys and information | [www.sanctuaries.noaa.gov/](http://www.sanctuaries.noaa.gov/) |

**California Current Large Marine Ecosystem**

| **Data set** | **Description** | **Illustrative references** |
| --- | --- | --- |
| CALCOFI | The California Cooperative Oceanic Fisheries Investigations are a partnership of the California Department of Fish and Game, NOAA Fisheries and the Scripps Institution of Oceanography. Formed in 1949 to study ecological aspects of the CA sardine fishery collapse, it makes available long-term datasets, atlases and reports on zooplankton and other physical and environmental collected off the California coast. | [www.calcofi.org](http://www.calcofi.org/) |
| NWFSC | The Northwest Fisheries Science Center provides three primary data sets through its Web site: West Coast Habitat Server is an interactive application and mapping tool for California Current fisheries and habitat information; the Ocean Index Tool uses biological and physical indicators to forecast salmon; and the Scientific Data Management Team portal provides access to a variety of NWFSC data sets. | [www.nwfsc.noaa.gov](http://www.nwfsc.noaa.gov/) |
| PACOOS | Pacific Coast Ocean Observing System is a partnership of NOAA, academia, foundations, and state agencies that provides access to, and integrates marine resource information for, the California Current system. Available information includes several regional ocean observing systems, CALCOFI, Cooperative Zooplankton Dataspace and the West Coast Habitat Server. | [www.pacoos.org/Datamgt.htm](http://www.pacoos.org/Datamgt.htm) |
| PSMFC | Pacific States Marine Fisheries Commission data programs collect, consolidate, and distribute information on fishery resources in California, Oregon, Washington, Idaho and Alaska. These include the Pacific Fisheries Information Network (PacFIN) providing up-to-date information on commercial fish catches, the Recreational Fisheries Information Network (RecFIN) providing biological and socioeconomic data on recreational fisheries, and habitat programs. | [www.psmfc.org/Data_Programs](http://www.psmfc.org/Data_Programs) |
| PISCO | The Partnership for Interdisciplinary Studies of Coastal Oceans is a consortium of four universities that integrates research and monitoring over an area extending more than 1,200 miles along the Pacific coast. PISCO provides a data catalog access portal, a subtidal community survey map, and a marine taxonomic database. | [www.piscoweb.org/data/access-and-applications](http://www.piscoweb.org/data/access-and-applications) |
| ONMS | Office of National Marine Sanctuaries: West Coast sanctuaries (Olympic Coast, Cordell Bank, Gulf of the Farallones, Monterey Bay, Channel Islands) are actively involved in identifying and assessing natural and cultural resources, monitoring the status and trends of marine life and habitats, and supporting targeted research projects to address other information needs. | [sanctuaries.noaa.gov/management/mpr/welcome.html](http://sanctuaries.noaa.gov/management/mpr/welcome.html)  [sanctuaries.noaa.gov/science/condition/welcome.html](../sanctuaries.noaa.gov/science/condition/welcome.html) |
| SWFSC | Southwest Fisheries Science Center maintains a data portal to CALCOFI, PACOOS, and local oceanographic information and provides access to marine mammal and turtle genetics samples. | [swfsc.noaa.gov](../swfsc.noaa.gov) |
| TNC | The Nature Conservancy has conducted Pacific coast marine ecoregion assessments from Baja California to Vancouver Island and from the shore out to the edge of the continental slope. The assessments are designed to inform conservation, help organize and update biodiversity information, produce spatial databases and maps and provide benchmarks for monitoring environmental trends. Many are contiguous with those for terrestrial ecoregions. TNC also supports MarineMap, a GIS tool with biodiversity layers. | [conserveonline.org/workspaces/MECA](../conserveonline.org/workspaces/MECA)  [www.marinemap.org/mlpa](http://www.marinemap.org/mlpa) |

**Alaska’s Large Marine Ecosystems – the Gulf of Alaska, Eastern Bering Sea and Aleutian Islands, and Chukchi and Beaufort Seas**

| **Data set** | **Brief description** | **Illustrative references** |
| --- | --- | --- |
| PMEL | Pacific Marine Environmental Laboratory (PMEL) in Seattle. Includes NOAA data, mostly physical, but also biological information from across the North Pacific, including datasets from foreign scientific institutions and government agencies. | <http://www.pmel.noaa.gov/datalinks.html> |
| AFSC | Alaska Fisheries Science Center (AFSC) databases and tools on fishery, oceanography, marine mammal, and environmental research compiled and designed by AFSC scientists are used to develop policies and strategies for fisheries management within the U.S. Exclusive Economic Zone, to monitor and manage the health of the region's marine mammal populations, and to assess the impacts of chemical contaminants and physical alterations on select organisms and marine habitats. The data, some dating back nearly 100 years, are obtained from boat surveys, aerial surveys, land counts, stream and beach surveys, and other miscellaneous sources. Some databases are available for downloading from the AFSC Web site or from database managers. | <http://www.afsc.noaa.gov/databases.htm>  Of specific relevance to the Ocean Biogeographic Information System (OBIS) may be the following:  (1) Groundfish survey data from Gulf of Alaska and Bering Sea and Aleutians. See: <http://www.afsc.noaa.gov/RACE/groundfish/survey_data/default.htm>  (2) Icthyoplankton cruise data. See <http://access.afsc.noaa.gov/icc/index.cfm>  (3) Nearshore Fish Atlas of Alaska - Auke Bay Laboratories. See: <http://www.afsc.noaa.gov/ABL/datasets/abstracts/ab_Fish_atlas-Lindeberg-updated11-25-08.pdf> |
| NPRB | The North Pacific Research Board has funded extensive marine research since 2002. Data from those projects are available on NPRB’s Web site and in the Alaska Marine Information System, a joint data system under development by NPRB, the University of Alaska Fairbanks, and the Alaska Ocean Observing System. | [www.nprb.org](http://www.nprb.org/);  <http://ak.aoos.org/amis/> |
| PICES | The North Pacific Marine Science Organization (PICES) established the Technical Committee on Data Exchange (TCODE) to gather metadata records from all PICES countries around the North Pacific. | See PICES Metadata Federation at <http://www.pices.int/projects/npem/default.aspx> |
| NPPSD | North Pacific Pelagic Seabird Database is a comprehensive database on distribution and abundance of pelagic seabirds off Alaska and in the North Pacific, maintained by the U.S. Geologic Survey and U.S. Fish and Wildlife Service. | <http://www.absc.usgs.gov/research/NPPSD/> |
| CPR | Continuous Plankton Recorder Database contains data from sampling that began in the North Pacific in 2000. The data are archived at the Sir Alister Hardy Foundation for Ocean Science (SAHFOS), an international charity that operates the Continuous Plankton Recorder (CPR) survey. | <http://www.sahfos.ac.uk/data.htm> |
| ArcOD | ArcOD dataset has provided some 120,000 records to OBIS to date, with an additional 50,000 records currently in preparation. | R. Hopcroft, pers. comm., Bluhm et al. 2009; <http://www.arcodiv.org/Database/Data_overview.html> |
| Alaskan seaweeds | Database of specimens of Alaskan seaweeds deposited in herbaria around the world. | Linstrom, 2009; <http://herbarium.botany.ubc.ca/herbarium_data/algae_alaska/search.htm> |
