# Supplementary material for: An Overview of Marine Biodiversity in United States Waters
Source: PLoS One. 2010 Aug 2;5(8):e11914. doi: 10.1371/journal.pone.0011914 (PMC2914028; doi:10.1371/journal.pone.0011914)
Supplement: Table S5 — Assessment of marine biodiversity, represented as number of described species by phylum, in the Southeast U.S. Continental Shelf Large Marine Ecosystem. (0.08 MB DOC) [file pone.0011914.s005.doc]

**Table S5. Assessment of marine biodiversity, represented as number of described species by phylum, in the Southeast U.S. Continental Shelf Large Marine Ecosystem.**

| **Taxon** | **Southeast U.S. Continental Shelf** | **South Atlantic Bight: Cape Hatteras to Cape Canaveral** | **Florida Keys only** |
| --- | --- | --- | --- |
| Bacteria | 32 | 32 |  |
| Cyanophyta/Cyanobacteria | 16 |  |  |
| Ciliophora ◄ |  |  |  |
| Radiolaria |  |  |  |
| Fungi |  |  |  |
| Chlorophyta | 65 | 65 |  |
| Foraminifera | 165 | 165 ‡ |  |
| Bacillariophyta |  |  |  |
| Phaeophyta | 217 | 217 |  |
| Rhodophyta | 38 |  |  |
| Plantae |  |  |  |
| Angiospermae | 10 |  |  |
| Dinoflagellates |  |  |  |
| Porifera | 111 | 111 |  |
| Placozoa | 1 |  |  |
| Cnidaria | 362 | 362 << |  |
| Ctenophora |  |  |  |
| Platyhelminthes |  |  |  |
| Dicyemida/Rhombozoa |  |  |  |
| Orthonectida |  |  |  |
| Nemertea |  |  |  |
| Rotifera |  |  |  |
| Gastrotricha |  |  |  |
| Kinorhyncha |  |  |  |
| Nematoda |  |  |  |
| Nematomorpha |  |  |  |
| Acanthocephala |  |  |  |
| Entoprocta |  |  |  |
| Gnathostomulida |  |  |  |
| Priapulida |  |  |  |
| Loricifera |  |  |  |
| Cycliophora |  |  |  |
| Sipuncula |  |  |  |
| Echiura |  |  |  |
| Annelida | 400 | 400 ■ |  |
| Pogonophora |  |  |  |
| Tardigrada |  |  |  |
| Crustacea | 696 † | 696 |  |
| Chelicerata (nonarachnid) |  |  |  |
| Mollusca | 698 | 696 * | 247 ♫ |
| Phoronida | 2 |  |  |
| Bryozoa/Ectoprocta | 91 | 91 |  |
| Brachiopoda |  |  |  |
| Echinodermata |  |  |  |
| Chaetognatha | 33 | 6 |  |
| Hemichordata |  |  |  |
| Urochordata | 35 |  |  |
| Cephalochordata | 5 |  |  |
| Vertebrata | [1,300] |  |  |
| Pisces | 1,200 | 798 |  |
| Reptilia | 7 |  |  |
| Aves | 73 | 73 ♦ |  |
| Mammalia | 20 |  |  |
| **Totals** | **4,277** |  |  |

NOTES:

Based on species registers, databases, and references from the South Atlantic Bight (Cape Hatteras to Cape Canaveral) and the Florida Keys.

◄ Author estimates that over 80% of species are undescribed.

‡ On the Atlantic continental margin, 876 species were recorded. 165 species of which were found on the Florida-Hatteras slope.

 Represents 52 from inner and middle shelf off Georgia from depths of 13-30 m; 111 is from from [Wenner EL, Hinde P, Knott DM, Van Dolah RF (1984) A temporal and spatial study of invertebrate communities associated with hard-bottom habitats in the South Atlantic Bight. 104 p.]

« 362 spp of Actiniaria, Corallimorpharia, Cubozoa, Schphoza and Hydrozoa (40 species of Actiniaria and Corallimorpharia (Fautin); 4 Cubozoa; 22 Scyphozoa; 296 Hydrozoa exclusive of siphonophores; total of 322 species for these taxa within the Cnidaria).

■ Of the 400 overall Annelida estimate, 377 are polychaetes.

† Author believes this number to be a very conservative estimate.

* Represents 263 species from near Gray's Reef; 696 overall in the SAB.

♫ Represents 247 bivalve species in FL Keys.

♦ Represents 73 species of pelagic birds.
