# Supplementary material for: An Overview of Marine Biodiversity in United States Waters
Source: PLoS One. 2010 Aug 2;5(8):e11914. doi: 10.1371/journal.pone.0011914 (PMC2914028; doi:10.1371/journal.pone.0011914)
Supplement: Table S6 — Compared assessments of large scale/regional marine biodiversity (Insular Pacific-Hawaiian Large Marine Ecosystem vs. worldwide estimate), represented as number of described species by phylum. (0.08 MB DOC) [file pone.0011914.s006.doc]

**Table S6. Compared assessments of large scale/regional marine biodiversity (Insular Pacific-Hawaiian Large Marine Ecosystem vs. worldwide estimate), represented as number of described species by phylum.**

| **Taxon** | **Insular Pacific-Hawaiian LME** | **Estimated Worldwide^a^** |
| --- | --- | --- |
| Bacteria |  | 4,800 |
| Cyanophyta/ Cyanobacteria | 183 | 1,000 |
| Ciliophora |  |  |
| Radiolaria |  | 550 |
| Fungi | 55 | 500 |
| Chlorophyta | 247 | 2,500 |
| Foraminifera | 755 | 10,000 |
| Bacillariophyta | 91 | 5,000 |
| Phaeophyta | 84 | 1,600 |
| Rhodophyta | 574 | 6,200 |
| Plantae |  |  |
| Dinoflagellates | 43 |  |
| Porifera | 144 | 5,500 |
| Placozoa | 1 |  |
| Cnidaria | 460 | 9,795 |
| Ctenophora | 14 | 166 |
| Platyhelminthes | 676 | 15,000 |
| Dicyemida/Rhombozoa |  | 82 |
| Orthonectida |  | 24 |
| Nemertea | 49 | 1,180–1,230 |
| Rotifera | 3 | 50 |
| Gastrotricha | 2 | 390–400 |
| Kinorhyncha | 2 | 130 |
| Nematoda | 54 | 12,000 |
| Nematomorpha | 4 | 5 |
| Acanthocephala | 6 | 600 |
| Entoprocta | 2 | 165–170 |
| Gnathostomulida | 8 | 97 |
| Priapulida | 1 | 8 |
| Loricifera |  | 18 |
| Cycliophora |  | 1 |
| Sipuncula | 14 | 144 |
| Echiura | 6 | 176 |
| Annelida | 343 | 12,000 |
| Pogonophora | 2 | 148 |
| Tardigrada | 2 | 212 |
| Crustacea | 1,325 | 44,950 |
| Chelicerata (non-arachinid) | 19 | 2,267 |
| Mollusca | 1,345 | 52,525 |
| Phoronida | 5 | 10 |
| Bryozoa/Ectoprocta | 168 | 5,700 |
| Brachiopoda | 10 | 550 |
| Echinodermata | 309 | 7,000 |
| Chaetognatha | 18 | 121 |
| Hemichordata | 3 | 106 |
| Urochordata | 102 | 4,900 |
| Cephalochordata | 3 | 32 |
| Vertebrata | [1,295] | [16,585] |
| Pisces | 1,214 | 16,475 |
| Reptilia | 5 |  |
| Aves | 52 |  |
| Mammalia | 24 | 110 |
| **Total** | **8,427** | **224,787** |

NL = Not listed in comparable grouping; UD = Listed in work but number undetermined to date;

[ ] = sum of vertebrate constituent groups.

^a^ Bouchet P (2006) The magnitude of marine biodiversity. In: Duarte CM, editor. The Exploration of Marine Biodiversity Scientific and Technological Challenges. Bilbao: Fundación BBVA. pp. 31–62.
