# Supplementary material for: An Overview of Marine Biodiversity in United States Waters
Source: PLoS One. 2010 Aug 2;5(8):e11914. doi: 10.1371/journal.pone.0011914 (PMC2914028; doi:10.1371/journal.pone.0011914)
Supplement: Table S7 — Alaska regional estimates of marine species, represented as number of described species by phylum (Bruce Wing, NOAA Auke Bay Lab, Juneau). (0.10 MB DOC) [file pone.0011914.s007.doc]

**Table S7. Alaska regional estimates of marine species, represented as number of described species by phylum (Bruce Wing, NOAA Auke Bay Lab, Juneau).**

| **Taxon** | **Alaska (total)** | **Gulf of Alaska *** | **Bering Sea Aleutian Island**** | **Arctic Alaska***** |
| --- | --- | --- | --- | --- |
| Viruses | UD^#^ | P^##^ | P | P |
| Bacteria | UD | P | P | P |
| Cyanophyta/  Cyanobacteria | UD | P | P | P |
| Ciliophora | UD | P | P | P |
| Radiolaria | UD | P | P | P |
| Fungi | UD | P | P | P |
| Chlorophyta | 23 | 23 | 19 | 18 |
| Foraminifera | UD | P | P | P |
| Bacillariophyta | UD | 40 | P | P |
| Phaeophyta | 35 | 35 | 31 | 6 |
| Rhodophyta | 61 | 61 | 51 | 1 |
| Plantae | 3 | 3 | 2 | 0 |
| Dinoflagellates | UD | 25 | P | P |
| Porifera | UD | P | P | P |
| Placozoa | 0 | 0 | 0 | 0 |
| Cnidaria | 150 | 114 | 144 | 56 |
| Ctenophora | 5 | 5 | 4 | 4 |
| Platyhelmenthes | UD | P | P | P |
| Dicyemida/  Rhombozoa | UD | h | h | h |
| Orthonectida | 0 | 0 | 0 | 0 |
| Nemertea | 32 | 29 | 20 | 6 |
| Rotifera | UD | 6 | P | 9 |
| Gastrotricha | UD | P | P | P |
| Kinorhyncha | UD | P | P | P |
| Nematoda | UD | P | P | P |
| Nematomorpha | 0 | 0 | 0 | 0 |
| Acanthocephala | 23 | 13 | 13 | 5 |
| Entoprocta | 0 | 0 | 0 | 0 |
| Gnathostomulida | 0 | 0 | 0 | 0 |
| Priapulida | 1 | 1 | 1 | 0 |
| Loricifera | 0 | 0 | 0 | 0 |
| Cyclophora | 0 | 0 | 0 | 0 |
| Sipunculida | 3 | 3 | 1 | 1 |
| Echiura | 2 | 2 | 2 | 1 |
| Annelida | UD | P | P | P |
| Pogonophora | 1 | 1 | 0 | 0 |
| Tardigrada | UD | P | P | P |
| Crustacea | 700 | 500 | 500 | 250 |
| Chelicerata  (non-arachinid) | UD | 4 | 4 | P |
| Mollusca | 675 | 495 | 494 | 154 |
| Phoronida | 1 | 1 | 1 | 0 |
| Bryozoa/Ectoprocta | UD | P | P | P |
| Brachiopoda | 5 | 5 | 5 | 0 |
| Echinodermata | 94 | 91 | 53 | 12 |
| Chaetognatha | 5 | 4 | 3 | 4 |
| Hemichordata | 0 | 0 | 0 | 0 |
| Urochordata | UD | P | P | P |
| Chephalochordata | 0 | 0 | 0 | 0 |
| Vertebrata | 708 | 542 | 572 | 220 |
| Pisces | 500 | 353 | 385 | 91 |
| Reptilia | 4 | 4 | 1 | 0 |
| Aves | 175 | 162 | 163 | 112 |
| Mammalia | 29 | 23 | 23 | 17 |
| Total | 2527 | 542 | 572 | 220 |

NOTES:

^*^Gulf of Alaska = North of a line from N. Dixon Entrance to SW Kodiak Island.

^**^Bering Sea Aleutian Islands = West of SW Kodiak Island to Bering Strait.

^***^Arctic Alaska = North of Bering Strait to Demarcation Point.

^#^UD= Number Undetermined; ^##^P= Known to be Present.
