# Supplementary material for: An Overview of Marine Biodiversity in United States Waters
Source: PLoS One. 2010 Aug 2;5(8):e11914. doi: 10.1371/journal.pone.0011914 (PMC2914028; doi:10.1371/journal.pone.0011914)
Supplement: Text S1 — Abbreviations from An Overview of Marine Biodiversity in United States Waters. (0.03 MB DOC) [file pone.0011914.s008.doc]

**Text S1. Abbreviations from An Overview of Marine Biodiversity in United States Waters.**

ACIA Arctic Climate Impact Assessment

AFSC Alaska Fisheries Science Center

AIDJEX Arctic Ice Dynamics Joint Experiment

AIS Aquatic Invasive Species

ArcOD Arctic Ocean Diversity

ARLIS Alaskan Resources Library and Information Services

BLM Bureau of Land Management

BoF Bay of Fundy

BSAI Bering Sea and Aleutian Islands

CAFF Conservation of Arctic Flora and Fauna

CALCOFI California Cooperative Oceanic Fisheries Investigations

CAML Census of Antarctic Marine Life

CARMS Canadian Atlantic Register of Marine Species

CeDAMar Census of Diversity of Abyssal Marine Life

CenSeam Global Census of Marine Life on Seamounts

Census Census of Marine Life

ChEss Biogeography of Deep-Water Chemosynthetic Ecosystems

CMarZ Census of Marine Zooplankton

COBEMEX Communidades Bentonicas del Golfo de Mexico

COMARGE Continental Margin Ecosystems on a Worldwide Scale

CPR Continuous Plankton Recorder

CReefs Census of Coral Reef Ecosystems

CSA International Canadian Standards Association International

DOE Department of Energy

DUML Duke University Marine Laboratory

EEZ Exclusive Economic Zone

EMAP Environmental Monitoring and Assessment Program

EORR Experimental Oculinar Research Reserve

EPA Environmental Protection Agency

EVOSTC Exxon Valdez Oil SIPI Trustee Council

FAO Food and Agriculture Organization

FMAP Future of Marine Animal Populations

FMC Fisheries Monitoring Center

GMFMC Gulf of Mexico Fisheries Management Council

GOA Gulf of Alaska

GoM Gulf of Maine

GoMA Gulf of Maine Area program

GOMRMS Gulf of Maine Register of Marine Species

GoMX Gulf of Mexico

HAPC Habitat Area of Particular Concern

HBS Hawaiian Biological Survey

HMAP History of Marine Animal Populations

ICoMM International Census of Marine Microbes

ITIS Integrated Taxonomic Information System

JOCI Joint Ocean Commission Initiative

LME Large Marine Ecosystem

MAFLA Mississippi, Alabama and Florida

MAR-ECO Patterns and Processes of the Ecosystems of the Northern Mid-Atlantic

MARMAP Marine Resources Monitoring, Assessment, and Prediction

MHI Main Hawaiian Islands

MMS Minerals Management Service

MPA Marine Protected Area

NaGISA Natural Geography in Shore Areas

NASA National Aeronautics and Space Administration

NBII National Biological Information Infrastructure

NCA National Coastal Assessment

NCDDC National Coastal Data Development Center

NERR National Estuarine Research Reserve

NMFS National Marine Fisheries Service

NMSP National Marine Sanctuary Program

NOAA National Oceanic and Atmospheric Administration

NOAA ELMR NOAA Estuarine Living Marine Resources

NOAA OE NOAA Office of Exploration

NODC National Oceanographic Data Center

NORPAC North Pacific Studies

NPFMC North Pacific Fishery Management Council

NPRB North Pacific Research Board

NSF National Science Foundation

NWFSC Northwest Fisheries Science Center

NWHI Northwestern Hawaiian Islands

OBIS Ocean Biogeographic Information System

OCSEAP Outer Continental Shelf Environmental Assessment Program

OGMEX Oceanografia del Golfo de Mexico

ONMS Office of National Marine Sanctuaries

PACOOS Pacific Coast Ocean Observing System

PICES North Pacific Marine Science Organization

PISCO Partnership for Interdisciplinary Studies of Coastal Oceans

PMEL Pacific Marine Environmental Laboratory

POST Pacific Ocean Shelf Tracking

PSFMC Pacific States Marine Fisheries Commission

RMS Register of Marine Species

RSMAS Rosenstiel School of Marine and Atmospheric Science

RUSALCA Russian-American Long-term Census of the Arctic

SAB South Atlantic Bight

SAFMC South Atlantic Fishery Management Council

SAHFOS Sir Alister Hardy Foundation for Ocean Science

SBI Shelf-Basin Interactions

SEAMAP Southeast Area Monitoring and Assessment Program

STOCS South Texas Outer Continental Shelf

TCODE Technical Committee on Data Exchange

TINRO Pacific Research and Fisheries Center

TNC The Nature Conservancy

TOPP Tagging of Pacific Predators

TPWD-CFMP Texas Parks and Wildlife Department – Coastal Fisheries Monitoring Program

UAF University of Alaska Fairbanks

U.S. United States

USCOP United States Commission on Ocean Policy

WoRMS World Register of Marine Species
