# Supplementary material for: An Overview of Marine Biodiversity in United States Waters
Source: PLoS One. 2010 Aug 2;5(8):e11914. doi: 10.1371/journal.pone.0011914 (PMC2914028; doi:10.1371/journal.pone.0011914)
Supplement: Text S2 — Taxonomic and Regional Guides for the Northeast U.S. Continental Shelf, Southeast U.S. Continental Shelf, Gulf of Mexico, California Current and Gulf of Alaska, Eastern Bering Sea, and Aleutian Islands, and Chukchi and Beaufort Seas Large Marine Ecosystems. (0.17 MB DOC) [file pone.0011914.s009.doc]

# Text S2. Taxonomic and Regional Guides for the Northeast U.S. Continental Shelf, Southeast U.S. Continental Shelf, Gulf of Mexico, California Current and Gulf of Alaska, Eastern Bering Sea, and Aleutian Islands, and Chukchi and Beaufort Seas Large Marine Ecosystems.

## A. Taxonomic and regional guides to marine organisms of the western North Atlantic, Gulf of Maine to Cape Hatteras

Algae

Sears JR (ed.) (2002) NEAS keys to benthic marine algae of the Northeastern Coast of North America from Long Island Sound to the Strait of Belle Isle. Second edition. Northeast Algal Society, Dartmouth, Mass. 161 pp.

South GR, Tittley I (1986) A checklist and distributional index of the benthic marine algae of the North Atlantic Ocean. Huntsman Marine Laboratory. St. Andrews, New Brunswick. 76 pp.

Taylor WR (1957) Marine algae of the northeastern coast of North America. The University of Michigan Press. 509 pp.

Vascular plants

Silberhorn GM (1999) Common plants of the Mid-Atlantic coast: A field guide. Second edition. Johns Hopkins University Press. 294 pp.

Tiner RW Jr (1987) A field guide to coastal wetland plants of the Northeastern United States. University of Massachusetts Press. 285 pp.

Phytoplankton

Gowen AW, Mulligan HF (1978) A photographic guide to phytoplankton of the coastal waters, Gulf of Maine. Published by Shoals Marine Laboratory, Cornell University. 164 pp.

Marshall HG (1986) Identification manual for phytoplankton of the United States Atlantic coast. U.S. Environmental Protection Agency Publication EPA/600/4-86/003. 142 pp.

Zooplankton

Gerber RP (2000) An identification manual to the coastal and estuarine zooplankton of the Gulf of Maine region from Passamaquoddy Bay to Long Island Sound. Part I. Text and identification keys. Freeport Village Press. 80 pp.

Johnson WS, Allen DM (2005) Zooplankton of the Atlantic and Gulf coasts: A guide to their identification and ecology. JHU Press. 379 pp.

Mollusks

Morris PA (1973) Field guide to shells of the Atlantic and Gulf coasts. Revised edition. Peterson Field Guide Series, No. 3. Boston: Houghton Mifflin. 330 pp. (includes some deep-sea species that were excluded from later editions)

Turgeon DD, Quinn JF Jr., Bogan AE, Coan EV, Hochberg FG, et al. (1998) Common and scientific names of aquatic invertebrates from the United States and Canada: Mollusks. Second Edition. American Fisheries Society Special. Publication 26. 526 pp.

Vecchione M, Roper CFE, Sweeney MJ (1989) Marine flora and fauna of the eastern United States. Mollusca: Cephalopoda. NOAA Technical Report. NMFS 73. 23 pp.

Vecchione M, Roper CFE, Sweeney MJ, Lu CC (2001) Distribution, relative abundance, and developmental morphology of paralarval cephalopods in the western North Atlantic Ocean. NOAA Tech Rep. NMFS 152. 54 pp.

Crustaceans

Murphy JA, Cohen RE (1978) A guide to the developmental stages of common coastal, Georges Bank and Gulf of Maine copepods. National Marine Fisheries Service, Northeast Fisheries Center. Woods Hole Laboratory, Massachusetts. Woods Hole laboratory reference document 78-53. 56 pp.

Pohle GW (1988) A guide to the deep-sea shrimp and shrimp-like decapod Crustacea of Atlantic Canada. Canadian Technical Report of Fisheries and Aquatic Science 1651, 29 pp.

Pohle GW (1990) A guide to decapod Crustacea from the Canadian Atlantic: Anomura and Brachyura. Canadian Technical Report of Fisheries and Aquatic Science 1771) 29 pp.

Roff JC (1978) A guide to the marine flora and fauna of the Bay of Fundy: Copepoda: Calanoida. Fisheries and Marine Service Technical Report 823. 27 pp.

Williams AB (1984) Shrimps, lobsters, and crabs of the Atlantic coast of the eastern United States, Maine to Florida. Washington, D.C.: Smithsonian Institution Press. 550 pp.

Other invertebrates

Amin OM (1998) Marine flora and fauna of the eastern United States: Acanthocephala. NOAA Technical Report NMFS 135. U.S. Department of Commerce. 27 pp.

Appy TD, Linkletter LE, Dadswell MJ (1980) A guide to the marine flora and fauna of the Bay of Fundy: Annelida: Polychaeta. Fisheries & Marine Service Technical Report No. 920. 124 pp.

Bleakney JS (1996) Sea slugs of Atlantic Canada and the Gulf of Maine. The Nova Scotia Museum Field Guide Series. Nimbus Publishing. Halifax. 216 pp.

Cutler EB (1977) Marine flora and fauna of northeastern United States. Sipuncula. NOAA Tech. Rep. NMFS Circular 403. 7 pp.

Hendrix SS (1994) Marine flora and fauna of the eastern United States. Platyhelminthes: Monogena. NOAA Technical Report NMFS 121. 107 pp.

Larson RJ (1976) Marine flora and fauna of the northeastern United States. Cnidaria: Scyphozoa. NOAA Techical Report NMFS Circular 397. 18 pp.

Ryland JS, Hayward PJ (1991) Marine flora and fauna of the northeastern United States: Erect Bryozoa. NOAA Technical Report NMFS 99. 48 pp.

Sebens KP (1998) Marine flora and fauna of the eastern United States. Anthozoa, Actinaria, Corallimorparia, Ceriantharia, and Zoanthidea. NOAA Technical Report NMFS 141. 68 pp.

Fish

Colette BB, Klein-MacPhee G (eds.) (2002) Bigelow and Schroeder’s fishes of the Gulf of Maine. Third edition. Smithsonian Institution Press. 748 pp.

Fahay MP (1983) Guide to the early stages of marine fishes occurring in the western North Atlantic Ocean, Cape Hatteras to the southern Scotian Shelf. Journal of NW Atlantic Fishery Science, Vol. 4, 423 pp. (B&W illustrations)

Fritzsche RA (1978) Development of fishes of the Mid-Atlantic Bight: An atlas of egg, larval and juvenile stages. Volume V. Chaetodontidae through Ophidiidae. U.S. Fish and Wildlife Service, Biological Services Program FWS/OBS-78/12. 340 pp.

Garrick JAF (1982) Sharks of the genus Carcharhinus. NOAA Technical Report NMFS Circular 445. 194 pp.

Hardy JD Jr (1978) Development of fishes of the Mid-Atlantic Bight. An atlas of egg, larval and juvenile stages. Volume II. Anguillidae through Syngnathidae. U.S. Fish and Wildlife Service, Biological Services Program FWS/OBS-78/12. 458 pp.

Hardy JD Jr (1978) Development of fishes of the Mid-Atlantic Bight: An atlas of egg, larval and juvenile stages. Volume III. Aphredoderidae through Rachycentridae. U.S. Fish and Wildlife Service, Biological Services Program FWS-OBS-78/12. 394 pp.

Hildebrand SF, Schroeder WC (1972) Fishes of Chesapeake Bay. T.F.H. Publications, Inc. 388 pp.

Johnson GD (1978) Development of fishes of the Mid-Atlantic Bight: An atlas of egg, larval and juvenile stages. Volume IV. Carangidae through Ephippidae. U.S. Fish and Wildlife Service, Biological Services Program FWS/OBS-78/12. 314 pp.

Jones PW, Martin FD, Hardy JD Jr. (1978) Development of fishes of the Mid-Atlantic Bight. An atlas of egg, larval and juvenile stages. Volume I. Acipenseridae through Ictaluridae. U.S. Fish and Wildlife Service, Biological Services Program FWS/OBS-78/12. 366 pp.

Leim AH, Scott WB (1966) Fishes of the Atlantic coast of Canada. Fisheries Research Board of Canada Bulletin No. 155. 485 pp.

Martin FD, Drewry GE (1978) Development of fishes of the Mid-Atlantic Bight: An atlas of egg, larval and juvenile stages. Volume VI. Stromateidae through Ogcocephalidae. U.S. Fish and Wildlife Service, Biological Services Program FWS/OBS-78/12. 416 pp.

Robins CR, Ray GC (1986) A field guide to Atlantic Coast fishes of North America. Peterson Field Guide Series. Houghton Mifflin Co., 354 pp.

Marine mammals and turtles

Hannah J (1998) Seals of Atlantic Canada and the northeastern United States. International Marine Mammal Association. 33 pp.

Katona SK, Rough V, Richardson DT (1993) A field guide to whales porpoises and seals from Cape Cod to Newfoundland. Fourth Edition. Smithsonian Institution Press. 316 pp.

Leatherwood SL, Caldwell DK, Winn HE, with special assistance from WE Schevill and MC Caldwell (1976) Whales, dolphins and porpoises of the western North Atlantic: A guide to their identification. NOAA Technical Report NMFS CIRC-396. 176 pp.

Wynne K, Schwartz M (1999) Guide to marine mammals and turtles of the U.S. Atlantic & Gulf of Mexico. Rhode Island Sea Grant, 115 pp.

Birds

Harrison P (1991) Seabirds: An identification guide. Houghton Mifflin Harcourt. 448 pp.

Peterson RT, [Peterson](http://www.amazon.com/exec/obidos/search-handle-url/ref=ntt_athr_dp_sr_2?_encoding=UTF8&search-type=ss&index=books&field-author=Virginia Marie Peterson) VM (2002) A field guide to the birds of eastern and central North America. Fifth edition. Houghton-Mifflin Books, 450 pp.

Sibley DA (2003) The Sibley field guide to birds of eastern North America. Alfred A. Knopf. 431 pp.

General field guides

Gosner KL (1978) A field guide to the Atlantic seashore from the Bay of Fundy to Cape Hatteras. Boston: Houghton Mifflin. 329 pp. (color and B&W illustrations; includes seaweeds)

Martinez AJ (1999) Marine Life of the North Atlantic: Canada to New England. 2nd Ed. Down East Books. 272 pp. (color photos)

Pollock LW (1998) A practical guide to the marine animals of northeastern North America. Rutgers University Press. 367 pp. (B&W illustrations)

Proctor NW, Lynch PJ (2005) A field guide to North Atlantic wildlife: Marine mammals, seabirds, fish and other sea life. Yale University. 256 pp. (color illustrations)

Shumway SW (2008) The naturalist’s guide to the Atlantic seashore: Beach ecology from the Gulf of Maine to Cape Hatteras. Falcon Guides, an imprint of Globe Pequot Press. 232 pp. (color photos and illustrations)

[Watling](http://www.amazon.com/exec/obidos/search-handle-url/ref=ntt_athr_dp_sr_1?_encoding=UTF8&search-type=ss&index=books&field-author=Les Watling) L, [Fegley](http://www.amazon.com/exec/obidos/search-handle-url/ref=ntt_athr_dp_sr_3?_encoding=UTF8&search-type=ss&index=books&field-author=Jill Fegley) J, [Moring](http://www.amazon.com/exec/obidos/search-handle-url/ref=ntt_athr_dp_sr_5?_encoding=UTF8&search-type=ss&index=books&field-author=John Moring) J (2003) Life between the tides: marine plants and animals of the northeast. Tilbury House Publishers. 110 pp. (B&W illustrations)

Ward N (1995) Stellwagen Bank: A guide to the whales, sea birds, and marine life of the Stellwagen Bank National Marine Sanctuary. Down East Books. 232 pp. (color photos and illustrations).

Zinn DJ (1985) The beach strollers handbook, from Maine to Cape Hatteras. 2nd ed. Chester, Conn: Globe Pequot, 246 pp. (B&W illustrations)

## B. Taxonomic and regional identification guides to marine organisms of the Southeast U.S. Continental Shelf LME

Algae

Littler DS, Littler MM (2000) Caribbean reef plants. Washington, D.C.: Offshore Graphics, Inc. 542 pp.

Searles RB (1981) Seaweeds from Gray's Reef, Georgia. *Northeast Gulf Science* 5, (1): 45-48.

Searles RB (1987) Phenology and floristics of seaweeds from the offshore waters of Georgia. *Northeast Gulf Sci* 9 (2): 99-108.

Cnidaria

Sebens KP (1998) Marine flora and fauna of the eastern United States Anthozoa: Acitniaria, Corallimorpharia, Ceriantharia, and Zoanthidea. NOAA Technical Report NMFS 141. 68 pp.

Smith FGW (1971) Atlantic reef corals; A handbook of the common reef and shallow-water corals of Bermuda, the Bahamas, Florida, the West Indies, and Brazil. Coral Gables, Fla.: University of Miami Press.

Arthropoda

Heard RW, King RA, Knott DM, Thoma BP, Thornton-DeVictor S (2007) A guide to the Thalassinidea (Crustacea: Malacostraca: Decapoda) of the South Atlantic Bight. NOAA Professional Paper NMFS 8. 30 pp.

Heard RW, Price WW, Knott DM, King RA, Allen DM (2006) A taxonomic guide to the mysids of the South Atlantic Bight. NOAA Professional Paper NMFS 4. 37 pp.

McCain JC (1968) The Caprellidae (Crustacea: Amphipoda) of the western North Atlantic. Washington, D.C.: Smithsonian Institution Press.

Williams AB (1984) Shrimps, lobsters, and crabs of the Atlantic Coast of the eastern United States, Maine to Florida. Washington, D.C.: Smithsonian Institution Press.

Polychaeta

Day JH (1973) New polychaeta from Beaufort, with a key to all species recorded from North Carolina. NOAA Tech. Rept. NMFS Circ. 375.

Mollusca

Mikkelsen PM, Bieler R (2008) Seashells of southern Florida: Living marine mollusks of the Florida Keys and adjacent regions. Bivalves. Princeton, N.J.: Princeton University Press. 503 pp.

Vecchione M, Roper CFE, Sweeney MJ (1989) Marine Flora and Fauna of the Eastern United States Mollusca: Cephalopoda. NOAA Technical Report NMFS 73. 23 pp.

Echinodermata

Hendler G, Miller JE, Pawson DL, Kier PM (1995) Sea stars, sea urchins, and allies: Echinoderms of Florida and the Caribbean. Washington, D.C.: Smithsonian Institution Press. 390 pp.

Vertebrata

Carpenter KE (ed.) (2002) The living marine resources of the western Central Atlantic. Three volumes. FAO Species Identification Guide for Fishery Purposes and American Society of Ichthyologists and Herpetologist Special Publication No. 5. Rome, FAO. 2127 pp.

Chao LN (1978) A basis for classifying western Atlantic Sciaenidae (Teleostei: Perciformes). NOAA Tech. Rep. NMFS Circ. 415. 64 pp.

Poss SG, Collette BB (1995) Second survey of fish collections in the United States and Canada. Copeia 1995(1): 48-70.

Richards WJ (2006) Early stages of Atlantic fishes: An identification guide for the western central North Atlantic. Boca Raton, Fla.: Taylor and Francis, 2 v. 2640 pp.

Springer S (1979) A revision of the catsharks, family Scyliorhinidae. NOAA Tech. Rep. NMFS Circ. 422. 152pp.

## C. Taxonomic and regional identification guides to marine organisms of the Gulf of Mexico LME

Coral Reefs

Kaplan E, Peterson TR, Kaplan, S (1999) A Field Guide to Coral Reefs: Caribbean and Florida.

First Edition. Houghton Mifflin Harcourt.

Spalding M, Bunting G, Ravilious, C (2004) A Guide to the Coral Reefs of the Caribbean. First Edition. University of California Press.

Cnidarians (miscellaneous)

Cairns S (1976) Guide to the commoner shallow-water gorgonians (sea whips, sea feathers, and sea fans) of Florida, the Gulf of Mexico, and the Caribbean region. Miami, Fla.: Sea Grant Field Guide Series. University of Miami Sea Grant Program.

Crustaceans

Heard RW (1982) Guide to common tidal marsh invertebrates of the northeastern Gulf of Mexico. Mississippi-Alabama Sea Grant Consortium. No. MASGP-79-004. 82 pp.

Williams A (1965) Decapod Crustaceans of the Carolinas. U.S. Deptarment of the Interior, Fish and Wildlife Service, Bureau of Commercial Fisheries. Washington, D.C.: U.S. Government Printing Office. [For sale by the Superintendent of Documents]

Fish

Bohlke JE, Chaplin CG (1993) Fishes of the Bahamas and Adjacent Tropical Waters. Second Edition. University of Texas Press.

Dahlberg MD (2008) Guide to the Coastal Fishes of Georgia and Nearby States. University of Georgia Press.

Dunaway V, Brant KR (2000) Sport Fish of the Gulf of Mexico. Wickstrom Publishers.

Greenberg I, Greenberg J (1986) Waterproof Guide to the Corals and Fishes of Florida, the Bahamas and the Caribbean. Nu Novelties.

Hoese H, Moore R (1998) Fishes of the Gulf of Mexico,Texas, Louisiana, and Adjacent Waters. Second Edition. Texas A&M University Press.

Horst J, Lane M, Raver D (2006) Angler's Guide to the Fishes of the Gulf of Mexico. Illustrated Edition. Pelican Publishing Company.

Humann P, DeLoach N (2002) The Reef Set: Reef Fish, Reef Creatures and Reef Coral (3 Volumes). Second Edition. New World Publications.

McEachran JD, Fechhelm JD (2006) Fishes of the Gulf of Mexico. University of Texas Press.

McKee DA, Compton H, Fechhelm JD (2008) Fishes of the Texas Laguna Madre: A Guide for Anglers and Naturalists. Texas A&M University Press.

Parsons G (2006) Sharks, Skates, and Rays of the Gulf of Mexico. University Press of Mississippi.

Randall JE (1996) Caribbean Reef Fishes. Third Revised Edition. TFH Publications.

Schwartz FJ (1984) Sharks, Sawfish, Skates and Rays of the Carolinas. Morehead City, North Carolina: Institute of Marine Sciences.

Smith CL, National Audubon Society (2000) National Audubon Society Field Guide to Tropical Marine Fishes: Of the Caribbean, the Gulf of Mexico, Florida, the Bahamas, and Bermuda. Scholastic.

Stokes FJ (1980) Handguide to the Coral Reef Fishes of the Caribbean. Lippincott and Crowell.

Tobbins CR, Ray GC, Douglas J (1999) A Field Guide to Atlantic Coast Fishes of North America. Houghton Mifflin Harcourt.

Flora

Dawes CJ (1990) Marine Algae of the West Coast of Florida. Miami, Fla.: University of Miami Press.

Hanlon R (1975) Guide to the mangroves, buttonwood, and poisonous shoreline trees of Florida, the Gulf of Mexico, and the Caribbean region. Sea Grant Field Guide Series. Miami, Fla.: University of Miami Sea Grant Program.

Hanlon R (1975) Guide to the sea grasses of Florida, the Gulf of Mexico, and the Caribbean region. Sea Grant Field Guide Series. Miami, Fla.: University of Miami Sea Grant Program.

Littler DS, Littler MM (2000) Caribbean Reef Plants: an identification guide to the reef plants of the Caribbean, Bahamas, Florida, and Gulf of Mexico. First Edition. OffShore Graphics, Inc.

Littler DS, Littler MM, Bucher KE, Norris JN (1989). Marine Plants of the Caribbean: A Field Guide from Florida to Brazil. First Edition. Smithsonian.

Taylor WR (1967) Marine Algae of the Eastern Tropical and Subtropical Coasts of the Americas. University of Michigan Press.

Echinoderms

Hess SC (1978) Guide to the commoner shallow-water asteroids (starfish) of Florida, the Gulf of Mexico, and the Caribbean region. Sea Grant Field Guide Series. Miami, Fla.: University of Miami Sea Grant Program.

Phelan TF (1970) A field guide to the Cidaroid echinoids of the northwestern Atlantic Ocean, Gulf of Mexico, and the Caribbean Sea. Smithsonian Contributions to Zoology No. 40. Washington, D.C.: Smithsonian Institution Press. [For sale by the Superintendent of Documents, U.S. Government Printing Office].

Marine Mammals

Wursig BG, Jefferson TA (2000) The Marine Mammals of the Gulf of Mexico. First Edition. Texas A&M University Press.

Wynne K, Schwartz M, Mix G (1999) Guide to Marine Mammals & Turtles of the U.S. Atlantic & Gulf of Mexico. Alaska Sea Grant College Program.

Mollusks

Abbott T (1974) American Seashells; The Marine Molluska of the Atlantic and Pacific Coasts of North America. Second Edition. (Hardcover). Van Nostrand Reinhold.

Abbott TR, Zim HS, Sandstrom G (2001) Seashells of North America: A Guide to Field Identification. Golden Guides from St. Martin's Press.

Andrews J (1994) Field Guide to Shells of the Florida Coast. Taylor Trade Publishing.

Andrews J (1977) Shells and Shores of Texas. First Edition. University of Texas Press.

Hartmann T (2007) Bivalve Seashells of Florida. First Edition. Anadara Press.

Mikkelsen P, Bieler R (2007) Seashells of Southern Florida: Living Marine Mollusks of the Florida Keys and Adjacent Regions: Bivalves. Vol. 1. Illustrated Edition. Princeton University Press.

Morris PA (1952) Shells of the Atlantic and Gulf coasts and the West Indies. Roger Tory Peterson Field Guides. Houghton Mufflin.

Morris VF, Abbott RT, Peterson RT (1995) A Field Guide to Shells of the Atlantic and Gulf Coasts and the West Indies. Fourth Edition. The Peterson Field Guide Series. Houghton Mifflin Harcourt.

Opresko L (1992) A guide to the larger marine gastropods of Florida, the Gulf of Mexico, and the Caribbean region. Sea Grant Field Guide Series. Miami, Fla.: University of Miami Sea Grant Program.

Romashko S (1992) The Shell Book: Florida, Gulf, and the Caribbean. Sixth Edition. Winward Publishing Inc.

Witherington B, Witherington D (2007) Florida’s Seashells. Pineapple Press.

Plankton

Curl HC (1953) The phytoplankton of Apalachee Bay and the Northeastern Gulf of Mexico (Contribution / Oceanographic Institute, Florida State University). Florida State University.

Johnson WS, Allen DM (2005) Zooplankton of the Atlantic and Gulf Coasts: A Guide to Their Identification and Ecology. Illustrated Edition. The Johns Hopkins University Press.

Pierce R (1979) Phytoplankton of the Gulf of Mexico: Taxonomy of calcareous nannoplankton (Geoscience and man). School of Geoscience, Louisiana State University.

Polychaetes

Johnson P, Uebelacker J (1984) Taxonomic Guide to the Polychaetes of the Northern Gulf of Mexico. Volumes I through VII. Barry A. Vittor & Associates.

Seashores

Kaplan EH, Peterson RT, Kaplan, SL (1999) Southeastern & Caribbean Seashores. Second Edition. Houghton Mifflin Harcourt.

## D. Taxonomic and regional identification guides to marine organisms of the California Current LME

Ciliophora, Other Protozoa

Love MS, Moser M (1983) A checklist of parasites of California, Oregon, and Washington marine and estuarine fishes. NOAA Tech. Rep. NMFS SSRF-777.

Plantae

Lamb A, Hanby, BP (2005) Marine Life of the Pacific Northwest - A Photographic Encyclopedia of Invertebrates, Seaweeds and Selected Fishes. Harbour Publishing, Madeira Park, BC. 398pp.

Porifera

Austin WC (1985) An Annotated Checklist of Marine Invertebrates in the Cold Temperate Northeast Pacific. Vol. 1: 21-42. Khoyatan Marine Laboratory: Cowichan Bay, BC.

Placozoa, Ctenophora, Dicyemida, Orthonectida, Rotifera, Nematoda, Nematomorpha, Entoprocta, Gnathostomulida, Priapulida, Sipuncula, Echiura, Tardigrada, Phoronida, Bryozoa, Brachiopoda, Urochordata, Cephalochordata

Carlton JT (ed.) (2007) The Light and Smith Manual: Intertidal Invertebrates from Central California to Oregon. Fourth Edition, Completely Revised and Expanded. Berkeley, California: University of California Press.

Platyhelminthes

Carlton JT (ed.) (2007) The Light and Smith Manual: Intertidal Invertebrates from Central California to Oregon. Fourth Edition, Completely Revised and Expanded. Berkeley, California: University of California.

Austin WC (1985) An Annotated Checklist of Marine Invertebrates in the Cold Temperate Northeast Pacific. Vol. 1: 21-42. Khoyatan Marine Laboratory: Cowichan Bay, BC.

Ching HL (1991) Lists of larval worms from marine invertebrates of the Pacific Coast of North America. J. Helminthological Society of Washington. 58:57-68.

Love MS, Moser M (1983) A checklist of parasites of California, Oregon, and Washington marine and estuarine fishes. NOAA Tech. Rep. NMFS SSRF-777.

Nemertea

Crandall FB, Norenburg JL (2001) Checklist of the Nemertean Fauna of the United States. 2nd ed. pp. 1-36.

Roe P, Norenburg JL, Maslakova SA (2007) Nemertea. In: The Light and Smith Manual: Intertidal Invertebrates from Central California to Oregon, 4th Edition. J. Carlton (ed). pp 182-196.

Gastrotricha, Kinorhyncha, Loricifera, Mollusca

Carlton JT (ed.) (2007) The Light and Smith Manual: Intertidal Invertebrates from Central California to Oregon. Fourth Edition, Completely Revised and Expanded. Berkeley, California: University of California.

Kozloff EN (1996) Marine invertebrates of the Pacific Northwest. Seattle: University of Washington Press.

Acanthocephala

Carlton JT (ed.) (2007) The Light and Smith Manual: Intertidal Invertebrates from Central California to Oregon. Fourth Edition, Completely Revised and Expanded. Berkeley, California: University of California.

Love MS, Moser M (1983) A checklist of parasites of California, Oregon, and Washington marine and estuarine fishes. NOAA Tech. Rep. NMFS SSRF-777.

Chaetognatha

<http://academic.evergreen.edu/t/thuesene/>

Hemichordata

Carlton JT (ed.) (2007) The Light and Smith Manual: Intertidal Invertebrates from Central California to Oregon. Fourth Edition, Completely Revised and Expanded. Berkeley, California: University of California.

Holland ND, Clague DA, Gordon DP, Gebruk A, Pawson DL, Vecchione M (2005) 'Lophenteropneust' hypothesis refuted by collection and photos of new deep-sea hemichordates. Nature 434:374-376.

Woodcock KH (1996) Phylum Hemichordata, Class Enteropneusta. In Taxanomic atlas of the benthic fauna of the Santa Maria Basin and Western Santa Barbara Channel. vol. 14. J.A. Blake, P.H. Scott and A. Lissner (eds). Santa Barbara, CA: Santa Barbara Museum of Natural History, pp. 251-259

Aves

Harrison P (1983) Seabirds, An Identification Guide. Boston: Houghton Mifflin.

Mammalia

Jefferson TA, Webber MA, Pitman RL (2008) Marine mammals of the world: a comprehensive guide to their identification. London; Burlington, MA: Academic.

## E. Taxonomic and regional identification guides to marine organisms of the Alaska, Eastern Bering Sea and Aleutian Islands, and Chukchi and Beaufort Seas LMEs

Gulf of Alaska

##### Entry points into the broader scientific literature

Hood D, Zimmerman S (eds.) (1986) Gulf of Alaska, physical environment and biological resources. Washington, D.C.: NOAA Ocean Assessments Division, Alaska Office. 655 p.

Mundy P (ed.) (2005) The Gulf of Alaska: Biology and Oceanography. Alaska Sea Grant College Program, University of Alaska Fairbanks. 214 p

NPRB (2005) North Pacific Research Board science plan. North Pacific Research Board. 198 p

PICES (2004) Marine ecosystems of the North Pacific. PICES (North Pacific Marine Science Organization) Special Publication 1. 280 p.

Spies R (ed.) 2007) Long-term ecological change in the northern Gulf of Alaska: Elsevier. 589 p.

Boeing W, Duffy-Anderson J (2008) Ichthyoplankton dynamics and biodiversity in the Gulf of Alaska: Responses to environmental change. Ecol. Indic., 8 (3), pp. 292-302.

Examples of taxonomic studies and species/taxa lists

ADFG (1976) Marine plant community studies. Final report. Kachemak Bay, Alaska: Alaska Dept. of Fish and Game. 288 p.

Atlas RM, Griffith RP (1986) Chapter 8. Microbiology. In: Hood D, Zimmerman S editors. Gulf of Alaska, physical environment and biological resources. Washington, D.C.: NOAA Ocean Assessments Division, Alaska Office pp. 221-246.

Austin WC (1985) An annotated checklist of marine invertebrates in the cold temperate northeastern Pacific. Vol. 1. Cowichan Bay, BC: Khoyatan Marine Laboratory. 218 p.

Baxter R (1983) Mollusks of Alaska. Alaska Dept of Fish and Game. 77 p.

Calkins DG (1986) Chapter 17. Marine Mammals. In: Hood D, Zimmerman S, editors. Gulf of Alaska, physical environment and biological resources. NOAA Ocean Assessments Division, Alaska Office, Washington, D.C. pp. 527-558.

Coe WR (1910) Nemertians. Harriman Alaska Series Vol. XI: Smithsonian Institution. pp. 251.

Cooney RT (1981) Bering Sea zooplankton and micronekton communities with emphasis on annual production. In: Hood DW, Calder JA, editors. The eastern Bering Sea shelf: Oceanography and Resources Vol. 2. Seattle: University of Washington Press. pp. 947-974.

Cuffey RJ (1983) Modern Bryozoans on the Kodiak shelf off southern Alaska. Scientific report for U.S. Minerals Management Service. 196 p.

Davis CC (1949) The pelagic Copepoda of the Northeastern Pacific Coast. University of Washington Press. UW Publications in Biology 14: 1-118.

DeGange AR, Sanger GA (1986) Chapter 16. Marine birds. In: Hood D, Zimmerman S editors. Gulf of Alaska, physical environment and biological resources. NOAA Ocean Assessments Division, Alaska Office, Washington, D.C. pp. 479-525.

Dick MH, Ross JRP (1988) Intertidal Bryozoa (Cheilostomata) of the Kodiak vicinity, Alaska. Occasional Paper 23. Center for Pacific Northwest Studies. Western Washington

Feder HM, Jewett SC (1986) Chapter 12. The subtidal benthos. In: Hood D, Zimmerman S editors. Gulf of Alaska: Physical environment and biological resources. Washington, D.C.: NOAA Ocean Assessments Division, Alaska Office. pp. 347-396.

Foster NR (1991) Intertidal bivalves. A guide to the common marine bivalves of Alaska. University of Alaska Press. 152 p.

Foster NR (2003) Database on the marine invertebrate macrofauna of Prince William Sound: An addition to the University of Alaska Museum’s ARCTOS Network. Exxon Valdez Oil Spill Gulf of Alaska Monitoring and Research Project 030642 Final Report.

Foster NR, Hoberg MK (2003) Permanent archiving of specimens collected in nearshore habitats. Exxon Valdez Oil Spill Restoration Project (#02628) Final Report. University of Alaska Fairbanks.

Lindstrom SC (2006) Biogeography of Alaskan seaweeds. J App Phycol 18: 637-641.

Lindstrom SC (2009) The biogeography of seaweeds in Southeast Alaska. J Biogeogr 36: 401-409.

Robertson A (1910) Bryozoans. Harriman Alaska Series Vol. XI: Smithsonian Institution. pp. 251.

Rogers DE, Rodgers BJ, Rosenthal RJ (1986) Chapter 13. The nearshore fishes. In: Hood D, Zimmerman S editors. 1986) Gulf of Alaska, physical environment and biological resources. NOAA Ocean Assessments Division, Alaska Office, Washington, D.C. pp. 399-415

Sambrotto RN, Lorenzen CJ (1986) Chapter 9. Phytoplankton and primary production. In: Hood D, Zimmerman S, editors. Gulf of Alaska, physical environment and biological resources. Washington, D.C.: NOAA Ocean Assessments Division, Alaska Office. pp. 249-282.

Bering Sea and Aleutians

Entry points into the broader scientific literature

Coyle KO (2005) Zooplankton distribution, abundance and biomass relative to water masses in eastern and central Aleutian Island passes. Fisheries Oceanography 14: 77-92.

Dagg M, Royer TC, Macklin SA (eds.) (2002) Ecology of the southeastern Bering Sea. Deep-Sea Res II 49: 5811-6168.

Hood DW, Calder JA (eds.) (1981) The eastern Bering Sea shelf: Oceanography and Resources. Vol. 2. Seattle: University of Washington Press.

Macklin SA, Saito SI, Radchenko VI, Napp JM, Stabeno PJ, et al. (2002) Variability in the Bering Sea Ecosystem. Prog Oceanogr 55: 261 p.

Macklin SA, Schumacher J, Moore SE, Smith S (2008) Sustaining the marine ecosystem of the Pribilof Domain. Deep Sea Res. II (Top. Stud. Oceanogr.). Vol. 55, no. 16-17, pp. 1698-1700.

Macklin SA, Hunt GL Jr (eds.) 2004) The Southeast Bering sea ecosystem: Implications for marine resource management. Final Report: Southeast Bering Sea Carrying Capacity. Silver Spring, Md.: NOAA Coastal Ocean Program Decision Analysis Series No. 24. 192 p.

Mathisen OA, Coyle KO (eds.) (1996) Ecology of the BeringSea: A review of Russian literature. University of Alaska Sea, Grant, AK-SG-96-01, Fairbanks, 306pp.

Mueter FJ, Litzow MA (2008) Sea ice retreat alters the biogeography of the Bering Sea continental shelf. Ecol Appl 18: 309-320.

NRC, National Research Council (1996) The Bering Sea ecosystem. Washington, D.C.: National Academy Press. 307 p.

NPRB, North Pacific Research Board (2005) North Pacific Research Board science plan. Anchorage, Alaska: NPRB. 198 p

PICES, North Pacific Marine Science Organization (2004) Marine ecosystems of the North Pacific. PICES Special Publication 1. 280 p.

Schumacher JD, Kruse GH, Macklin SA (eds.) 2005) The Aleutian ecosystem: Processes controlling variability in productivity and ecosystem structure. Fisheries Oceanography 14: 306.

Examples of taxonomic studies and species/taxa lists

Allen MJ, Smith GB (1988) Atlas and zoogeography of common fishes in the Bering Sea and northeastern Pacific. NOAA Technical Report NMFS 66: 115-133.

Brodsky KA (1950) Calanoida of the far eastern seas and Polar Basin of the USSR. USSR Academy of Sciences No. 35. (Translated from Russian by Israel Program for Scientific Translations. 1967).

Burns JJ, Shapiro LH, Fay FH (1981) Ice as marine mammal habitat in the Bering Sea. In: Hood DW, Calder JA, editors. The eastern Bering Sea shelf: Oceanography and resources. Vol. 2. Seattle: University of Washington Press. pp. 781-797.

Cooney RT (1981) Bering Sea zooplankton and micronekton communities with emphasis on annual production. In: Hood DW, Calder JA, editors. The eastern Bering Sea shelf: Oceanography and resources Vol. 2. Seattle: University of Washington Press. pp. 947-974.

Coyle KO, Chavtur VG, Pinchuk AI (1996) Zooplankton of the Bering Sea: A review of Russian-language literature. In: Mathisen OA, Coyle KO, editors. Ecology of the Bering Sea A review of Russian literature Alaska Sea Grant College Program Report No 96-01. Fairbanks: University of Alaska. pp. 97-133.

Coyle KO, Pinchuk AI, Eisner LB, Napp JM (2008) Zooplankton species composition, abundance and biomass on the eastern Bering Sea shelf during summer: The potential role of water-column stability and nutrients in structuring the zooplankton community. Deep-Sea Res II 55: 1775-1791.

D’yakonov AM (1923) Fauna of Russia and adjacent countries. Vol. 1: Echinodermata, Echinoidea. (Translated from Russian by Israel Program for Scientific Translations, Jerusalem 1969) 265 p.

Gibson DD, Byrd GV (2007) Birds of the Aleutian Islands. Series in ornithology. 1. Fayetteville, Ark.: American Ornithologists’ Union. 367 p.

Gill RE, Handel CH (1981) Shorebirds of the eastern Bering Sea. In: Hood DW, Calder JA, editors. The eastern Bering Sea shelf: Oceanography and Resources Vol. 2. Seattle: University of Washington Press. pp. 719-738.

Hunt GL Jr., Gould PJ, Forsell DJ, Peterson H Jr. (1981) Pelagic distribution of marine birds in the eastern Bering Sea. In: Hood DW, Calder JA, editors. The eastern Bering Sea shelf: Oceanography and Resources Vol. 2. Seattle: University of Washington Press. pp. 689-718.

Jewett SC, Feder HM (1981) Epifaunal invertebrates of the continental shelf of the eastern Bering Sea and Chukchi Seas. In: Hood DW, Calder JA, editors. The eastern Bering Sea shelf: Oceanography and Resources Vol. 2. Seattle: University of Washington Press. pp. 1131-1153.

MacIntosh RA (1976) A guide to some common eastern Bering Sea snails. Northwest Fisheries Center Processed Report. Kodiak. 27 p.

Motoda S, Minoda T (1974) Plankton of the Bering Sea. In: Hood DW, Kelley EJ, editors. Oceanography of the Bering Sea with emphasis on renewable resources: Institute of Marine Sciences, University of Alaska Fairbanks. pp. 207-241.

Pavlovskii EN (1955) Atlas of the invertebrates of the Far Eastern Seas of the USSR. Academy of Sciences of the USSR Zoological Inst. (Translated from Russian by Israel Program for Sci. Translation, Jerusalem 1966). 457 p.

Pereyra WT, Reeves JE, Bakkala RG (1976) Demersal and shellfish resources of the eastern Bering Sea in the baseline year 1975) NOAA Processed Report NMFS. 619 p.

Stoker SW (1978) Benthic invertebrate macrofauna of the eastern continental shelf of the Bering and Chukchi Seas. Ph.D. Thesis: University of Alaska Fairbanks. 259 p.

Wilimovsky NJ (1974) Fishes of the Bering Sea: The state of existing knowledge and requirements for future effective effort. In: Hood DW, Kelley EJ, editors. Oceanography of the Bering Sea with emphasis on renewable resources: Institute of Marine Sciences, University of Alaska Fairbanks. pp. 243-256.

Arctic Ocean, Beaufort and Chukchi seas

Entry points into the broader scientific literature

Bluhm BA, Gradinger R (2008) Regional variability in food availability for arctic marine mammals. Ecol Appl 18: Supplement S77-S96.

Dunton KH, Weingartner T, Carmack EC (2006) The nearshore western Beaufort Sea ecosystem: Circulation and importance of terrestrial carbon in arctic coastal food webs. Progr. Oceanogr., 71: 362–378.

Grebmeier JM, Cooper LW, Feder HM and Sirenko, B.I (2006) Ecosystem dynamics of the Pacific-influenced northern Bering and Chukchi seas in the Amerasian Arctic. Prog Oceanogr 71: 331-361.

Grebmeier JM, Overland JE, Moore SE, Farley EV, Carmack EC, et al (2006) A major ecosystem shift observed in the northern Bering Sea. Science 311: 1461-1464.

Herman, Y (1989) The Arctic seas. Climatology, oceanography, geology, and biology. New York: Van Nostrand Reinhold. 888 p.

Hopcroft, R, Bluhm, B, and Gradinger, R (eds.) (2008) Arctic Ocean synthesis: Analysis of climate change impacts in the Chukchi and Beaufort seas with strategies for future research. Final report for project 503 for North Pacific Research Board. Institute of Marine Sciences, University of Alaska Fairbanks.

Iken, K. and Konar, B (eds.) Proceedings of the Arctic Biodiversity Workshop: New Census of Marine Life Initiative; 2003; Fairbanks: Alaska Sea Grant College program, University of Alaska Fairbanks. pp. 162 p.

Lane PVZ, Llinas, L, Smith SL, and Pilz, D (2007) Zooplankton distribution in the western Arctic during summer 2002: Hydrographic habitats and implications for food chain dynamics. J Marine Syst 70: 97-103.

MacGinitie, N (1959) Marine Mollusca of Point Barrow, Alaska. Proceedings of the United States National Museum. No. 3412, Vol. 109, pp 59-208.

Piepenburg, D (2005) Recent research on Arctic benthos: common notions need to be revised. Polar Biol 28: 733-755.

Sirenko BI and Gagaev SY (2007) Unusual abundance of macrobenthos and biological invasions in the Chukchi Sea. Russ J Mar Biol 33: 355-364.

Smith WO Jr, Barber DG (2007) Benthic processes in polynas. Elsevier Oceanography Series 74. 363-390 p.

Examples of taxonomic studies and species/taxa lists

Bernard FR (1979) Bivalve mollusks of the western Beaufort Sea. Contrib Sci Natural History Museum, Los Angeles 313: 1-80.

Brodsky KA (1950) Calanoida of the far eastern seas and Polar Basin of the USSR. USSR Academy of Sciences No. 35. (Translated from Russian by Israel Program for Scientific Translations. 1967).

Dunton KH, Schonberg SV (2000) The benthic faunal assemblage of the Boulder Patch kelp community. In: Truett JC, Johnson SR, editors. The natural history of an Arctic oil field. New York: Academic Press. pp. 371-397.

Ellis DV (1960) Marine infaunal benthos in Arctic North America. Arctic Inst. North Am. Tech. Paper 5. 56 p.

Feder HM, Jewett SC, Blanchard AL (2005) Southeastern Chukchi Sea (Alaska) epibenthos. Polar Biol 28: 402-421.

Feder HM, Jewett SC, Blanchard AL (2007) Southeastern Chukchi Sea (Alaska) macrobenthos. Polar Biol 30: 261-275.

Haud C, Kan LB (1961) The Medusae of the Chukchi and Beaufort seas of the Arctic Ocean including the description of a new species of Encodonium (Hydrozoa: Anthomedusae). Arctic Inst. of North Am. Tech. Paper 6. 23 p.

Heifetz J, Wing BL, Stone RP, Malecha PW, Courtney DL (2005) Corals of the Aleutian Islands. Fish Oceanogr 14 (Suppl 1): 131-138.

Horner RA (1985) Sea ice biota. Boca Raton, Fla: CRC Press. 215 p.

Hülsemann K (1963) Radiolaria in Plankton from the Arctic drifting station T-3, including the description of three new species. Arctic Institute of North America Technical Paper 13. 52 p.

Macpherson E (1971) The marine mollusks of Arctic Canada. Publications in Biological Oceanography, No. 3. Natural Museums of Canada, Ottawa. 149 p.

Huntington HP, Moore SE (2008) Assessing the impacts of climate change on Arctic marine mammals. Ecol Appl 18(2) suppl: S1-2.

Johnson SR, Herter DR (1989) The birds of the Beaufort Sea. BP Exploration. Anchorage, Alaska. 372 p.

Kluge GA (1962) Bryozoa of the northern seas of the USSR. Academy of Sciences of the USSR Publication 76. (Translated from Russian in 1974) 711 p.

Sirenko BI (2001) List of species of free-living invertebrates of Eurasian Arctic seas and adjacent deep waters. Explorations of the Fauna of the Seas 51(59).

Stoker SW (1978) Benthic invertebrate macrofauna of the eastern continental shelf of the Bering and Chukchi Seas. Ph.D. Thesis: University of Alaska Fairbanks. 259 p.

General taxonomic guides

Marine seaweeds, maritime plants and lichens

Abbott IA, Hollenberg GJ (1976) Marine algae of California. Stanford, Calif.: Stanford University Press. 827 p.

Druehl LD (2000) Pacific seaweeds: A guide to common seaweeds of the West Coast. Harbour Publishing, BC Canada. 190 p.

Gabrielson PW, Widdowson TB, Lindstrom SC, Hawkes MW, Scagel RF (2000) Keys to the benthic marine algae and seagrasses of British Columbia, Southeast Alaska, Washington and Oregon. University of British Columbia, Department of Botany. 187 p.

Garza DA (2005) Common edible seaweeds in the Gulf of Alaska. Alaska Sea Grant College Program. 57 p.

Lamb A, Hanby BP (2005) Marine life of the Pacific Northwest: A photographic encyclopedia of invertebrates, seaweeds and selected fishes. Madeira Park, British Columbia: Harbour Publishing Co. Ltd. 398 p.

Mondragon J, Mondragon J (2003) Seaweeds of the Pacific Coast. Monterey, Calif.: Sea Challengers. 96 p.

O'Clair RM, Lindstrom SC, Brodo IR (1996) Southeast Alaska's rocky shores: Seaweeds and lichens. Auke Bay, Alaska: Plant Press. 152 p.

O'Clair RM, Lindstrom SC (2000) North Pacific seaweeds. Auke Bay, Alaska: Plant Press. 159 p.

Scagel RF, Gabrielson PW, Garbary DJ, Golden L, Hawkes MW, et al. (1989) A synopsis of the benthic marine algae of British Columbia, Southeast Alaska, Washington and Oregon. Phycological Contribution no. 3. University of British Columbia, Department of Botany. 532 pp. Reprinted 1993 with minor changes and corrections.

Wilce RT (1994) The Arctic subtidal as habitat for macrophytes. In: Lobban S, Harrison PJ, editors. Seaweed ecology and physiology. Cambridge: Cambridge University Press. pp. 89–92.

Seaweeds of Alaska Web site (<http://www.seaweedsofalaska.com/>)

General invertebrates

Austin WC, Deutsch MM (1978) Marine biota of the N.E. Pacific: A bibliography emphasizing systematic and distribution. Cowichan Bay, BC: Khoyatan Marine Laboratory. 350 p.

Clark RN (2006) Field guide to the benthic marine invertebrates of Alaska's shelf and upper slope taken by NOAA/NMFS/AFSC/ RACE Division trawl surveys. 302 pp.

Field CM, Field CJ (1999) Alaska’s seashore creatures. A guide to selected marine invertebrates. Portland, Ore.: Alaska Northwest Books. 94 p.

Gotshall DW (1994) Guide to marine invertebrates. Alaska to Baja California. Monterey, Calif.: Sea Challengers. 105 p.

Kozloff EN (1996) Marine invertebrates of the Pacific Northwest, revised. Seattle/London: University of Washington Press. 539 p.

Lamb A, Hanby BP (2005) Marine life of the Pacific Northwest: A photographic encyclopedia of invertebrates, seaweeds and selected fishes. Madeira Park, British Columbia: Harbour Publishing Co. Ltd. 398 p.

Pavlovskii EN (1955) Atlas of the invertebrates of the far eastern seas of the USSR. Academy of Sciences of the USSR Zoological Inst. (Translated from Russian by Israel Program for Sci. Translation, Jerusalem 1966). 457 p.

Ushakov PV (1952) Chukchi Sea and its bottom fauna. Krainii severo-vostok Soyuza SSR. Fauna I flora Chukotskogo moray (Extreme southeast of the USSR, Fauna and Flora of the Chukchi Sea), V. 2: 5-83 [In Russian].

Vassilenko SV, Petryashov VV (eds.) 2009) Illustrated keys to free-living invertebrates of Eurasian Arctic seas and adjacent deep waters, Vol. 1. Rotifera, Pycnogonida, Cirripedia, Leptostraca, Mysidacea, Hyperiidea, Caprellidea, Euphausiasea, Dendrobranchiata, Pleocyemata, Anomura, and Brachyura. Alaska Sea Grant, University of Alaska Fairbanks. 186 p.

Bryozoa

Kluge GA (1962) Bryozoa of the northern seas of the USSR. Academy of Sciences of the USSR Publication 76. (Translated from Russian in 1974) 711 p.

Robertson A (1910) Bryozoans. Harriman Alaska Series Vol. XI: Smithsonian Institution. pp. 251.

Echinoderms

D’yakonov AM (1923) Fauna of Russia and adjacent countries. Vol. 1: Echinodermata, Echinoidea. (Translated from Russian by Israel Program for Scientific Translations, Jerusalem 1969) 265 p.

Grainger EH (1966) Sea stars (Echinodermata: Asteroidea) of Arctic North America. Fisheries Research Board of Canada Ottawa Bulletin No. 152, 70 p.

Lambert P (1997) Sea cucumbers of British Columbia, Southeast Alaska, and Puget Sound. Royal British Columbia Museum. UBC Press, Vancouver. 166 p.

Lambert P (2000) Sea stars of British Columbia, Southeast Alaska, and Puget Sound. Royal BC Museum Handbook. UBC Press. Vancouver. 186 p.

Lambert P, Austin WC (2007) Brittle stars, sea urchins, and feather stars of British Columbia, Southeast Alaska, and Puget Sound. Royal BC Museum Handbook. Victoria. 150 pp.

Verrill AE (1914) Monograph of the shallow-water starfishes of the North Pacific Coast from the Arctic Ocean to California. Harriman Alaska Series Vol. XIV: Smithsonian Institution. 408 p.

Copepods

Brodsky KA (1950) Calanoida of the far eastern seas and Polar Basin of the USSR. USSR Academy of Sciences No. 35. (Translated from Russian by Israel Program for Scientific Translations. 1967).

Brodsky KA (1957) The copepod fauna (Calanoida) and zoogeographic zonation of the North Pacific and adjacent waters. Izd. Akad. Nauk SSSR, Leningrad.

Brodsky KA (1983) Copepod Crustacea (Copepoda: Calanoida) of the seas of the USSR and adjacent waters (Part 1). Zoological Institute of the Academy of Sciences of the USSR, Leningrad.

Gardner GA, Szabo I (1982) British Columbia pelagic marine Copepoda: an identification manual and annotated bibliography. Can. Spec. Pub. Fish. Aquat. Sci. 62, I-536.

Pteropods

Spoel S van der, Newman LJ, Estep K.W (1996) Pelagic mollusks of the world. ETI Bioinformatics Series.

Mysidacea and Euphausiacea

Kathman RD, Austin WC, Saltman JC, Fulton JD (1986) Identification manual to the Mysidacea and Euphausiacea of the Northeast Pacific. Can. Spec. Pub. Fish. Aquat. Sci. 93, 1-411.

Nemertians

Coe WR (1910) Nemertians. Harriman Alaska Series Vol. XI: Smithsonian Institution. pp. 251.

Mollusks

Abbot RT (1974) American seashells: The marine Mollusca of the Atlantic and Pacific coasts of North America. Second edition. New York: Van Nostrand Reinhold. 663 p.

Baxter R (1983) Mollusks of Alaska. Alaska Dept of Fish and Game. 77 p.

Bernard FR (1979) Bivalve mollusks of the western Beaufort Sea. Contrib Sci Natural History Museum, Los Angeles 313: 1-80.

Foster NR (1991) Intertidal bivalves. A guide to the common marine bivalves of Alaska. University of Alaska Press. 152 p.

MacGinitie N (1959) Marine Mollusca of Point Barrow, Alaska. Proceedings of the United States National Museum. No. 3412, Vol. 109, pp 59-208.

MacIntosh RA (1976) A guide to some common eastern Bering Sea snails. Northwest Fisheries Center Processed Report. Kodiak. 27 p.

Macpherson E (1971) The marine mollusks of Arctic Canada. Publications in Biological Oceanography, No. 3. Natural Museums of Canada, Ottawa. 149 p.

Cnidaria and Ctenophora

Cairns SD, Calder DR, Brinckmann-Voss A, Castro CB, Pugh PR, et al. (1991) Common and scientific names of aquatic invertebrates from the United States and Canada: Cnidaria and Ctenophora. Am. Fisheries Society Special Publication 22. 79 p.

Polychaeta

Zhirkov IA (2001) Polychaeta of the Arctic Ocean. Yanus-K, Moscow, 632 p (in Russian).

Crabs

Donaldson WE, Byersdorfer SC (2005) Biological field techniques for lithodid crabs. Alaska Sea Grant College Program, University of Alaska. 82 p.

Garth JS, Stephenson W (1966) Brachyura of the Pacific Coast of America. Brachyrhyche: Portunidae. Allan Hancock Monographs in Mar. Bio. 1. 154 p.

Jadamec LS, Donaldson WE, Cullenberg, P (1999) Biological field techniques for Chionoecetes crabs. Univ. of Alaska Sea Grant AK-SG-99-02. 80 p.

Jensen GC (1995) Pacific Coast crabs and shrimps. Monterey, Calif.: Sea Challengers. 87 p.

Fishes

Allen MJ, Smith GB (1988) Atlas and zoogeography of common fishes in the Bering Sea and Northeastern Pacific. NOAA Technical Report NMFS 66: 115-133.

Eschmeyer WN, Herald ES, Hammann, H, Smith KP (1984) A field guide to Pacific coast fishes of North America from the Gulf of Alaska to Baja California. Boston: Houghton-Mifflin. 336 p.

Hart, JL (1973) Pacific fishes of Canada. Fish Res Brd Can Bull 180: 740.

Kessler, D.W (1985) Alaska’s saltwater fishes and other sea life. Anchorage: Alaska Northwest Publishing Company. 359 p.

Kramer DE, O’Connell VM (2003) Guide to northeast Pacific rockfishes genera *Sebastes* and *Sebastolobus*. Alaska Sea Grant Marine Advisory Bulletin No. 25: 78 p.

Krame DE, Barss WH, Paust BC, Bracken BE (2008) Guide to northeast Pacific flatfishes. Alaska Sea Grant Marine Advisory Bulletin No. 47: 106 p.

Lamb A, Hanby BP (2005) Marine life of the Pacific Northwest: A photographic encyclopedia of invertebrates, seaweeds and selected fishes. Madeira Park, British Columbia: Harbour Publishing Co. Ltd. 398 p.

Lee RS (1979) Whitefish identification guide. Fairbanks: University of Alaska Sea Grant College Program. 169 p.

Love M, Thorsteinson L (2002) The rockfishes of the northeast Pacific. Berkeley: University of California Press. 416 p.

Mecklenburg CW, Mecklenburg TA, Thorsteinson LK (2002) Fishes of Alaska. Bethesda, Md.: American Fisheries Society. 1116 p.

Stevenson DE, Orr JW, Hoff GR, McEachran JD (2007) Field guide to sharks, skates, and ratfish of Alaska. Alaska Sea Grant College Program, University of Alaska Fairbanks. 77 p.

Seabirds and sea ducks

Gabrielson IN, Lincoln FC (1959) The birds of Alaska. Stackpole Company, Harrisburg, Pa., and Wildlife Management Institute, Washington, D.C. 992 p.

Haley D (ed.) (1984) Seabirds of eastern North Pacific and Arctic waters. Seattle: Pacific Search Press. 214 p.

Also see lists at <http://alaska.fws.gov/mbsp/mbm/seabirds/species_list.htm> and <http://seaduckjv.org/>.

Marine mammals

Wynne K (2007) Guide to marine mammals of Alaska. Mar Advis Bull 44: 75.
