# Supplementary material for: An Overview of Marine Biodiversity in United States Waters
Source: PLoS One. 2010 Aug 2;5(8):e11914. doi: 10.1371/journal.pone.0011914 (PMC2914028; doi:10.1371/journal.pone.0011914)
Supplement: Text S3 — List of Contributors to the Harte Research Institute for Gulf of Mexico Studies Gulf of Mexico Biodiversity Project [Felder DL, Camp DK (eds) (2009) Gulf of Mexico Origin, Waters, and Biota. Volume 1, Biodiversity. College Station, Texas: Texas A&M University Press. 1384 p.] (0.12 MB DOC) [file pone.0011914.s010.doc]

**Text S3.** **List of Contributors to the Harte Research Institute for Gulf of Mexico Studies Gulf of Mexico Biodiversity Project** [Felder DL, Camp DK (eds) (2009) Gulf of Mexico Origin, Waters, and Biota. Volume 1, Biodiversity. College Station, Texas: Texas A&M University Press. 1384 p.]

**Pål Aas**

Department of Biology, University of Bergen

Box 7800

Bergen N-5020 Norway

Pal.Aas@student.uib.no

**Lophogastrida (Co-author)**

**Fernando Álvarez**

Colección Nacional de Crustáceos

Instituto de Biología, UNAM

Apartado Postal 70-153

México, D.F. 04510, México

falvarez@servidor.unam.mx

**Decapoda (Co-author); Stomatopoda (Co-author)**

**Omar M. Amin**

Institute of Parasitic Diseases

P.O. Box 28372

Tempe, AZ 85285-8372, U.S.A.

OmarAmin@aol.com

**Acanthocephala (Co-author)**

**Gary Anderson**

Department of Biological Sciences

University of Southern Mississippi

Hattiesburg, MS 39406, U.S.A.

gary.anderson@usm.edu

**Tanaidacea (Co-author)**

**Erick R. Baqueiro Cárdenas**

CICATA-IPN-Centro de Investigación en Ciencia Aplicada y Tecnología Avanzada, IPN

Km. 14.5 Carr. Tampico Puerto Industrial, Altamira, Tamaulipas, C.P. 89600 México

toshaar@yahoo.com

**Mollusca Introduction (Co-author)**

**Noe Barrera**

Center for Coastal Studies

Texas A&M University-Corpus Christi

6300 Ocean Drive

Corpus Christi, TX 78412, U.S.A.

Noe.barrera@tamucc.edu

**Mollusca Introduction (Co-author)**

**Frederick M. Bayer**

Department of Invertebrate Zoology

Smithsonian Institution-NMNH

P.O. Box 37012, MRC-163, W-329

Washington, D.C. 20013-7012, U.S.A.

**Octocorallia (Co-author)**

**Eugene M. Burreson**

Virginia Institute of Marine Sciences

College of William and Mary

P.O. Box 1346

Gloucester Point, VA 23062, U.S.A.

gene@vims.edu

**Marine leeches (Author)**

**Stephen D. Cairns**

Department of Invertebrate Zoology

Smithsonian Institution-NMNH

P.O. Box 37012, MRC-163, W-329

Washington, D.C. 20013-7012, U.S.A.

cairns.stephen@si.edu

**Cnidaria Introduction (Author); Octocorallia (Author); Scleractinia (Author); Hydroids (Co-author)**

**Dale R. Calder**

Department of Natural History

Royal Ontario Museum

100 Queen’s Park

Toronto, Ontario Canada M55 2C6

dalec@rom.on.ca

**Hydroids (Author)**

**Chris B. Cameron**

Sciences Biologiques

University of Montreal

C. P. 6128, Succ. Centre-ville

Montreal, QC, Canada H3C 3J7

ccameron@bms.bc.ca

**Hemichordata (Author); Cephalochordata (Author)**

**David K. Camp**

11990 68th Avenue

Seminole, FL 33772-6107, U.S.A.

campdave@tampabay.rr.com

**Introduction (Co-author); Stomatopoda (Co-author); Volume (Co-editor)**

**Iván A. Castellanos**

ECOSUR, Unidad Chetumal Av.

Centenario Km.5.5, A.P. 424

Chetumal, Quintana Roo, 77000, México

ivancast@ecosur.mx

**Euphausiacea (Author); Appendicularia (Author)**

**Laura Celis**

Instituto de Ciencias del Mar y Limnología

Unidad Académica Puerto-UNAM

P.O. Box 1152

77501 Cancún, Quintana Roo, México

lcelis@mar.icmyl.unam.mx

**Medusozoa (Co-author)**

**Luciano Chiaverano**

Dauphin Island Sea Lab

University of South Alabama

101 Bienville Blvd.

Dauphin Island, AL 36528, USA

ichiaverano@www.disl.org

**Medusozoa (Co-author)**

**C. Allan Child**

8008 Beech Tree Rd., #2929

Bethesda, MD 20817, U.S.A.

Allanchild@comcast.net

**Pycnogonida (Author)**

**Andrei Chistoserdov**

Department of Biology

University of Louisiana at Lafayette

P.O. Box 42451

Lafayette, LA 70504-2451, U.S.A.

ayc6160@louisiana.edu

**Prokaryotes (Author)**

**Tae Oh Cho**

Department of Biology

University of Louisiana at Lafayette

P.O. Box 42451

Lafayette, LA 70504-2451, U.S.A.

txc7221@louisiana.edu

**Chlorophyta, Phaeophyta, & Rhodophyta (Co-author)**

**Luz Elena Mateo Cid**

Laboratorio de Ficología, Departamento de Botánica

Escuela Nacional de Ciencias Biológicas

Instituto Politécnico Nacional

Carpio y Plan de Ayala Col. Sto. Tomás

México, D.F. 11340, México

luzecyd@yahoo.com.mx

**Chlorophyta, Phaeophyta, & Rhodophyta (Co-author)**

**John C. Clamp**

Department of Biology

North Carolina Central University

Durham, NC 27707, U.S.A.

jclamp@nccu.edu

**Ciliated Protozoa (Co-author)**

**D. Wayne Coats**

Smithsonian Environmental Research Center

647 Contees Wharf Road

Edgewater, MD 21037, U.S.A.

coatsw@si.edu

**Ciliated Protozoa (Author)**

**Linda Cole**

Smithsonian Institution-NMNH, MRC-163

P.O Box 37012

Washington, D.C. 20013-7012, U.S.A.

colel@si.edu

**Tunicates (Author)**

**Joshua O. Cook**

Gulf Coast Research Laboratory

University of Southern Mississippi

703 East Beach Drive

Ocean Springs, MS 39564, U.S.A.

josh.cook@usm.edu

**Trematoda (Co-author)**

**Marymegan Daly**

Department of Evolution, Ecology and Evolutionary Biology

Ohio State University

Columbus, OH 43210, U.S.A.

daly.66@osu.edu

**Actiniaria, Corallimorpharia, and Zoanthidea (Co-author)**

**Sylvia A. Earle**

Harte Research Institute for Gulf of Mexico Studies

Texas A&M University-Corpus Christi

6300 Ocean Drive

Corpus Christi, TX 78412, U.S.A.

**Chlorophyta, Phaeophyta, & Rhodophyta (Co-author)**

**Elva Escobar-Briones**

Universidad Nacional Autónoma de México

Instituto de Ciencias del Mar y Limnología

Unidad Académica Sistemas Oceanográficos y Costeros

A. P. 70-305, Ciudad Universitaria, 04510 D.F México

escobri@mar.icmyl.unam.mx

**Amphipoda (Co-author)**

**José Espinosa**

Instituto de Oceanología, Academia de Ciencias de Cuba

Avenida 1a no. 18406, E. 184 y 186

Municipio Playa

Ciudad de La Habana, Cuba

espinosa@oceano.inf.cu

**Mollusca Introduction (Co-author)**

**Kristian Fauchald**

Department of Invertebrate Zoology

National Museum of Natural History

Smithsonian Institution

NHB MRC 0163, P.O. Box 37012

Washington, D.C. 20013-7012, U.S.A.

Fauchald@si.edu

**Polychaeta (Author)**

**Vestimentifera and Pogonophora (Author)**

**Maria A. Faust**

U.S. National Herbarium

Smithsonian Institution-NMNH

Washington, D.C. 20560, U.S.A.

faustm@si.edu

**Dinoflagellates (Co-author)**

**Daphne Gail Fautin**

Department of Ecology and Evolutionary Biology, and

Natural History Museum

University of Kansas

Lawrence, KS 66045, U.S.A.

fautin@ku.edu

**Cnidaria Introduction (Co-author); Actiniaria, Corallimorpharia and Zoanthidea (Author)**

**Darryl L. Felder**

University of Louisiana at Lafayette

Department of Biology and Laboratory for Crustacean Research

P.O. Box 42451

Lafayette, LA 70504-2451, U.S.A.

dlf4517@louisiana.edu

**Introduction (Author); Decapoda (Author), Volume (Co-editor)**

**John W. Fleeger**

Department of Biological Sciences

Louisiana State University

Baton Rouge, LA 70803, U.S.A.

zoflee@lsu.edu

**Free-living Copepoda (Co-author)**

**Suzanne Fredericq**

Department of Biology

University of Louisiana at Lafayette

P.O. Box 42451

Lafayette, LA 70504-2451, U.S.A.

slf9209@louisiana.edu

**Chlorophyta, Phaeophyta, & Rhodophyta (Author); Diatoms (Co-author)**

**Julio C. Gallardo**

Centro de Ecología y Pesquerías

Universidad Veracruzana

Calle Hidalgo 617, Col. Río Jamapa, Boca del Río

C.P. 94290, Veracruz, México

**Aves (Co-author)**

**Emilio F. García**

University of Louisiana at Lafayette

P.O. Box 42451

Lafayette, LA 70504-2451, U.S.A.

efg2112@louisiana.edu

**Mollusca Introduction (Co-author); Gastropoda (Co-author)**

**Rebeca Gasca**

ECOSUR, Unidad Chetumal, A.P. 424

Chetumal, Quintana Roo, 77000, México

Rgasca@ecosur.mx

**Siphonophora (Co-author); Amphipoda (Co-author)**

**F. Raúl Gío Argáez**

Instituto de Ciencias del Mar y Limnología-UNAM

A.P. 70-305

México 04510, México

raulg@mar.icmyl.unam.mx

**Ostracoda Podocopa (Co-author)**

**Stephen R. Gittings**

NOAA, Marine Sanctuaries Division

1305 East-West Highway, Rm. 11642

Silver Spring, MD, 20910, U.S.A.

steve.gittings@noaa.gov

**Cirripedia (Author)**

**Michele Gold-Morgan**

Laboratorio de Ficología, Facultad de Ciencias-UNAM

Circuito Exterior

Coyoacán, México, D.F. 04510, México

mgm@hp.fciencias.unam.mx

**Benthic Cyanoprokaryota (Co-author)**

**A. Catalina Mendoza Gonzáles**

Laboratorio de Ficología, Departamento de Botánica

Escuela Nacional de Ciencias Biológicas

Instituto Politécnico Nacional

Carpio y Plan de Ayala Col. Sto. Tomás

México, D.F. 11340, México

**Chlorophyta, Phaeophyta, & Rhodophyta (Co-author)**

**María C. González**

Department of Botany, Institute of Biology-UNAM

México City, D.F. 04510, México

mcgv@ibiologia.unam.mx

**Marine Fungi (Author)**

**Joseph W. Goy**

Department of Biology, Box 2251

Harding University

Searcy, AR 72149-0001, U.S.A.

jwgoy@harding.edu

**Decapoda (Co-author)**

**Adolfo G. Gracia**

Instituto de Ciencias del Mar y Limnología

Universidad Nacional Autonóma de México

Apartado Postal 70-305

México, D.F. 04510, México

gracia@mar.icmyl.unam.mx

**Stomatopoda (Co-author)**

**Alejandro Granados-Barba**

Centro de Ecología y Pesquerías

Universidad Veracruzana

Calle Hidalgo #617, Col. Río Jamapa, Boca del Río

Veracruz, C.P. 94290, México

agranados1@gmail.com

**Polychaeta (Co-author)**

**C. Fred Gurgel**

Department of Biology

University of Louisiana at Lafayette

P.O. Box 42451

Lafayette, LA 70504-2451, U.S.A.

f_grugel@yahoo.com

**Chlorophyta, Phaeophyta, & Rhodophyta (Co-author)**

**Todd A. Haney**

Department of Ecology and Evolutionary Biology

University of California Los Angeles

Los Angeles, CA 90095, U.S.A.

haney@ucla.edu

**Leptostraca (Co-author)**

**Elizabeth Harrison-Nelson**

Department of Invertebrate Zoology

Smithsonian Institution, NMNH

Washington, D.C., 20013-7012, U.S.A.

nelsone@si.edu

**Myodocopan Ostracoda (Author)**

**Robert R. Haynes**

Department of Biological Sciences

University of Alabama

Tsucaloosa, AL 35478, U.S.A.

rhaynes@bama.ua.edu

**Plants (Co-author)**

**Richard W. Heard**

Department of Coastal Sciences

University of Southern Mississippi

P.O. Box 7000

Ocean Springs, MS 39566-7000, U.S.A.

richard.heard@usm.edu

**Lophogastrida (Co-author); Tanaidacea (Author); Cumacea (Author); Mysida (Co-author); Trematoda (Co-author)**

**David U. Hernández-Becerril**

Instituto de Ciencias del Mar y Limnología-UNAM

México, D.F. 04510, México

dhernand@icmyl.unam.mx

**Dinoflagellates (Co-author)**

**Rosa Ma. Hernández-Flores**

ECOSUR, Unidad Chetumal Av., Centenario Km.5.5

A.P. 424, Chetumal, Quintana Roo, 77000, México

rosamahe@ecosur.mx

**Planktonic Chaetognatha (Author)**

**F. G. Hochberg**

Department of Invertebrate Zoology

Santa Barbara Museum of Natural History

2559 Puesta Del Sol Road

Santa Barbara, CA 93105, U.S.A.

fghochberg@sbnature2.org

**Dicyemida (Co-author)**

**Rick Hochberg**

Smithsonian Marine Station

701 Seaway Drive

Fort Pierce, FL 34949, U.S.A.

Hochberg_Rick@yahoo.com

**Gastrotricha (Author)**

**Matthew D. Hooge**

Department of Biological Sciences

University of Maine

5751 Murray Hall

Orono, ME 04469-5751, U.S.A.

hooge@umit.maine.edu

**Turbellaria (Author)**

**W. Duane Hope**

Department of Invertebrate Zoology

National Museum of Natural History

Smithsonian Institution

Room W212, MRC 163, P.O. Box 37012

Washington, D.C. 20013-7012, U.S.A.

hoped@si.edu

**Free-living Marine Nematoda (Author)**

**Dmitry L. Ivanov**

Zoological Museum

Moscow State University

Bol`shaja Nikitskaja Str. 6

Moscow 125009, Russia

**Aplacophora (Author)**

**Walter C. Jaap**

Florida Fish and Wildlife Research Institute

100 Eighth Avenue SE

St. Petersburg, FL 33701-5095, U.S.A.

walt.jaap@myfwc.com

**Scleractinia (Co-author)**

**Kirsten Jensen**

Department of Ecology and Evolutionary Biology

Natural History Museum

University of Kansas

Lawrence, Kansas 66045, U.S.A.

jensen@ku.edu

**Cestoda (Author)**

**Heather L. Judkins**

College of Marine Science

University of South Florida

St. Petersburg, FL 33701, U.S.A.

hjudkins@marine.usf.edu

**Cephalopoda (Author)**

**Louis S. Kornicker**

Department of Invertebrate Zoology

Smithsonian Institution, NMNH

Washington, D.C. 20013-7012, U.S.A.

kornickl@si.edu

**Myodocopan Ostracoda (Co-author)**

**John N. Kraeuter**

Haskin Shellfish Research Lab

Institute of Marine and Coastal Sciences

Rutgers University

Port Norris, NJ 08349, U.S.A.

kraeuter@hsrl.rutgers.edu

**Scaphopoda (Author)**

**David M. Krayesky**

Department of Biology

University of Louisiana at Lafayette

Lafayette, LA 70504-2451, U.S.A.

dmk8533@louisiana.edu

**Chlorophyta, Phaeophyta, & Rhodophyta (Co-author); Diatoms (Author)**

**Gretchen Lambert**

12001 11th Ave., NW

Seattle, WA 98117, U.S.A.

glambert@fullerton.edu

**Tunicata (Co-author)**

**Judith C. Lang**

P.O. Box 539

Ophelia, VA 22530, U.S.A.

jandl@rivnet.net

**Scleractinia (Co-author)**

**Sara LeCroy**

USM Institute of Marine Sciences

Gulf Coast Research Laboratory

703 East Beach Blvd., P.O. Box 7000

Ocean Springs, MS 39564, U.S.A.

sara.lecroy@usm.edu

**Amphipoda (Author)**

**Rafael Lemaitre**

Department of Invertebrate Zoology

National Museum of Natural History, MRC 163

Smithsonian Institution

Washington, D.C., 20013-7012, U.S.A.

lemaitrr@si.edu

**Decapoda (Co-author)**

**Hilda León-Tejera**

Herbario de la Facultad de Ciencias-UNAM

Circuito Exterior, Coyoacán México, D.F. 04510, México

hlt@hp.fciencias.unam.mx

**Benthic Cyanoprokaryota (Author)**

**Donald H. Les**

Department of Ecology and Evolutionary Biology

University of Connecticut

Storrs, CT 06269-3043, U.S.A.

les@uconn.edu

**Plants (Author)**

**William G. Lyons**

4227 Porpoise Drive SE

St. Petersburg, FL 33705, U.S.A.

w.lyons@knology.net

**Mollusca Introduction (Co-author); Polyplacophora (Author); Bivalvia (Co-author)**

**María Luisa Machain-Castillo**

Instituto de Ciencias del Mar y Limnología

Universidad Nacional Autónoma de México (UNAM)

A.P. 70-305

México, D.F. 04510, México

machain@mar.icmyl.unam.mx

**Foraminifera (Co-author); Podocopan Ostracoda (Co-author)**

**Vania Macías**

Centro de Ecología y Pesquerías

Universidad Veracruzana

Calle Hidalgo 617, Col. Río Jamapa, Boca del Río

Veracruz, C.P. 94290, México

**Aves (Co-author)**

**Rosalie F. Maddocks**

Department of Geosciences

University of Houston

Houston, TX 77204-5007, U.S.A.

RMaddocks@uh.edu

**Podocopan Ostracoda (Author)**

**Christopher L. Mah**

Department of Invertebrate Zoology

Smithsonian Institution-NMNH

Washington, D.C. 20013-7012, U.S.A.

mahc@si.edu

**Echinodermata (Co-author)**

**John C. Markham**

Arch Cape Marine Laboratory

31909 Markham Lane

Arch Cape, OR 97102-0133, U.S.A.

jmarkham@seasurf.net

**Isopoda (Co-author)**

**Joel W. Martin**

Natural History Museum of Los Angeles County

900 Exposition Blvd.

Los Angeles, CA 90007, U.S.A.

jmartin@nhm.org

**Cephalocarida and Mystacocarida (Author); Leptostraca (Author)**

**Frank J. Maturo, Jr.**

Zoology Department

University of Florida

Gainesville, FL 32600, U.S.A.

fjmaturo@zoology.ufl.edu

**Bryozoa (Co-author)**

**John D. McEachran**

Department of Wildlife and Fisheries Sciences

Texas A&M University, TAMU 2258

College Station, TX 77843-2258, U.S.A.

j-mceachran@neo.tamu.edu

**Fishes (Author)**

**Jerry A. McLelland**

Department of Coastal Sciences

University of Southern Mississippi

Gulf Coast Research Laboratory

P.O. Box 7000

Ocean Springs, MS 39564, U.S.A.

jerry.mclelland@usm.edu

**Planktonic Chaetognatha (Co-author)**

**Esther Meave del Castillo**

Departamento de Hidrobiología

Universidad Autónoma Metropolitana-Iztapalapa

Av. San Rafael Atlixco 186

C.P. 55-535, México, D.F. 09340, México

mem@xanum.uam.mx

**Diatoms (Co-author)**

**Kenneth Meland**

Department of Biology

University of Bergen

Box 7800

Bergen N-5020, Norway

Kenneth.Meland@bio.uib.no

**Lophogastrida (Co-author)**

**Charles G. Messing**

Nova Southeastern University Oceanographic Center

8000 North Ocean Drive

Dania Beach, FL 33004, U.S.A.

messingc@nsu.nova.edu

**Echinodermata (Co-author)**

**Paula Mikkelsen**

Paleontological Research Institution

1259 Trumansburg Road, Route 96

Ithaca, NY 14850, U.S.A.

mikkelsen@museumoftheearth.org

**Bivalvia (Co-author)**

**Tina N. Molodtsova**

P.P. Shirshov Institute of Oceanology-RAS

36 Nakhimovskiy prospect

Moscow 117218, Russia

tina@sio.rssi.ru

**Ceriantharia (Author)**

**Paul A. Montagna**

Harte Research Institute for Gulf of Mexico Studies

Texas A&M University-Corpus Christi

6300 Ocean Drive

Corpus Christi, TX 78412, U.S.A.

Paul.Montagna@tamucc.edu

**Free-living Copepoda (Co-author)**

**Gustavo Montejano**

Laboratorio de Ficología, Facultad de Ciencias-UNAM

Circuito Exterior

Coyoacán, México, D.F. 04510, México

gmz@hp.fciencias.unam.mx

**Benthic Cyanoprokaryota (Co-author)**

**Fabio Moretzsohn**

Harte Research Institute for Gulf of Mexico Studies

Texas A&M University-Corpus Christi

6300 Ocean Drive

Corpus Christi, TX 78412, U.S.A.

mollusca@gmail.com

**Mollusca Introduction (Author); Polyplacophora (Co-author); Gastropoda (Co-author); Bivalvia (Co-author)**

**Anthony G. Moss**

Biological Sciences

Auburn University

Auburn, AL 36849, U.S.A.

mossant@auburn.edu

**Ctenophora (Author)**

**Leslie J. Newman**

Marine Biology

Auckland Museum

Private Bag 92018

Auckland, New Zealand

lnewman@aucklandmuseum.com

**Turbellaria (Co-author)**

**Joseph E. Neigel**

Department of Biology

University of Louisiana at Lafayette

Box 42451

Lafayette, LA 70504, U.S.A.

jneigel@louisiana.edu

**Population Genetics/Biogeography (Author)**

**Claus Nielsen**

Natural History Museum of Denmark

University of Copenhagen

Universitetsparken 15

DK-2100 Copenhagen, Denmark

cnielsen@snm.ku.dk

**Entoprocta (Author)**

**Jon L. Norenburg**

Smithsonian Institution, PO Box 37012

Invertebrate Zoology, NMNH, W-216, MRC163

Washington, D.C. 20013-7012, U.S.A.

norenburgj@si.edu

**Nemertea (Author)**

**James N. Norris**

Department of Botany, NHB 166

National Museum of National History

Smithsonian Institution, P.O. Box 37012

Washington, D.C. 20013-7012, U.S.A.

norrisj@si.edu

**Chlorophyta, Phaeophyta, & Rhodophyta (Co-author); Diatoms (Co-author)**

**Alejandro Novelo-Retana**

Departamento de Botánica, Instituto de Biología

Universidad Nacional Autónoma de México

Apartado Postal 70-367

México, D.F. 04510, México

**Plants (Co-author)**

**Dennis M. Opresko**

Life Sciences Division

Oak Ridge National Laboratory

1060 Commerce Park

Oak Ridge, TN 37830, U.S.A.

opreskodm@ornl.gov

**Antipatharia (Author)**

**Jesús Ortea**

Departamento de Organismos y Sistemas

Laboratorio de Zoología

Universidad de Oviedo Jesús Arias de Velasco sin

33005 Oviedo, Asturias, Spain

jortea@correo.uniovi.es

**Mollusca Introduction (Co-author)**

**Manuel Ortiz Touzet**

Universidad de la Habana, Centro de Investigaciones Marinas

Calle 16 #114, entre l era y 3 era Miramar Playa

Ciudad de la Habana, Cuba

ortiztouzet@yahoo.com

**Amphipoda (Co-author); Stomatopoda (Co-author)**

**Robin M. Overstreet**

Gulf Coast Research Laboratory

University of Southern Mississippi

703 East Beach Dr.

Ocean Springs, MS 39564, U.S.A.

robin.overstreet@usm.edu

**Dicyemida (Author); Trematoda (Author)**

**David L. Pawson**

Department of Invertebrate Zoology

Smithsonian Institution-NMNH

Washington, D.C. 20013-7012, U.S.A.

pawsond@si.edu

**Echinodermata (Author)**

**Carla Piantoni**

Department of Invertebrate Zoology

National Museum of Natural History

MRC 163, Smithsonian Institution

Washington, D.C. 20013-7012, U.S.A.

dietricc@si.edu

**Porifera (Co-author)**

**John F. Pilger**

Department of Biology

Agnes Scott College

141 East College Avenue

Decatur, GA 30030-3770, U.S.A.

jpilger@agnesscott.edu

**Echiura (Author)**

**William J. Poly**

SCDNR, Fisheries Research

1921 Vanboklen Road

Eastover, SC 29044, U.S.A.

wpoly@calacademy.org

**Branchiura (Author)**

**W. Wayne Price**

Department of Biology

University of Tampa

Tampa, FL 33606, U.S.A.

wprice@ut.edu

**Lophogastrida (Author); Mysida (Author)**

**Philip R. Pugh**

National Oceanography Centre

Southampton, S014 3ZH, U.K.

prp@noc.soton.ac.uk

**Siphonophora (Author)**

**Marjorie L. Reaka**

Department of Biology

University of Maryland

College Park, MD 20742, U.S.A.

mlreaka@umd.edu

**Stomatopoda (Author)**

**Martha Reguero**

Instituto de Ciencias del Mar y Limnología-UNAM

Apartado Postal 70-305

México, D.F. 04510, México

reguero@mar.icmyl.unam.mx

**Mollusca Introduction –Co-author**

**Mary E. Rice**

Smithsonian Institution

Smithsonian Marine Station at Fort Pierce

701 Seaway Drive

Fort Pierce, FL 34949, U.S.A.

rice@si.edu

**Sipuncula (Author)**

**Daniel Roccatagliata**

Departamento de Biodiversidad y Biología Experimental

Facultad de Ciencias Exactas y Naturales

Universidad de Buenos Aires, C1428EHA

Buenos Aires, Argentina

rocca@bg.fcen.uba.ar

**Cumacea (Co-author)**

**Frank A. Romano, III**

Department of Biology

Jacksonville State University

700 Pelham Road North

Jacksonville, AL 36265-1602, U.S.A.

fromano@jsu.edu

**Tardigrada (Author)**

**Clyde Roper**

Invertebrate Zoology, MRC 118

National Museum of Natural History

Smithsonian Institution

Washington, D.C. 20013-7012, U.S.A.

gsquidinc@verizon.net

**Cephalopoda (Co-author)**

**Gary Rosenberg**

Academy of Natural Sciences

1900 Benjamin Franklin Parkway

Philadelphia, PA 19103, U.S.A.

rosenberg@ansp.org

**Gastropoda (Author); Bivalvia (Co-author)**

**Klaus Rützler**

Department of Invertebrate Zoology

National Museum of Natural History

MRC 163, Smithsonian Institution

Washington, D.C. 20013-7012, U.S.A.

ruetzler@si.edu

**Porifera (Author)**

**Guillermo Salgado-Maldonado**

Universidad Nacional Autónoma de México

Instituto de Biología, Departamento de Zoología

Laboratorio de Helmintología,

Apartado Postal 70-153

México, D.F. 04510, México

gsalgado@mail.ibiologia.unam.mx

**Acanthocephala (Author)**

**Scott Santagata**

Smithsonian Environmental Research Center

P.O. Box 28, 647 Contees Wharf Road

Edgewater, MD 21037, U.S.A.

scott_santagata@hotmail.com

**Phoronida (Author); Brachiopoda (Author)**

**Thomas C. Shirley**

Harte Research Institute for Gulf of Mexico Studies

Texas A&M University-Corpus Christi

6300 Ocean Drive, Unit 5869

Corpus Christi, TX 78412-5869, U.S.A.

thomas.shirley@tamucc.edu

**Priapulida (Author); Loricifera (Author);**

**Kinorhyncha (Author)**

**David J. Schmidly**

Scholes Hall, Building 10

University of New Mexico

Albuquerque, NM 87131-0001, U.S.A.

unmpres@unm.edu

**Marine Mammals (Author)**

**Amélie H. Scheltema**

Woods Hole Oceanographic Institution

Redfield 1-34 MS #34

Woods Hole, MA 02543, U.S.A.

aschetema@whoi.edu

**Aplacophora (Co-author)**

**Marilyn Schotte**

Department of Invertebrate Zoology

Smithsonian Institution-NMNH, NHB 163, P.O. Box 37012 Washington, D.C. 20013-7012, U.S.A.

schottem@si.edu

**Isopoda (Author)**

**Lourdes Segura-Puertas**

Unidad Académica Puerto Morales

Instituto de Ciencias del Mar y Limnología, UNAM

P.O. Box 1152

Cancún, Quintana Roo, 77501, México

lsegura@mar.icmyl.unam.mx

**Medusozoa (Author)**

**Barun K. Sen Gupta**

Department of Geology and Geophysics

Louisiana State University

Baton Rouge, LA 70803, U.S.A.

glbarun@lsu.edu

**Foraminifera (Author)**

**Lorene E. Smith**

Museum of Natural Science

Department of Geology and Geophysics

Louisiana State University

Baton Rouge, LA 70803, U.S.A.

lsmit18@lsu.edu

**Foraminifera (Co-author)**

**Rob W. M. van Soest**

Zoological Museum

University of Amsterdam

P.O. Box 94766-1090, GT

Amsterdam, The Netherlands

soest@science.uva.nl

**Porifera (Co-author)**

**Francisco A. Solís-Marín**

Laboratorio de Sistemática y Ecología de Equinodermos

Instituto de Ciencias del Mar y Limnología-UNAM

México, D.F., México

fasolis@icmyl.unam.mx

**Echinodermata (Co-author)**

**Vivianne Solís-Weiss**

Instituto de Ciencias del Mar y Limnología-UNAM

Circuito Escoral Exterior

C.U. Mexico, D.F. 04510, México

solisw@mar.icmyl.unam.mx

**Polychaeta (Co-author)**

**Martin V. Sørensen**

Ancient DNA and Evolution Group

Biological Institute

University of Copenhagen

Universitetsparken 15

DK-2100 Copenhagen, Denmark

mvsorensen@bi.ku.dk

**Gnathostomulida (Author); Rotifera (Author)**

**Karen A. Steidinger**

Florida Institute of Oceanography

Florida Fish and Wildlife Conservation Commission

Fish and Wildlife Research Institute

St. Petersburg, FL 33701, U.S.A.

ksteidin@tampabay.rr.com

**Dinoflagellates (Author)**

**Wolfgang Sterrer**

Bermuda Natural History Museum

Flatts, FLBX, Bermuda

westerrer@gov.bm

**Gnathostomulida (Co-author)**

**Ana María Suárez**

Centro de Investigaciones Marinas, Universidad de la Habana

Calle 16, no. 14, Playa, C.P. 11300

Ciudad la Habana, Cuba

ficologia2005@cim.uh.cu

**Chlorophyta, Phaeophyta, & Rhodophyta (Co-author)**

**Eduardo Suárez-Morales**

ECOSUR, Unidad Chetumal, A.P. 424

Chetumal, Quintana Roo, 77000, México

esuarez@ecosur.mx

**Free-living Copepoda (Author); Euphausiacea (Co-author); Appendicularia (Co-author); Planktonic Chaetognatha (Co-author)**

**John W. Tunnell, Jr.**

Harte Research Institute for Gulf of Mexico Studies

Texas A&M University-Corpus Christi

6300 Ocean Drive, Unit 5869

Corpus Christi, TX 78412-5869, U.S.A.

Wes.Tunnell@tamucc.edu

**Introduction (Co-author); Brachiopoda (Co-author); Mollusca Introduction (Co-author)**

**Donna Turgeon**

8701 Running Fox Court

Fairfax Station, VA 22039, U.S.A.

donna.turgeon@cox.net

**Bivalvia (Author)**

**Doris Vance**

Department of Invertebrate Zoology

National Museum of Natural History

Smithsonian Institution

Washington, D.C. 20013-7012, U.S.A.

vanced@si.edu

**Echinodermata (Co-author)**

**Ana Rosa Vázquez-Bader**

Instituto de Ciencias del Mar y Limnología

Universidad Nacional Autonóma de México

Apartado Postal 70-305

México, D.F. 04510, México

**Stomatopoda (Co-author)**

**Michael Vecchione**

NMFS National Systematics Laboratory

National Museum of Natural History

Washington, D.C. 20013-7012, U.S.A.

VecchioneM@si.edu

**Cephalopoda (Co-author)**

**Enriqueta Velarde**

Unidad de Investigación de Ecología de Pesquerías, Universidad Veracruzana

Calle Hidalgo 617, Col. Río Jamapa, Boca del Río, C.P. 94290

Veracruz, México

enriqueta_velarde@yahoo.com

**Aves (Author)**

**George D. F. Wilson**

Australian Museum

6 College Street

Sydney 2000, NSW, Australia

BuzW@austmus.gov.au

**Isopoda (Co-author)**

**Ignacio Winfield**

Laboratorio de Crustáceos

FES-Iztacala UNAM,

A.P. 314, Tlalnepantla, Estado de México 54090, México

ignacioc@servidor.unam.mx

**Amphipoda (Co-author)**

**Judith E. Winston**

Virginia Museum of Natural History

21 Starling Avenue

Martinsville, VA 24112, U.S.A.

Judith.winston@vmnh.virginia.gov

**Bryozoa (Author)**

**Bernd Würsig**

Marine Mammal Research Program

Texas A&M University

4700 Avenue U, Building 303

Galveston, TX 77551, U.S.A.

wursigb@tamug.edu

**Marine Mammals (Co-author)**

**Eugenia Zamudio**

Departamento de Hidrobiología

Universidad Autónoma Metropolitana-Iztapalapa

Av. San Rafael Atlixco 186.P. 55-535

México, D.F. 09340, México

mem@xanum.uam.mx

**Diatoms (Co-author)**

**George R. Zug**

Smithsonian Institution-NMNH

washington, D.C. 20013-7012, U.S.A.

zugg@si.edu

**Reptilia (Author)**
