# Supplementary material for: An Overview of Marine Biodiversity in United States Waters
Source: PLoS One. 2010 Aug 2;5(8):e11914. doi: 10.1371/journal.pone.0011914 (PMC2914028; doi:10.1371/journal.pone.0011914)
Supplement: Text S4 — California Current Large Marine Ecosystem Contributing Taxonomic Experts (0.03 MB DOC) [file pone.0011914.s011.doc]

**Text S4. California Current Large Marine Ecosystem Contributing Taxonomic Experts**

*CHLOROPHYTA, PHAEOPHYTA, RHODOPHYTA*

Kathy Ann Miller, University Herbarium, 1001 Valley Life Sciences Building #2465, University of California, Berkeley, California 94720 USA

<mailto:kathyannmiller@berkeley.edu>; <http://ucjeps.berkeley.edu/californiaseaweeds.html>

*FORAMINIFERA*

Mary McGann, Research Geologist, Marine Microbiology, U.S. Geological Survey, Coastal and Marine Geology Team, M/S 999, 345 Middlefield Road, Menlo Park, California 94025 USA

<mailto:mmcgann@usgs.gov>

*CNIDARIA*

Daphne Fautin, Professor of Ecology and Evolutionary Biology, Curator Natural History Museum and Biodiversity Research Center, 1200 Sunnyside Avenue, University of Kansas, Lawrence, Kansas 66045-7534 USA

<mailto:fautin@ku.edu>

*NEMERTEA*

Svetlana Maslakova, Assistant Professor, University of Oregon, Oregon Institute of Marine Biology, P.O. Box 5389, 63466 Boat Basin Road, Charleston, Oregon 97420 USA

<mailto:svetlana@uoregon.edu>; [www.uoregon.edu/~svetlana/](../www.uoregon.edu/~svetlana/)

Jon Norenburg, Research Zoologist, Curator of Worms, National Museum of Natural History, Smithsonian Institution, P.O. Box 37012, MRC 163, Washington, District of Columbia 20013-7012 USA

[norenburgj@si.edu](mailto:norenburgj@si.edu)

*CYCLIOPHORA, ANNELIDA, SIBOGLINIDAE*

Greg Rouse, Professor and Curator, Scripps Institution of Oceanography, University of California at San Diego, 9500 Gilman Drive, La Jolla, California 92093-0202 USA

<mailto:grouse@ucsd.edu>

*Crustacea, Chelicerata (non-arachinid)*

Mary Wicksten, Professor of Biology, Texas A&M University, Department of Biology, 3258 TAMU, College Station, Texas 77843-3258 USA

[wicksten@mail.bio.tamu.edu](mailto:wicksten@mail.bio.tamu.edu)

*ECHINODERMATA*

Richard J. Mooi, Curator of Echinoderms, Department of Invertebrate Zoology & Geology,

California Academy of Sciences, 55 Music Concourse Drive, San Francisco, California 94118-4503 USA

[rmooi@calacademy.org](mailto:rmooi@calacademy.org)

*PISCES*

James W. Orr, Research Fish Biologist, NOAA Fisheries Service/Alaska Fisheries Science Center, RACE Division, F/AKC-1, 7600 Sand Point Way NE, Seattle, Washington 98115-0070 USA

<mailto:James.Orr@noaa.gov>
